# Brazilian recommendations of mechanical ventilation 2013. Part I

**DOI:** 10.1590/S1806-37132014000400002

**Published:** 2014

## Abstract

Perspectives on invasive and noninvasive ventilatory support for critically ill patients are evolving, as much evidence indicates that ventilation may have positive effects on patient survival and the quality of the care provided in intensive care units in Brazil. For those reasons, the Brazilian Association of Intensive Care Medicine (*Associação de Medicina Intensiva Brasileira* - AMIB) and the Brazilian Thoracic Society (*Sociedade Brasileira de Pneumologia e Tisiologia* - SBPT), represented by the Mechanical Ventilation Committee and the Commission of Intensive Therapy, respectively, decided to review the literature and draft recommendations for mechanical ventilation with the goal of creating a document for bedside guidance as to the best practices on mechanical ventilation available to their members. The document was based on the available evidence regarding 29 subtopics selected as the most relevant for the subject of interest. The project was developed in several stages, during which the selected topics were distributed among experts recommended by both societies with recent publications on the subject of interest and/or significant teaching and research activity in the field of mechanical ventilation in Brazil. The experts were divided into pairs that were charged with performing a thorough review of the international literature on each topic. All the experts met at the Forum on Mechanical Ventilation, which was held at the headquarters of AMIB in São Paulo on August 3 and 4, 2013, to collaboratively draft the final text corresponding to each sub-topic, which was presented to, appraised, discussed and approved in a plenary session that included all 58 participants and aimed to create the final document.

The present recommendations are a joint initiative of the Mechanical Ventilation Committee of the Brazilian Intensive Care Medicine Association (Associação de Medicina Intensiva Brasileira - AMIB) and the Commission of Intensive Therapy of the Brazilian Thoracic Society (Sociedade Brasileira de Pneumologia e Tisiologia - SBPT). 

## Introduction

Invasive or non-invasive mechanical ventilation (MV) must be performed in an adequate and safe manner to avoid the occurrence of ventilation-induced lung injury. Based on physiological principles, evidence collected in laboratory experiments, and randomized clinical or observational studies involving actual patients that were available in the literature, current MV recommendations indicate that ventilatory support should be performed at a tidal volume (Vt) of 6mL/Kg predicted body weight, with a delta between plateau pressure and positive end-expiratory pressure (PEEP) not greater than 15cmH_2_O, and end-expiratory pressure levels sufficient to avoid airway and alveolar collapse and ensure adequate gas exchange. Other recommendations include positioning the patient to guarantee adequate and harmless ventilation (such as prone positioning in cases of severe acute respiratory distress syndrome - ARDS) and the use of advanced support techniques (such as extracorporeal carbon dioxide (CO_2_) removal) in cases of refractory ARDS. The development of increasingly more sophisticated ventilators allow for fine adjustment of sensitivity and include several trigger mechanisms, different inspiratory flow speeds, acceleration, mechanisms for ending inspiratory time, and monitoring options, which enable adjustment of the patient-ventilator synchrony and MV as a function of the patient's disease. In this regard, the possibility of providing differential ventilatory support for restrictive and obstructive conditions stands out. 

For that reason, joint analysis of the available evidence on ventilatory support by Brazilian experts who deal with mechanical ventilation like anesthesiologists, intensivists, pneumonologists, physical therapists, nurses, nutritionists and speech therapists was necessary. Such evidence, taken together with experience gathered by the various specialties, may provide guidance to health care professionals in Brazilian intensive care units (ICU) on how to provide safe and effective respiratory support for patients with respiratory failure, based on the best evidence available, in order to avoid the occurrence of ventilator-associated lung injury. 

Therefore, the aim of the present study was to review the available literature on 29 subtopics related to ventilatory support for individuals with respiratory failure, and following presentation, discussion, and approval at a plenary session including all 58 participating specialists, to present the results in the form of recommendations and suggestions. 

## Methods

Literature available from MEDLINE (2003-2013) and the Cochrane Central Register of Controlled Trials (CENTRAL) was reviewed by specialists with a higher education (intensivists, anesthetists, pulmonary specialists, physical therapists, and nurses) who were distributed in pairs for review of each of the 29 selected subtopics related to non-invasive and invasive ventilatory support for patients with respiratory failure. 

After reviewing the articles available in the literature, each pair answered the questions formulated by the organizing commission (composed by Carmen Silvia Valente Barbas, President of the Committee of Respiratory Failure and Mechanical Ventilation of AMIB, Alexandre Marini Isola, National Coordinator of the Course of MV in ICU - VENUTI, and Augusto Manoel de Carvalho Farias, Coordinator of the Department of Intensive Care of the SBPT) according to criteria previously suggested by other authors.^(^ 1 ^-^ 4 ^)^ Thus, the term recommendation was used when the level of evidence was high, i.e., derived from randomized studies conducted with more than 100 participants, meta-analyses, all-or-nothing effect, or patient safety. The term suggestion was used when the available evidence was weak, i.e., based on observational or case-control studies, case series, or on the experience of specialists to provide guidance for efficient and safe ventilatory support in Brazil. We therefore hoped that these evidence-based recommendations would help to avoid potential deleterious effects associated with inadequate ventilatory support in our patients. 

The 58 participating specialists were requested to answer the proposed questions during an eight-hour session conducted at the Brazilian Intensive Care Medicine Association (*Associação de Medicina Intensiva Brasileira* - AMIB) on August 3, 2013. The answers were formulated based on the evidence available in the literature and on the experience of the specialists and were then presented at a plenary session that included all 58 participating specialists, which was held on August 4, 2013 at AMIB headquarters. During that session, the answers were discussed, modified when needed, voted on, and approved in accordance with the suggestions and observations of the specialists who attended the meeting. 

The reports made by all the pairs of specialists were gathered by the project organizing commission, which revised, formatted and drafted the final document, following the authors' revisions. The document was then printed in the form of a bedside manual of recommendations to be distributed to ICUs all across Brazil, and it was also sent for publication in the Brazilian Journal of Intensive Care (*Revista Brasileira de Terapia Intensiva* - RBTI) and the Brazilian Journal of Pneumology (*Jornal Brasileiro de Pneumologia*).

## Indications for noninvasive and invasive ventilatory support

**Comment - **Mechanical ventilation (MV) totally or partially replaces spontaneous ventilation and is indicated in acute respiratory failure (ARF) or acute exacerbations of chronic respiratory failure. MV promotes improvement of the gas exchange and reduction in the work of breathing. It can be performed in a noninvasive manner by means of an external interface, which usually consists of a face mask, or in an invasive manner through an endotracheal or a tracheostomy tube. Noninvasive ventilation (NIV) consists of the application of inspiratory pressure to ventilate the patient through a nasal/facial interface (inspiratory positive airway pressure (IPAP) and/or pressure support ventilation (PSV)) or of positive expiratory pressure to keep the airway and alveoli open and thus improve oxygenation (expiratory positive airway pressure (EPAP or PEEP)). The continuous positive airway pressure (CPAP) mode consists of the exclusive application of continuous end-expiratory pressure to the airway through a nasal/facial interface, while the patient's ventilation is fully spontaneous. 

Noninvasive positive pressure mechanical ventilation: when to start

**Chart 1:**
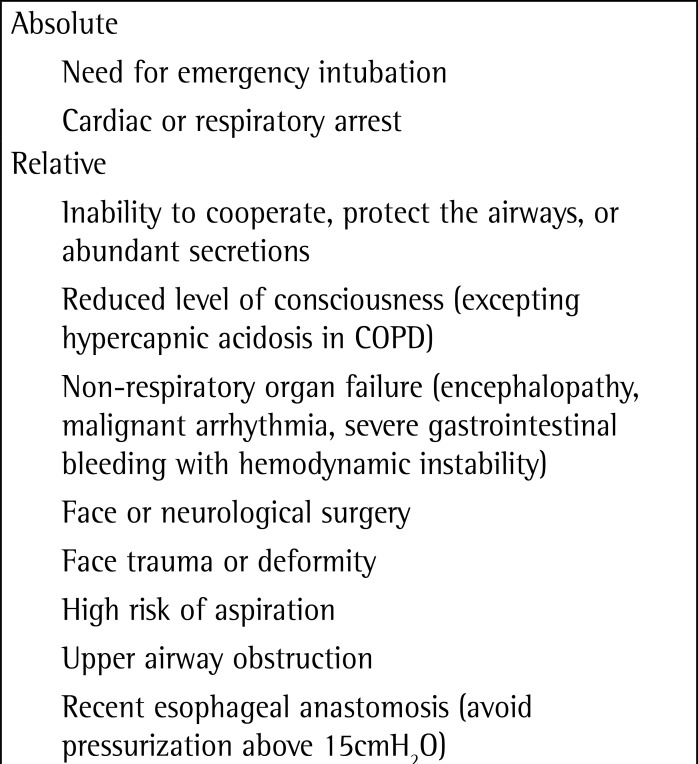
Contraindications to noninvasive ventilation COPD - chronic obstructive pulmonary disease.

**Recommendation -** In the absence of contraindications (Chart 1), patients unable to maintain spontaneous ventilation (minute ventilation >4Lpm, PaCO_2_<50mmHg, and pH>7.25) should start bi-level NIV, with a sufficient inspiratory pressure to maintain adequate ventilation; the goal is to avoid progression to muscle fatigue and/or respiratory arrest.^(^ 5 ^)^

**Suggestion -** NIV may be used in patients with reduced consciousness levels due to hypercapnia in chronic obstructive pulmonary disease (COPD). The level of consciousness should clearly improve one or two hours after beginning NIV.^(^ 5 ^,^ 6 ^)^

**Recommendation -** Patients who deteriorate or do not improve should be immediately intubated due to risk of loss of lower airway protection and respiratory arrest.^(^ 5 ^)^

### Noninvasive positive pressure mechanical ventilation: when to discontinue

**Recommendation -** Use of NIV should be monitored at bedside by a health care professional within thirty minutes to two hours. For NIV to be considered successful, the following criteria should be met: reduction of the respiratory rate (f), increase in the tidal volume (Vt), improvement of the level of consciousness, reduction or cessation of the use of accessory muscles, increase in the partial pressure of oxygen (PaO_2_) and/or the peripheral oxygen saturation (SpO_2_), and reduction of PaCO_2_ without significant abdominal distension. When NIV is unsuccessful, orotracheal intubation (OTI) with initiation of invasive ventilation should immediately be performed. Successful NIV is expected in 75% of hypercapnia cases and approximately 50% of hypoxia cases.^(^ 5 ^)^

### Noninvasive mechanical ventilation in asthma exacerbations 

**Suggestion - **NIV may be used together with pharmacological treatment to improve airflow obstruction and reduce respiratory effort in individuals with moderate and severe asthma attacks.^(^ 5 ^,^ 7 ^)^

### Noninvasive mechanical ventilation in acute exacerbations of chronic obstructive pulmonary disease

**Recommendation - **NIV should be used in COPD exacerbations to reduce the need for intubation (relative risk - RR: 0.41 [95% confidence interval - 95%CI: 0.33-0.53]), reduce hospital length of stay and reduce mortality rates (RR: 0.52 [95%CI: 0.35-0.76).^(^ 5 ^,^ 6 ^)^

Acute cardiogenic pulmonary edema

**Recommendation - **NIV (bilevel positive airway pressure (BIPAP) with EPAP at 5 to 10 and IPAP at up to 15cmH_2_O) or CPAP at 5 to 10cmH_2_O must be used in individuals with acute cardiogenic pulmonary edema to reduce the need for endotracheal intubation (RR: 0.53 [95%CI: 0.34-0.83]), as well as the in-hospital mortality rate (RR: 0.6 [95%CI: 0.45-0.84]).^(^ 5 ^,^ 8 ^,^ 9 ^)^

### Noninvasive mechanical ventilation in acute respiratory distress syndrome

**Suggestion -** NIV may be used in ARDS, especially in cases of mild ARDS; the desired therapeutic goals should be achieved within thirty minutes to two hours. Avoid delaying intubation in unsuccessful cases.^(^ 5 ^,^ 10 ^)^

**Recommendation -** NIV should be avoided in severe ARDS due to the high rate of respiratory failure and need for OTI, especially when PaO_2_/FIO_2_<140 and the Simplified Acute Physiology Score (SAPS) II >35.^(^ 5 ^,^ 10 ^)^

### Noninvasive mechanical ventilation in severe community-acquired pneumonia

**Suggestion - **NIV may be used in severe cases of community-acquired pneumonia (CAP) with hypoxemic respiratory failure, particularly in individuals with concomitant COPD; the desired therapeutic effect should be achieved within thirty minutes to two hours. Avoid delaying intubation in unsuccessful cases.^(^ 5 ^,^ 11 ^)^

### Post-extubation 

**Recommendation -** NIV should be used to shorten the duration of invasive ventilation (NIV weaning-facilitating action), reduce mortality, reduce the rate of ventilator-associated pneumonia (VAP), and shorten the ICU and hospital stay of individuals with COPD and hypercapnia.^(^ 5 ^,^ 12 ^,^ 13 ^)^

**Chart 2:**
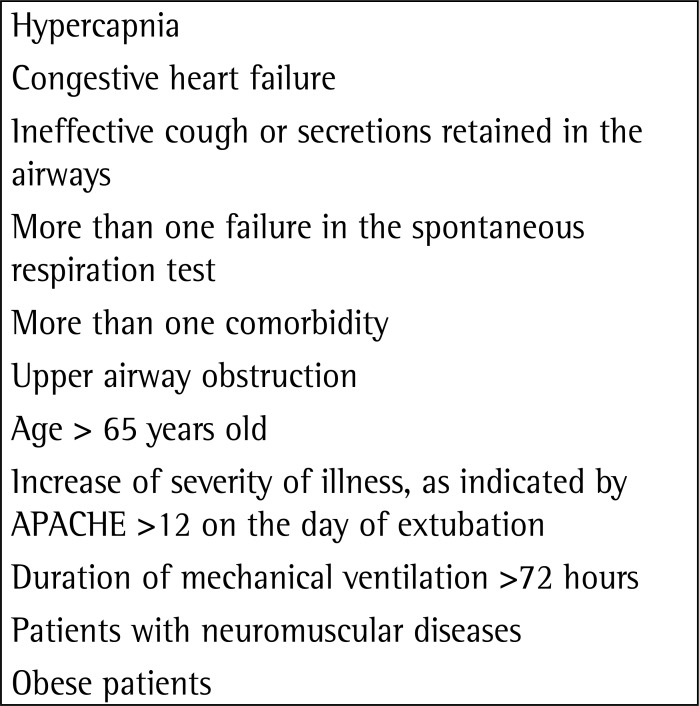
Patients considered to be at risk of extubation failure and who could benefit from noninvasive ventilation immediately after extubation (prophylactic use)

**Recommendation -** NIV should be started immediately in high-risk patients (Chart 2) to avoid ARF and reintubation (prophylactic action). ^(^ 5 ^,^ 12 ^-^ 15 ^)^

**Recommendation -** Avoid the use of NIV following the onset of a new respiratory failure event after extubation (curative action).^(^ 5 ^,^ 12 ^-^ 16 ^)^

Noninvasive ventilation in the postoperative period

**Recommendation - **NIV is indicated for the treatment of ARF that occurs in the immediate postoperative period following elective abdominal and thoracic surgery, and is associated with improvements in gas exchange, reductions in atelectasis, decreased work of breathing, and reduction in the need for OTI; furthermore, NIV may possibly reduce the mortality rate. In such cases, NIV must be used cautiously, with a full understanding of the limitations of and contraindications for its use.^(^ 5 ^,^ 16 ^-^ 19 ^)^

**Suggestion - **In esophageal surgery, NIV may be used to avoid ARF by maintaining lower inspiratory pressures (EPAP< 8 and IPAP < 15). This same suggestion applies to thoracic, abdominal, cardiac, or bariatric surgery.^(^ 5 ^,^ 17 ^-^ 19 ^)^

### Bronchoscopy

**Suggestion - **NIV may be used during and after bronchoscopy to reduce the risk of complications in individuals with severe refractory hypoxemia, postoperative respiratory failure, or severe COPD.^(^ 5 ^)^ Special care must be provided to individuals subjected to transbronchial biopsy, which includes maintenance of the airway pressures at <20cmH_2_O and performance of chest radiographs in cases of clinical decompensation and approximately six hours after the procedure (in order to rule out pneumothorax). 

## Masks and ventilators for providing noninvasive ventilation

Ventilators available in Brazil: characteristics, advantages and disadvantages

**Chart 3:**
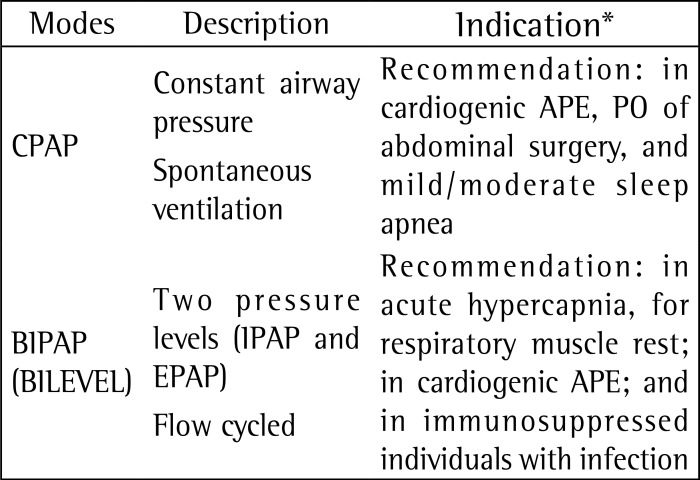
Types of modes of ventilation for noninvasive support CPAP - continuous positive airway pressure; BIPAP - bilevel positive airway pressure; APE - acute pulmonary edema; PO - postoperative period; IPAP - inspiratory positive airway pressure; EPAP - expiratory positive airway pressure. *except when contraindicated.

**Suggestion - **NIV may be performed using portable ventilators specifically designed for this purpose and that have leak compensation. The device should be coupled to a nasal/facial interface with a single-limb circuit and a built-in exhalation port. NIV may also be performed using microprocessor-controlled ventilators with software for this specific purpose, which should be coupled to the nasal/facial interface by means of an elbow connector and the ventilator's dual-limb circuit (Chart 1 - electronic supplementary material ). The CPAP mode may be generated using of flow generators^(20,21) ^(Chart 3).

### Carbon dioxide rebreathing

**Suggestion -** Avoid or minimize CO_2_ rebreathing when single-limb circuit ventilators are used. The risk of CO_2_ rebreathing is lower with systems where the exhalation ports are built into the mask compared to ones where the exhalation ports are in the ventilator circuit. Other factors that might contribute to CO_2_ rebreathing are use of low PEEP and reduced pressure support; special attention is needed in such cases.^(^ 22 ^)^

### Oxygen supplementation

**Suggestion -** In the case of ventilators with a gas blender, the device allow adjustments in the oxygen (O_2_) supplementation. When portable NIV devices without a gas blender are used, oxygen should be given straight to the mask beyond the exhalation port using an external O_2_ source. The supplemental FiO_2 _depends on the O_2 _flow, position of the O_2 _connector in the circuit, degree of leak in the ventilator circuit, the type of interface used, and the level of IPAP and EPAP supplied.^(^ 23 ^-^ 26 ^)^

### Monitoring during noninvasive ventilation

**Recommendation -** Monitor Vt, f and SpO_2_ during the use of NIV. Use a graphical monitoring system when available. Asynchrony, air leaks, auto-PEEP, efficacy of effort, and the leak compensation mechanism should be continuously monitored. ^(^ 26 ^,^ 27 ^)^

Indications for the choice of interface in common clinical situations 

**Recommendation -** Choose an appropriate interface, i.e., the one that adjusts best to the patient's face to achieve the greatest clinical efficiency. 

**Recommendation - **Use interfaces without nasal compression when the estimated duration of NIV is >24 to 48 hours. 

**Recommendation** - Use interfaces with a PEEP valve when CPAP with flow generator is used. 

**Chart 4:**
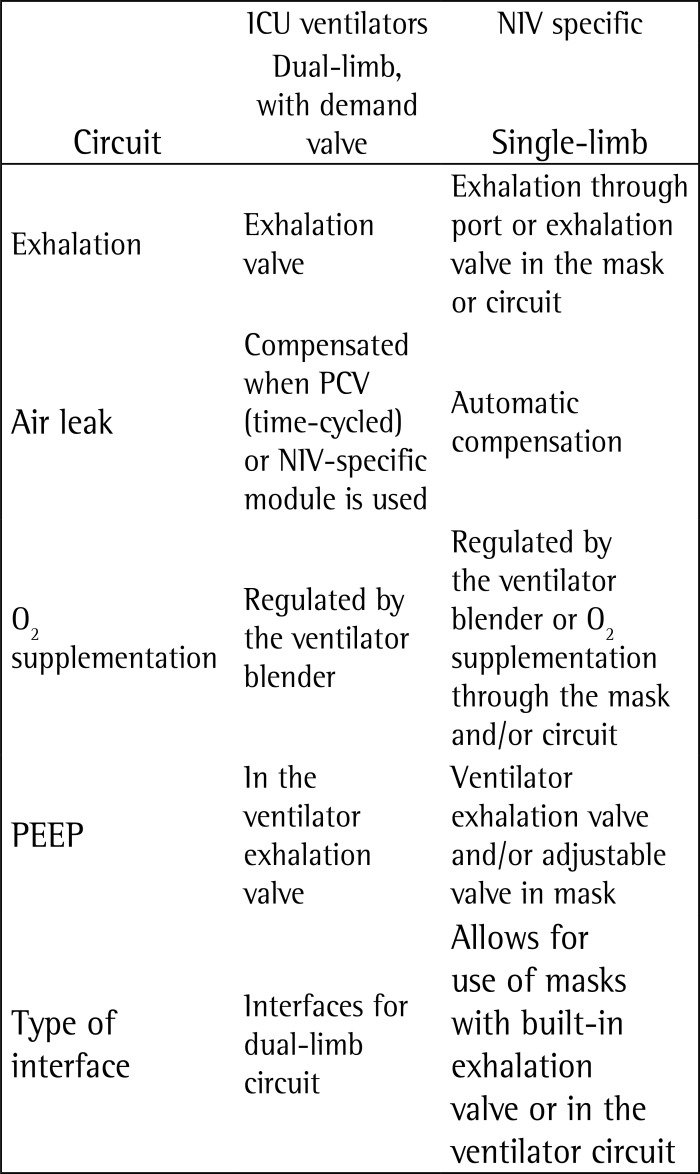
Differences between noninvasive ventilation using portable ventilators specific for noninvasive ventilation and intensive care unit microprocessorcontrolled ventilators with a non-invasive ventilation module ICU - intensive care unit; PCV - pressure-controlled ventilation; NIV - noninvasive ventilation; O2 - oxygen; PEEP - positive end-expiratory pressure

**Recommendation -** When NIV is performed with an ICU (conventional microprocessor-controlled) ventilator, use a mask connected to a dual-limb circuit. When NIV-specific ventilators are used, use a mask for single-limb circuits^(^ 20 ^,^ 23 ^-^ 25 ^)^ (Chart 4).

### Adaptation to and tolerance of interfaces

Nasal masks 

**Suggestion -** Nasal masks may be used in cases of mild ARF for patients with claustrophobia or maladaptation to the facial mask.

**Chart 5:**
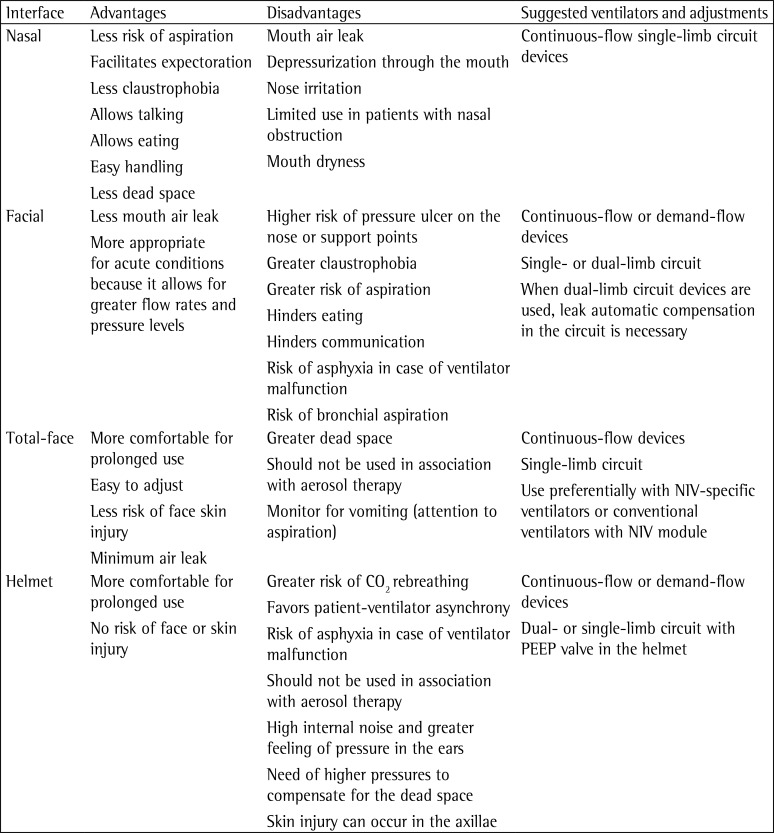
Advantages and disadvantages of the various types of interfaces NIV - noninvasive ventilation; CO2 - carbon dioxide; PEEP - positive end-expiratory pressure.

**Suggestion -** Several interfaces can be combined when patients need continuous ventilatory support to avoid the occurrence of ischemia due to reduction of blood flow that is caused by the pressure of the mask on the patient's face (Chart 5).^(^ 25 ^)^

Oral-nasal (facial) masks

**Recommendation - **Use face masks in cases of mild to moderate ARF to achieve fast improvement of physiological parameters (gas exchange and work of breathing). Monitor the patient's tolerance and the occurrence of side effects, such as ulcers at support points and gastric distension. 

Full-face mask and Helmet

**Recommendation -** Use these interfaces in the most severe cases of hypoxemic respiratory failure because they allow for greater airway pressurization. As those devices cover the patient's entire face, the pressure they exert on the skin is more widely distributed, and thus pressure points on the nose are minimized, consequently reducing the risk of skin injury (Chart 5). 

**Suggestion -** Helmet-like masks can be used, when available, in less severe cases of respiratory failure. This type of mask is hermetically sealed around the patient's neck by an air cushion that is inflated by the ventilator itself, and the points of contact are on the neck, shoulders and axillary region. However, as the dead space is large, the use of helmet-like masks in individuals with ventilatory disorders is limited; such patients may need requires correction by means of higher levels of pressure support. Internal noise is another cause of discomfort that should be taken into consideration. This type of interface may induce trigger asynchrony due to delayed release of the inspiratory flow, with a consequent increase in the work of breathing^(28-30) ^(Chart 5).

## Intubation and tracheostomy

Techniques for elective, semi-elective and emergency intubation

**Recommendation -** Use direct laryngoscopy with visualization of the larynx as the fastest and most reliable method for insertion of the orotracheal tube in elective or emergency cases. Three unsuccessful attempts at intubation by an experienced physician are considered to characterize a difficult airway, in which case the corresponding specific guidelines should be followed.^(^ 31 ^,^ 32 ^)^

Elective intubation 

**Suggestion -** Elective tracheal intubation is an intubation that is performed when there are no signs of imminent failure of airway protection, ventilation, and/or oxygenation. Under such conditions, the method of tracheal intubation that is most suited to each individual patient should be selected. Use direct laryngoscopy with OTI as the first-choice method.^(^ 31 ^,^ 32 ^)^

**Suggestion -** Adequately prepare the patient for tracheal intubation, including pre-oxygenation, monitoring, and appropriate positioning during the procedure in order to achieve optimal laryngoscopy.^(^ 32 ^,^ 33 ^)^

**Suggestion -** A curved-blade laryngoscope of the appropriate size is preferred. A straight-blade laryngoscope may be used to achieve appropriate larynx exposure in cases where intubation is difficult.^(^ 31 ^,^ 32 ^,^ 34 ^)^

Emergency intubation 

**Suggestion -** Use the rapid sequence intubation technique to avoid the risk of gastric aspiration. Insert the orotracheal tube as soon as possible after loss of consciousness occurs. ^(^ 32 ^,^ 35 ^,^ 36 ^)^

**Suggestion -** Use hypnotics (propofol, etomidate, ketamine or thiopental), opioids (fentanyl, alfentanil or remifentanil) and neuromuscular blocking drugs (rocuronium or succinylcholine). The Sellick maneuver (cricoid pressure) can be performed during the procedure to minimize the risk of gastric aspiration.^(^ 32 ^,^ 35 ^-^ 37 ^)^

Techniques and indications for tracheostomy: advantages and disadvantages

Timing of tracheostomy: recommendations based on the cause of respiratory failure

Spinal cord injury

**Suggestion -** Perform tracheostomy early (within seven days). High cervical spinal cord injury (C5 or above) is an independent predictor of the need for prolonged MV. Patients with injuries at lower levels should be assessed on an individual basis.^(^ 32 ^,^ 38 ^)^

Traumatic brain injury

**Suggestion - **Perform tracheostomy early (within seven days) in the most severe cases (Glasgow Coma Scale <8), as patients with traumatic brain injury usually require prolonged ventilatory support. The evidence regarding reductions in the VAP rate is contradictory, and there is no evidence that early tracheostomy reduces mortality, airway injury, or the length of hospital stay.^(^ 32 ^,^ 38 ^,^ 39 ^)^

Patients with trauma not affecting the central nervous system

**Suggestion -** Early tracheostomy is indicated when prolonged ventilatory support is anticipated. ^(^ 32 ^,^ 38 ^-^ 40 ^)^

Patients admitted to the intensive care unit for clinical causes

**Recommendation -** Wait 14 days to perform a tracheostomy, as early use of this procedure does not reduce the 30-day mortality rate, length of stay in the ICU, or the need for sedation.^(^ 32 ^,^ 41 ^-^ 44 ^)^

### Tracheostomy techniques

**Recommendation** - Perform percutaneous or conventional tracheostomy, depending on the available resources and the staff's experience. Percutaneous tracheostomy can be performed at the bedside by ICU staff. Although it is more expensive and demands that a bronchoscopy be performed to increase its safety, the associated rates of surgical wound infection are lower. Conventional tracheostomy must be performed in an operating room by specialized staff, except for the case of ICUs that are equipped with a room for surgical procedures. Both techniques have similar rates of major complications, such as bleeding, subcutaneous emphysema, pneumothorax and death.^(^ 32 ^,^ 45 ^-^ 47 ^)^

Initial adjustment of invasive ventilation and conventional ventilation modes

### Ventilation adjustment

**Recommendation -** Use the FIO_2_ needed to maintain SpO_2_ at 93 - 97%.^(^ 48 ^,^ 49 ^)^

**Recommendation -** Use a Vt of 6mL/kg/ predicted body weight. Reassess as a function of changes in the patient's clinical condition. ^(^ 48 ^-^ 52 ^)^

**Recommendation - **Use the assist-control mode (AC) as either volume-cycled (VCV) or time-cycled pressure-limited, known as pressure controlled ventilation mode (PCV), and reassess within the first few hours based on the patient's clinical condition.^(^ 48 ^-^ 51 ^)^

**Recommendation -** Adjust the initial f = 12 to 16 breaths per minute, with an inspiratory flow rate or inspiratory time required to maintain the inspiration to expiration ratio (I:E) initially at 1:2 or 1:3. In patients with obstructive disease, the initial f can be lower (<12 breaths per minute), and in patients with restrictive disease it may be higher (e.g., >20 breaths per minute, if required by the patient's clinical condition). Reassess as soon as the first arterial blood gas results are available.^(^ 48 ^,^ 51 ^-^ 54 ^)^

**Recommendation -** Establish the type of ventilator triggering. The more widely available types of ventilator triggering are the time-triggered (ventilator-controlled mode) and the patient-triggered (flow or pressure triggered, also known as pneumatically triggered) modes. The ventilator's sensitivity should be adjusted to the most sensitive level to avoid auto-triggering. The ventilator can also be triggered by neural stimuli (neurally adjusted ventilatory assist-NAVA).^(^ 48 ^,^ 51 ^-^ 54 ^)^

**Recommendation -** Initially use a PEEP of 3 to 5cmH_2_O, except in cases of diseases such as ARDS, where the PEEP value should be assessed according to the specific guidelines described in the each topic of the present recommendations. ^(^ 48 ^,^ 49 ^,^ 55 ^-^ 57 ^)^

**Recommendation -** Use passive heaters and humidifiers in individuals undergoing MV. When available, active humidification and heating should be performed in patients with thick secretions, and optimal humidification should be maintained to avoid obstruction of the orotracheal tube.^(^ 58 ^)^

**Recommendation -** Set the alarms on an individual basis, using specificity and sensitivity parameters appropriate for the patient's clinical condition. Also, an apnea backup and the specific parameters for apnea should be adjusted if they are available in the device. 

**Recommendation -** After the initial parameters are defined, check the Vt, pressure and flow curves to establish whether their values correspond to the expected parameters or if immediate readjustment is needed. Check pulse oximetry, which should be continuously monitored. Initially, set the maximum airway pressure at 40 cmH_2_O to avoid barotrauma, and adjust as soon as possible based on the patient's clinical condition.^(^ 48 ^,^ 51 ^-^ 54 ^)^

**Recommendation - **Arterial blood gases must be assessed after 30 minutes of steady ventilation to check whether the ventilation and gas exchange goals were met. If they were not, perform necessary adjustments of the mode and cycling parameters.^(^ 48 ^-^ 51 ^)^

**Recommendation -** Assess the eventual hemodynamic repercussions of MV. Investigate the presence of hypovolemia, auto-PEEP and/or pneumothorax in patients with hypotension that is associated with positive pressure ventilation. 

**Recommendation -** Maintain the most appropriate level of muscle work. In patients with high inspiratory flow demands, use opioids to reduce the ventilatory drive and provide appropriate comfort for the patient. Induce muscle rest for 24 to 48 hours in patients with respiratory muscle fatigue or hemodynamic instability. 

**Recommendation -** In patients who do not need muscle rest, start an assist mode of ventilation as soon as possible, with appropriate adjustment of the ventilator's sensitivity. Avoid ventilator-induced diaphragmatic dysfunction, which usually occurs after 18 hours of controlled ventilation. 

**Suggestion -** In older adults, patients who require prolonged use of controlled modes of ventilation, malnourished patients, patients using corticosteroids or neuromuscular blocking agents, and individuals with hypothyroidism, pay special attention to the assessment of respiratory muscle function.

### Conventional modes of ventilation(59)

**Suggestion -** Use the volume assist-control mode (VCV) when the aim is to maintain a more stable minute volume (Vt x f). This mode of ventilation can be timed (controlled), and pressure- and flow-triggered (assisted) and is cycled off when the preset inspired Vt is achieved. The airway pressure is variable and depends on the patient's ventilatory mechanics (special attention should be paid to monitoring the peak and plateau pressures when this mode is used, and it should be ensured that the maximum airway pressure alarm is properly set). This mode is also used to measure the peak and plateau pressures for calculating the compliance and resistance of the respiratory system under a constant square-wave inspiratory flow pattern (see this specific topic in the present recommendations). 

**Suggestion -** Use the PCV assist-control mode when respiratory mechanics are impaired (low compliance and/or high resistance), as it allows for better control of the airway and alveolar pressures. This mode characteristically limits pressure throughout all the inspiratory phase and is time-cycled. The inspiratory time is set in seconds by the caregiver. The flow is free and decelerating waveform. In this mode, the Vt is variable and depends on the administered delta pressure and the patient's ventilatory mechanics (special attention should be paid to monitoring the expired Vt and adjusting the maximum and minimum minute volume alarms). The inspiratory flow speed (ramp, rise time or slope) can be increased or reduced. The rise time can be faster in patients with obstructive disease to obtain a better Vt. Special attention should be paid to the possible occurrence of peak flow overshoot*.* In patients with restrictive disease, a slower rise time should be used. 

**Suggestion -** PSV is considered the preferential mode during assisted/spontaneous ventilation. It should be started as soon as possible, based on the patient's clinical condition. This is an exclusively patient-triggered mode, and can be flow- or pressure-triggered. Characteristically, pressure is limited throughout all the inspiratory phase and is cycled off when the inspiratory flow falls, typically to 25% of the peak inspiratory flow. This cycling criterion (% of the peak inspiratory flow) can be set between 5% and 80% in some of the most modern ventilators, which allows a reduction of the inspiratory time in patients with obstructive disease (% of the cycling off >25%) and an increase in the inspiratory time in patients with restrictive disease (% of the cycling off <25%). The rise time can be faster in patients with obstructive disease, thus decreasing inspiratory time and obtaining a better Vt. Special attention should be paid to the occurrence of peak flow overshoot. In patients with restrictive disease, use a slower rise time, which may be accompanied by a Vt gain. 

**Suggestion -** Use pressure-cycled ventilators just if they are the only ventilators available. The ventilator can be time and pressure triggered. Characteristically, it provides a fixed flow rate until the airway pressure reaches the value predetermined by the caregiver (cycling). As a result, the Vt is unknown, and consequently the use of an external ventilometer (Wright's ventilometer) is recommended; alternatively, arterial blood gases can be assessed after 20 minutes of steady ventilation to check whether the PaCO_2 _is compatible with the patient's clinical condition (35 to 45 mmHg in most cases). This device usually does not have a built-in O_2_ blender or alarms. The multi-disciplinary staff must pay special attention to monitoring both ventilation and oxygenation. 

**Recommendation - **Avoid the use of Synchronized Intermittent Mandatory Ventilation (SIMV) because it has been shown to be associated with a delay in MV weaning. Currently, the use of SIMV is restricted to patients in whom minimal minute volume is necessary at the beginning of MV weaning process (e.g., individuals with neuropathy, or upon immediate awakening from general anesthesia). As soon as the ventilatory drive stabilizes, SIMV should be shifted to PSV. A brief description of SIMV mode follows. Controlled cycles can be volume-cycled (V-SIMV) or pressure-limited (P-SIMV). Spontaneous cycles should be associated with PSV. SIMV is characterized by the fact that it allows for controlled, assisted and spontaneous cycles to occur within the same time window (TW), which is determined by the f of the controlled mode. Controlled cycles only occur when a patient assisted trigger did not occur in the immediately preceding TW. Otherwise, the ventilator waits for the next patient-trigger, i.e., an assisted cycle. Spontaneous cycles supported by PSV can occur in the remainder of the TW. 

## Asynchrony and new modes of mechanical ventilation

### Patient-ventilator asynchrony

**Comment -** Patient-ventilator asynchrony is a lack of coordination between the patient's inspiratory effort and ventilatory needs and the support provided by the ventilator.^(^ 60 ^)^ Asynchrony is a frequent event, occurring in 10% to 80% of all ventilator cycles, and is associated with prolonged of MV and ICU stays.^(^ 61 ^)^

**Recommendation - **The presence of asynchrony should be actively assessed during the assessment of patients subjected to MV, and it should be corrected.

### Trigger asynchrony 

Ineffective triggering

**Comment -** Ineffective triggering occurs when the patient's inspiratory effort is not enough to trigger the ventilator.^(^ 62 ^)^ The reason might be a maladjustment in ventilator sensitivity or patient-related factors such as respiratory muscle weakness, central respiratory depression, dynamic hyperinflation (auto-PEEP), or longer mechanical inspiratory time relative to the neurally stimulated inspiratory time.^(62,63)^

**Figure 1:**
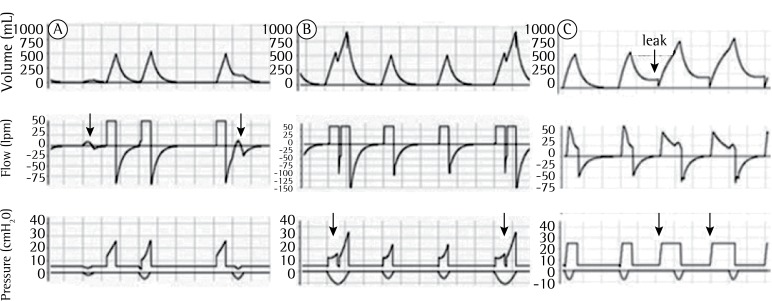
Trigger asynchronies identified in volume-, flow- and pressure-time curves, indicated by arrows. Negative deflections in the pressure-time curves represent the patient's inspiratory effort (muscle pressure), which are only visible when the esophageal pressure is monitored. Panel A) Lost efforts. The first arrow indicates a weak stimulus, which is unable to trigger the ventilator, thus resulting in a small positive flow wave and minimal tidal volume. The second arrow points to effort during expiration, which failed to trigger the ventilator and merely sufficed for the flow to return to baseline and become slightly positive. Panel B) Double-triggering. Example in volume-controlled ventilation. The patient's inspiratory efforts persist at the time of cycling-off, thus triggering another cycle. The corresponding volumes are added together (stacking), and the airway pressure increases, causing the high-pressure alarm to go off. Panel C) Auto-triggering. In the support pressure mode, some cycles are triggered without a patient inspiratory effort, which can be facilitated by leaks; this is observed in the volume-time curve, which does not return to baseline (the inspired volume is greater than the expired volume). Figures obtained at Xlung.net, a virtual mechanical ventilation simulator. Available at: http//:www.xlung.net.

**Identification **- Clinical examination of the patient's chest and abdomen can reveal that the inspiratory effort is not accompanied by a ventilator cycle.^(64,65)^ Figure 1A shows how to identify this asynchrony in ventilator curves.^(^ 64 ^,^ 65 ^)^

**Recommendation -** To correct trigger asynchrony, the ventilator's sensitivity should be adjusted to the most sensitive level possible, while avoiding auto-triggering; in addition, pressure triggering can be shifted to flow triggering (which is usually more sensitive). 

**Suggestion -** In the presence of auto-PEEP, extrinsic PEEP may be titrated up to 70 to 85% of the auto-PEEP; the effects of this adjustment on asynchrony must be checked.^(^ 62 ^)^ During PSV, one might attempt to reduce the pressure that is administered or to increase the percentage of the cycling criterion.^(^ 63 ^)^ When pressure-controlled ventilation (PCV) is used, one might attempt to reduce the inspiratory time, or in cases where VCV is used, to increase the inspiratory flow rate or reduce the pause time.^(^ 62 ^,^ 63 ^)^

Double triggering

**Comment - **Two consecutive cycles are triggered by a single patient inspiratory effort. The ventilator's mechanical inspiratory time is shorter than the patient's neural inspiratory time. ^(3)^

**Identification** - Clinically two consecutive cycles without an interval between them can be observed; this pattern that may be repeated quite often. Figure 1B shows how to identify this asynchrony in the ventilator curves.^(^ 64 ^-^ 66 ^)^

**Suggestion -** In VCV, the inspiratory flow rate and/or the Vt should be increased, while still complying with the safety thresholds. Alternatively, VCV could be shifted to PCV or PSV, in which the inspiratory flow rate varies as a function of the patient's inspiratory effort. When double triggering occurs under PCV, the inspiratory time and/or delta of pression value could be increased. In PSV, one might try to increase the pressure level or reduce the percentage of the cycling criterion. ^(62,63)^

Auto-triggering 

**Comment -** The ventilator is triggered in the absence of a patient's inspiratory effort. This can be caused by overly high ventilatory sensitivity, leaks in the system, flow alterations due to presence of condensates in the circuit, detection of the heartbeat, or wide variations in chest pressure that are due to stroke volume (Figure 1C).^(60,62)^

**Identification - **The observed respiratory frequency is higher than the adjusted one, and the cycles are not preceded by indicators of patient inspiratory effort.^(^ 64 ^-^ 67 ^)^

**Recommendation -** Once the presence of leak or condensate in the circuit is corrected or ruled out, gradually reduce the ventilator's sensitivity to a level sufficient for auto-triggering to stop. ^(^ 62 ^,^ 64 ^-^ 66 ^)^

Flow asynchrony

Insufficient inspiratory flow

**Comment -** In insufficient inspiratory flow, the flow offered is lower than patient ventilatory demands. This typically occurs when the flow is set by the operator and cannot be increased by the patient's inspiratory effort, as in VCV. Nevertheless, this phenomenon might also occur in PCV and PSV, when the adjusted pressure is insufficient to ensure an appropriate balance between the patient's ventilatory demands and mechanics.^(67,68)^

**Figure 2:**
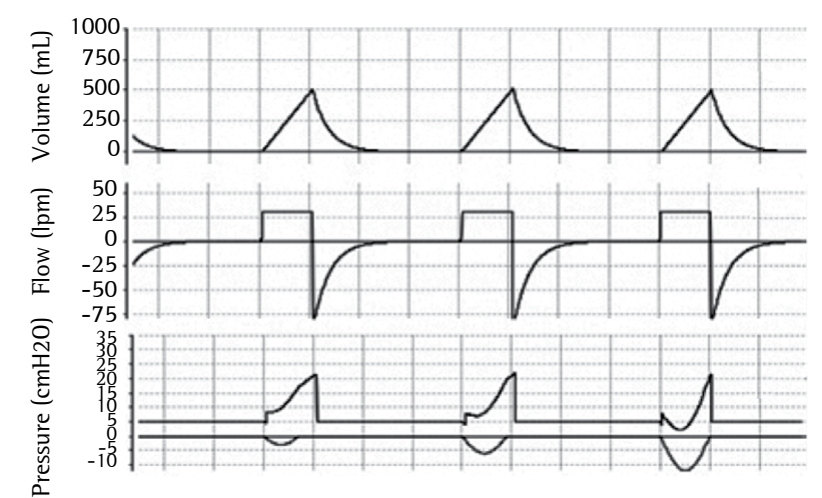
Flow asynchrony. In volume-controlled mode, the flow rate was adjusted below the patient's demand; the patient thus maintained muscle effort throughout inspiration, and the curve consequently became concave and upward. The asynchrony exhibits increasing intensity from the first to the third cycle, as represented in the figure. The negative deflections in the pressure-time curve represent the patient's inspiratory effort (muscle pressure) and are only visible when esophageal pressure is monitored. Figures obtained at Xlung.net, a virtual mechanical ventilation simulator. Available at: http//:www.xlung.net.

**Identification **- The patient exhibits discomfort and uses the accessory respiratory muscles. Figure 2 shows how to identify this asynchrony in the ventilator curves.^(^ 67 ^,^ 68 ^)^

**Recommendation -** Correct the causes of the increased ventilatory demands, such as fever, pain, anxiety, or acidosis. In VCV, increase the inspiratory flow rate and check for signs of patient comfort, as well as the shape of the pressure - time curve; shift to PCV or PSV, in which the flow is not fixed;^(^ 68 ^)^ adjust the speed necessary to achieve the maximum airway pressure (rise time - speed of flow rise, or increasing the controlled pressure value).^(^ 69 ^)^

Excessive inspiratory flow

**Comment -** Excessive inspiratory flow can occur in VCV when the flow is set above the level desired by the patient, or in PCV or PSV when high pressures or a faster rise time are set. 

**Identification** - In VCV, the pressure - time curve peak is achieved too early.^(68,69) ^In PCV or PSV, the airway pressure becomes higher than the adjusted level, a phenomenon known as overshoot.^(^ 69 ^)^

**Recommendation -** In VCV, reduce the flow rate; in PCV and PSV, the rise time should be reduced until the overshoot disappears.^(^ 68 ^)^

Cycling asynchrony

Premature cycling

**Figure 3:**
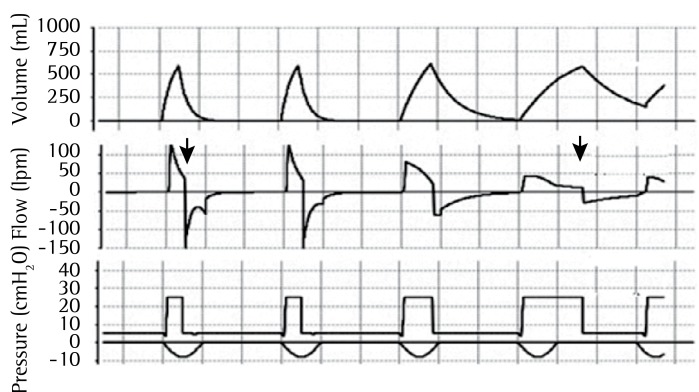
Cycling asynchronies during pressure support ventilation. In the first cycle, the cutoff point of 25% of the peak inspiratory flow (percentage of the cycling criterion) was reached rapidly; the ventilator's inspiratory time was therefore shorter than the time desired by the patient. This is shown in the expiratory segment of the flow curve, which tends to return to the baseline as a result of the patient's inspiratory effort, which is still present. The last cycle represents the opposite situation, i.e., delayed cycling. The flow reduction occurs very slowly, which is typical of airway obstruction; the cycling threshold is therefore reached with some delay. Sometimes, the cycle is interrupted by a contraction of the respiratory muscles, which causes an increase above the support pressure adjusted at the end of inspiration (not shown in this figure). Figures obtained at Xlung.net, a virtual mechanical ventilation simulator. Available at: http//:www.xlung.net

**Comment -** In premature cycling, the ventilator interrupts the inspiratory flow before the patient desired; in other words, the ventilator's mechanical inspiratory time is shorter than the patient's neurally controlled inspiratory time.^(^ 70 ^)^ In VCV and PCV, the inspiratory time is adjusted by the operator. In PSV, premature cycling occurs when a low pressure level and/or a high percentage of the cycling criterion are adjusted.^(^ 70 ^)^ Figure 3 shows how to identify this asynchrony in the ventilator curves. In some cases, the patient's inspiratory effort may suffice to trigger a new cycle (double cycling).^(64,66,70)^

**Recommendation -** In VCV, the inspiratory flow rate may be reduced and/or Vt may be increased in compliance with the safety thresholds. Alternatively, one might shift to PCV or PSV, where the inspiratory flow rate varies as a function of the patient's inspiratory effort. When premature cycling occurs in PCV, the inspiratory time and/or the delta of inspiratory pressure value may be increased. In PSV, one could try to increase the pressure level or reduce the percentage of the cycling criterion.^(^ 62 ^,^ 63 ^,^ 70 ^)^

Delayed cycling

**Comment -** In delayed cycling, the ventilator's mechanical inspiratory time is longer than the time desired by the patient; in other words, the ventilator cycling time is longer than the patient's neurally controlled inspiratory time. In VCV, this can occur when the inspiratory time is extended by setting a high Vt or a low inspiratory flow rate or if inadequate use is made of the inspiratory pause. In PCV, delayed cycling occurs when the inspiratory time is set beyond the time desired by the patient. In PSV, particularly in the case of obstructive diseases such as COPD, the increase in the resistance and compliance of the respiratory system gradually slows down the inspiratory flow rate, thus increasing the inspiratory time.^(70)^ Figure 3 shows how to identify this asynchrony in the ventilator curves.^(^ 64 ^,^ 66 ^)^

**Recommendation -** In modes of ventilation in which the operator adjusts the inspiratory time, the latter should be reduced. In PSV, the percentage of the cycling criterion might be increased (e.g., from 25% to 40% or even higher). ^(70)^

**Suggestion** - Patient-ventilator asynchrony should be treated by adjusting the ventilation parameters or shifting to other modes of ventilation (experts' opinion). 

### Advanced modes of mechanical ventilation

**Comment -** The choice of the mode of ventilation should be based on the severity of the patient's condition.^(71) ^In patients with respiratory failure and asynchrony, a shift to another mode of ventilation may be an option. The number and complexity of modes of ventilation exhibited a significant rise in recent years. Despite their increasing availability, the clinical impact of these newer modes of ventilation has not yet been thoroughly investigated.^(^ 71 ^)^

**Suggestion** - Use advanced modes of ventilation in specific clinical situations, provided that the operator is thoroughly acquainted with the parameters of each mode and that the patient's clinical condition can benefit from the resources specific to each mode. 

### Pressure-regulated volume-control mode

**Comment -** This is a time-cycled pressure-limited ventilation mode. The ventilator readjusts the pressure limit at each cycle based on the Vt obtained in the previous one, until reaching a target Vt that has been preset by the operator. ^(72)^

**Suggestion -** Indicate when limited-pressure Vt control is desired, aiming to automatically adjust the inspiratory pressure if the respiratory mechanics change. 

**Recommendation - **Caution is required in adjusting the target Vt, as undesirable increases of the inspiratory pressure may result. 

Airway pressure release ventilation and bilevel positive airway pressure ventilation

**Comment -** Airway pressure release ventilation (APRV) is pressure-limited and time-cycled, and is considered to be a spontaneous mode of ventilation. The operator adjusts the pressure high (PEEPhigh) and low (PEEPlow), the PEEPhigh to PEEPlow ratio, and the frequency of alternation between both PEEP levels; the time of PEEPhigh must be longer than the time of PEEPlow. The BIPAP mode also uses two PEEP levels, but the time of PEEPlow is longer than that of PEEPhigh. The patient can breathe spontaneously at both pressure levels.^(73,74) ^Support pressure may also be applied, as its value is added to the PEEPlow value, and the final airway pressure (Paw) is the result of the sum of PSV + PEEPlow. When the PEEPhigh value is lower than PSV + PEEPlow value, during the PEEPhigh period the ventilator only complements the PSV value to reach the same level of Paw as in PEEPlow + PSV.

**Suggestion -** Use APRV when maintenance of spontaneous ventilation and alveolar recruitment is necessary; APRV may improve gas exchange and reduce dead space and asynchrony. 

**Recommendation - **Caution is required when regulating the alternation between the two pressure levels because in this mode, the minute volume results from the sum of the obtained Vt, when the pressures are alternated, plus the Vt generated from PSV cicles. 

### Proportional assist ventilation 

**Comment -** Proportional assist ventilation (PAV) is a spontaneous ventilation mode that follows the equation of motion to generate inspiration pressure (Pvent) in proportion to the patient's inspiratory effort (Pmus). Therefore, when the Pmus decreases, Pvent also decreases, and vice-versa.^(71,75-79) ^Some studies found better patient-ventilator synchrony when PAV, or its latest version, PAV plus (PAV+), is used compared to PSV. The PAV+ software estimates the work of breathing (WOB) of both patient and mechanical ventilator using the equation of motion, and calculates compliance and resistance through the application of 300-ms inspiratory micro-pauses every 4 to 10 ventilation cycles. 

**Indication** - PAV is indicated for patients with respiratory drive and significant asynchrony under spontaneous modes of ventilation, PSV in particular. It is also indicated when one wants to determine the patient's WOB and mechanical measurements during assisted ventilation, e.g., for obtaining real-time intrinsic PEEP estimates.^(^ 75 ^-^ 79 ^)^

**Recommendation - **Before starting the PAV+ mode, the operator should set the type and diameter of the tracheal prosthesis, the type of humidifier, maximum Vt and maximum allowed airway pressure (limits) in the ventilator. 

**Recommendation -** Set the percentage of initial support at 50% to achieve a patient WOB of 0.3 - 0.7 J/L with adequate Vt and f. Pvent increases proportionally with the patient's Pmus. The support percentage should not exceed 90%. If a greater percentage is needed, conventional assisted-controlled ventilation modes are recommended. Gradually reduce the support percentage in parallel with improvement of the patient's clinical condition, to as low as 30%. When the (abovementioned) parameters are maintained, consider to extubate the patient. 

**Suggestion -** PAV is an alternative to PSV in patients with significant asynchrony; it has the potential to improve the patient-ventilator interaction. 

**Recommendation -** PAV should be avoided in patients without respiratory drive, as well as in MV with leaks that impair the measurements of resistance and compliance. 

### Automatic tube compensation

**Comment -** Automatic tube compensation (ATC) is a spontaneous mode of ventilation that aims at reducing the resistive work imposed by the presence of an artificial airway - i.e., an orotracheal or tracheostomy tube. Some studies showed reductions in the work of breathing and better patient comfort with ATC compared to PSV.^(80-82)^

**Suggestion -** Use ATC plus or minus PSV to automatically compensate for the increase in the resistive work associated with the presence of a tracheal prosthesis (in PSV, the compensation should be calculated by the caregiver as a function of the prosthesis diameter; the smaller the diameter, the higher the PSV value should be, e.g., PSV=5cmH_2_O for 9-mm tubes, and PSV=9cmH_2_O for 6-mm tubes). 

**Recommendation -** ATC is contraindicated for patients without respiratory drive, and care should be taken in patients who have excess secretions that interfere with inspiratory flow; the airway pressure alarms should be properly set. 

### Neurally adjusted ventilatory assist

**Comment -** Neurally adjusted ventilatory assist (NAVA) is a mode of ventilation that captures the electrical activity of the diaphragm and uses it as a criterion for triggering and cycling-off of the ventilator, thus providing inspiratory support in proportion to the electrical activity of the diaphragm. Use of NAVA requires placement of an esophageal-gastric catheter, with sensors positioned on the distal third of the esophagus to detect the electrical activity of the diaphragm. ^(5,6) ^In clinical studies, use of NAVA was associated with improved patient-ventilator synchrony when compared to PSV. 

**Indications** - NAVA is indicated for patients with respiratory drive and significant asynchrony on spontaneous ventilation, and particularly in the case of loss of effort with PSV, as in patients with auto-PEEP (intrinsic PEEP).^(^ 77 ^-^ 79 ^,^ 83 ^)^

**Recommendation -** Special care is required in patients with oronasal or esophageal disorders that might hinder the passage or proper positioning of the NAVA catheter. The NAVA catheter should be properly placed and fixed, and its position should be checked on a regular basis. Once the probe is fixed, measure the electrical activity of the diaphragm (Edi), and adjust the NAVA gain as a function of the Vt, f and airway pressure (Edi versus NAVA gain). The ventilator is triggered by 0.5-µV variations in the Edi. From that point onwards, the ventilator delivers free flow as a function of the Edi reading. The maximum airway pressure results from adding [maximum Edi - minimum Edi] multiplied by the NAVA gain to the extrinsic PEEP value. Cycling-off occurs when Edi falls to 70% of the maximum Edi peak detected.^(^ 77 ^-^ 79 ^,^ 83 ^)^

**Recommendation - **NAVA gain is adjusted as a function of the patient's clinical condition, and should be assessed on an individual basis.

**Suggestion -** NAVA may be an alternative to PSV for patients with significant asynchrony; it may improve the patient-ventilator interaction, especially in cases where there is loss of respiratory effort. 

### Adaptive support ventilation

**Comment -** Adaptive support ventilation (ASV) employs an algorithm to select the Vt and f combination necessary to reach the minute volume set by the caregiver by means of spontaneous and controlled cycles, with the lowest possible airway pressure. The version known as Intellivent-ASV employs an end-tidal CO_2_ (ETCO_2_) and a SpO_2_ sensor to adjust the PEEP and FIO_2 _automatically by means of a table.^(^ 83 ^)^

**Indications** - ASV is indicated for patients with severe respiratory failure when reductions of the work of breathing and stimulation of spontaneous respiration are desired.

**Suggestion -** Use ASV to ensure minute volume with appropriate lung protection in patients with unstable ventilatory drive, asynchrony or discomfort. Monitor for possible occurrence of leaks or excess secretions, which may impair the appropriate functioning of the ventilator. 

## Ventilators for invasive ventilation

### Choice of mechanical ventilator

The following questions should be answered when choosing mechanical ventilators: in which patient population they will be used (adults, children, or newborn infants)? How often are patients with severe ventilation problems admitted (e.g., ARDS, severe obstructive disease, pulmonary fistula, etc.)? What information do ventilators provide to contribute to decision-making about ventilatory support in that particular ICU? How will patients be weaned from MV? What mode of ventilation will be used? Which clinical and mechanical measurements contribute to decision-making? How often and in which situations will NIV be used?

**Suggestion -** Assess the particular characteristics of various ventilators as a function of the resources available to and the needs of your service: 

**Ventilators with basic resources**. These include one or more basic modes of ventilation without curves. As a rule, they are used for transportation of patients under MV. 

**Ventilators with basic resources and curves**. These include the basic modes of ventilation (VCV, PCV, SIMV and PSV) and the basic ventilation curves (volume, flow and pressure).

**Ventilators with curves and advanced ventilation resources**. In addition to the basic modes of ventilation and curves, these also include advanced ventilation modes, such as dual-control modes (e.g., PRVC), differential modes for spontaneous ventilation (such as PAV+ and NAVA), and advanced monitoring methods (e.g., measuring the work of breathing, airway occlusion pressure [P 0.1], maximum inspiratory pressure [PImax], volumetric capnometry, and indirect calorimetry). 

**Recommendation -** In the hospital setting, any ventilator should include at least the following features: (1) control of the expired tidal volume (eVt); (2) basic monitoring tools (at least inspiratory pressure); and (3) a gas blender coupled to the ventilator to avoid the use of O_2_ supplementation through the artificial airway. 

**Recommendation -** In addition to the requirements mentioned above, ventilators that are to be used in the ICU should also include the following: (1) curve monitoring (at least the pressure-time curve), (2) alarms (at least for the maximum and minimum airway pressure, for detection of apnea and disconnection from the ventilator).

**Comment -** The electronic supplementary material includes a list of the mechanical ventilators for adults available in Brazil (in August 2013) with a description of some of their features (Tables 2, 3, 4 and 5 in the supplementary material ). This list does not include ventilators that are exclusively used in the following situations: (1) for NIV, (2) in children and newborn infants, (3) at home or for sleep apnea, and (4) in anesthesia. 

## Monitoring the patient under ventilatory support

### Monitoring of gas exchange 

How to perform bedside monitoring of the ventilatory mechanics

**Recommendation -** The ventilatory mechanics should be routinely monitored in all patients who are subjected to invasive mechanical ventilatory support, including the following parameters: eVt, peak pressure (maximum inspiratory pressure), plateau or inspiratory pause pressure (under controlled ventilation), extrinsic PEEP, auto-PEEP or intrinsic PEEP .^(^ 84 ^-^ 88 ^)^

**Suggestion -** Calculate the resistance of airways (Raw) and static compliance (Cst), and monitor the flow-time, pressure-time, and volume-time curves in selected cases.^(^ 84 ^-^ 88 ^)^

**Comment -** In clinical practice, the alveolar pressure can be estimated by means of an inspiratory pause lasting at least two seconds. The pressure at the end of the pause is known as plateau or pause pressure. For measurements to calculate the Raw, the inspiratory flow rate must have a "square" wave pattern and be converted to liters/second. 

**Recommendation -** The following are mandatory requirements for accurate measurement of the pause pressure: absence of respiratory muscle effort, pause duration of two to three seconds, and absence of leaks.^(^ 84 ^-^ 87 ^)^

**Figure 4:**
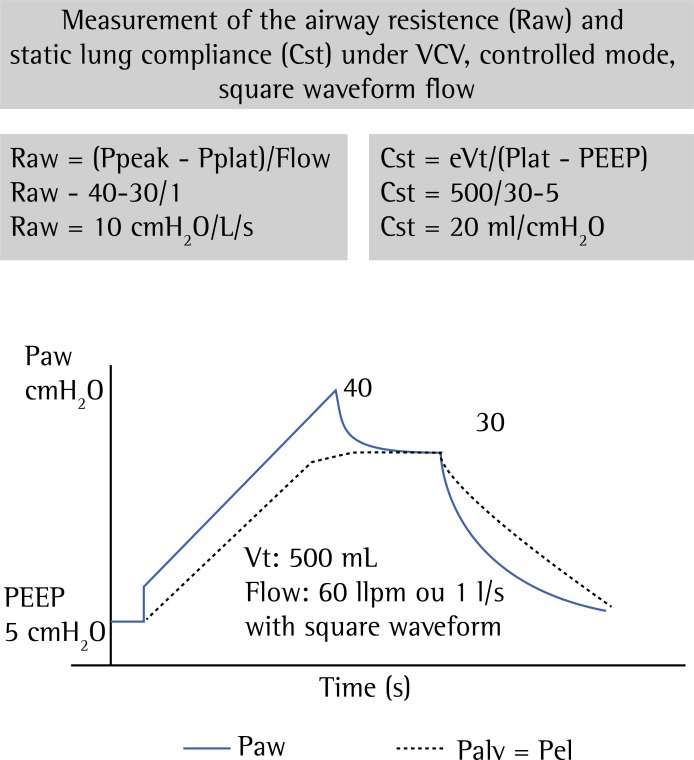
Inspiratory pause maneuver and estimation of the airway resistance and pause (or plateau) pressure. VCV - volume-controlled ventilation. Paw - airway pressure; PEEP - positive end-expiratory pressure; Vt - tidal volume; Pel - elastic pressure; Palv - alveolar pressure.

**Recommendation -** Avoid alveolar pressure values >28 to 30cmH_2_O, which are indicative of low static lung compliance. In such case, the possible cause should be investigated (alteration of the lung parenchyma and/or the thoracic cage). In the former case, reduce the Vt and/or the driving pressure (also called distending pressure); in the latter, also other causes might be present, to wit, reduction of the chest wall compliance and/or intra-abdominal hypertension. In the latter case, the intra-abdominal pressure should be monitored and decompression should be started when needed.^(84-88)^ Figure 4 shows how to calculate Raw and Cst. 

**Comment -** Auto-PEEP, also called intrinsic PEEP (PEEPi), occurs when the end-expiratory pressure is higher than the airway pressure due to incomplete lung emptying. 

**Recommendation - **Auto-PEEP is identified on the flow-time curve when the expiratory flow does not return to zero at the end of expiration. ^(^ 84 ^-^ 87 ^)^

**Recommendation -** Auto-PEEP or PEEPi should be measured during controlled ventilation; for this purpose, a pause is introduced at the end of expiration (expiratory pause), with full attention to the same warnings as in the measurement of the inspiratory pause.^(^ 84 ^-^ 87 ^)^

**Recommendation -** In cases of ARDS, the distending pressure should be monitored; also known as driving pressure*,* this value is calculated by subtracting PEEP from the plateau pressure (Pplat). The distending pressure should always be ≤15cmH_2_O in cases of moderate or severe ARDS, when higher PEEP is necessary, resulting in an increase of Pplat to 30 - 40cmH_2_O (see topic: MV in ARDS in the present recommendations). ^(^ 89 ^-^ 91 ^)^

Monitoring of gas exchange in mechanical ventilation 

Arterial blood gas measurement

**Recommendation - **In order to ground clinical reasoning and therapeutic practice, arterial blood gas samples should be collected as soon as possible, preferably from the radial or the femoral artery, in all cases of ARF. Arterial blood gas assessments permit diagnostic assessment of the acid-base status and lung gas exchange through direct measurement of the pH, PaCO_2_, and PaO_2_, and calculation of the oxygen saturation (SaO_2_), bicarbonate (HCO_3-_) and base excess (BE). When intoxication causing methemoglobinemia and carboxyhemoglobinemia is suspected, SaO_2_ should be directly measured using co-oximetry. ^(^ 92 ^,^ 93 ^)^

**Recommendation -** Collect samples for arterial blood gas measurement in all patients subjected to ventilatory support 20 minutes after the initial adjustment of the ventilator parameters, and then every day for the duration of the acute phase of the clinical problem. Samples should also be collected whenever the patient's clinical condition changes.^(^ 92 ^,^ 93 ^)^

**Recommendation -** Avoid collecting samples for arterial blood gas measurement from areas irrigated by the artery to be punctured that are at risk of ischemia, and from infected sites. In patients with coagulopathy or thrombocytopenia, samples should only be collected when the test is fully necessary.^(^ 92 ^,^ 93 ^)^

Care in the performance of the blood gas measurement

**Suggestion -** Use standard kits or 5-mL syringes with a minimum amount of lithium or sodium heparin, and a fine needle (23 to 25G), preferentially with a safety mechanism.^(^ 92 ^,^ 93 ^)^

**Recommendation -** This procedure is invasive, and thus it must be performed under aseptic conditions. Whenever possible, the procedure should be explained to the patient and performed only with his or her consent.^(^ 92 ^,^ 93 ^)^

**Recommendation -** The puncture site should be compressed for at least five minutes, or longer in cases of coagulopathy or use of anticoagulants. ^(^ 92 ^,^ 93 ^)^

**Recommendation -** The sample should be analyzed as soon as possible. When analysis is performed outside the unit, the sample should be transported in a refrigerated container.^(^ 92 ^,^ 93 ^)^

Care in the interpretation of arterial blood gas measurements

**Recommendation -** Record the following parameters at the time of sample collection: FIO_2_, Vt, f, PEEP, SpO_2_, and ETCO_2_ (when capnography is performed). 

**Recommendation -** The PaO_2_/FIO_2 _ratio should be calculated in all cases to assess the efficiency of oxygenation and the patient's clinical progression.^(^ 92 ^,^ 93 ^)^

**Suggestion - **Record whether the patient is in the prone position, the mode of ventilation at the time of sample collection, and if alveolar recruitment maneuvers and PEEP titration were performed before sample collection.

**Comment -** The arterial blood gas measurement merely reflects a especific moment of the patient's condition. Pulse oximetry and capnography are more adequate methods for continuous monitoring.

Pulse oximetry

**Recommendation -** Continuous monitoring by means of pulse oximetry should be performed in all patients who are receiving O_2_ supplementation, NIV, or invasive ventilatory support, as well as in patients with ARF.

Capnography

**Recommendation -** Perform capnography in patients with neurologic diseases who are receiving ventilatory support, to confirm the position of the ventilatory prosthesis, and whenever the CO_2 _level is above 50mmHg. 

**Suggestion -** Capnography can be used for monitoring in patients with a ventilation-perfusion imbalance to detect acute alterations in status, as well as for monitoring of specific therapies (e.g., thrombolytic therapy in pulmonary thromboembolism). 

### Regional monitoring

Monitoring by means of electrical impedance tomography

**Comment -** Electrical impedance tomography (EIT) is a noninvasive technique based on the measurement of electrical current that passes between electrodes placed around the thorax to identify areas that are more and less resistant to the passage of the current. EIT is used for monitoring ventilation, and more recently, for bedside continuous monitoring of lung perfusion.^(^ 94 ^-^ 98 ^)^

**Suggestion -** Use EIT for detection of lung ventilation disorders, such as pneumothorax, as well as for evaluating changes in ventilation when placing the patient in specific decubitus position, to check the position of the endotracheal tube, to assess pulmonary recruitment and collapse, and to assess the regional distribution of ventilation. In the future, EIT may be used for monitoring of lung perfusion.^(^ 94 ^-^ 98 ^)^

Computed tomography

**Recommendation -** Use computed tomography (CT) as a diagnostic method in cases of respiratory failure of unknown etiology; CT angiography should be used when pulmonary embolism is suspected. 

**Suggestion -** In centers where CT is available, this method may be used to monitor alveolar recruitment and decremental PEEP titration in cases of moderate or severe ARDS, paying special attention to the care required in patient's transportation, and taking the total radiation dose into consideration.^(^ 99 ^,^ 100 ^)^

Chest ultrasound

**Recommendation -** In centers where it is available, staff should be trained to use chest ultrasound for early detection of pneumothorax and pleural effusion, and as an aid in performing therapeutic procedures. 

**Suggestion -** Chest ultrasound can be used to estimate alveolar re-aeration in patients treated for VAP, to assess pulmonary edema, to detect post-extubation atelectasis, and to estimate PEEP-induced pulmonary recruitment.^(^ 101 ^-^ 103 ^)^

## Sedation and analgesia during mechanical ventilation

When are sedatives and analgesics indicated and how should they be administered?

**Suggestion -** Use sedation and analgesia in patients treated with MV in order to control anxiety, agitation and pain. Appropriate sedation helps the patient better tolerate the ventilator, diagnostic and therapeutic procedures.^(^ 104 ^,^ 105 ^)^

**Recommendation -** The sedation level should be mild to moderate to allow for early mobilization. ^(^ 106 ^)^

**Recommendation -** Titrate propofol and midazolam for low, moderate and deep sedation. Dexmedetomidine should not be used to induce deep sedation. The recommended opioids are fentanyl, morphine, and remifentanil.^(^ 107 ^)^

**Suggestion -** Avoid using ketamine as the main sedative in patients undergoing MV. Ketamine may be useful in situations in which its opioid-sparing effect is required.^(^ 107 ^,^ 108 ^)^

**Suggestion - **Have a thorough knowledge of the main drugs used for analgesia and sedation in patients under ventilatory support:

**Propofol -** Its main action is as a gamma-aminobutyric acid (GABA) agonist. It has sedative, hypnotic, anxiolytic and anticonvulsant effects and promotes amnesia. It does not have an analgesic effect. It causes dose-dependent respiratory depression and hypotension secondary to systemic vasodilation, especially when administered by bolus. Prolonged infusion might make awakening unpredictable and cause propofol infusion syndrome (PRIS), which has an incidence of <1%. The mortality of PRIS is high, and the syndrome is characterized by worsening of metabolic acidosis, hypertriglyceridemia, arrhythmia, and hypotension with an increased need for vasopressors. The recommended initial dose is 5mcg/kg/minute over five minutes, followed by continuous infusion at 5 to 50mcg/kg/minute.^(^ 107 ^)^

**Midazolam -** This is a GABA agonist that promotes anxiolysis, amnesia and hypnosis. It has anticonvulsant effects. It does not have an analgesic effect. The use of this benzodiazepine for hypnosis seems to be associated with a higher incidence of delirium. Compared to propofol, midazolam may increase the length under MV. Abstinence syndrome can occur after prolonged infusions, i.e., longer than seven days. The recommended initial dose is 0.01 to 0.05mg/kg, and the maintenance dose is 0.02 to 0.1mg/kg/h in continuous infusion.^(^ 109 ^)^

**Dexmedetomidine -** This is an alpha-2-adrenergic agonist with central action. It has sedative effects and helps to reduce the need for analgesics/opioids. It does not have an anticonvulsant effect. It is not associated with significant respiratory depression. It is not appropriate for inducing deep sedation. The prevalence of delirium is lower in patients treated with dexmedetomidine compared to benzodiazepines. In patients admitted to the ICU, the recommended loading dose is not used, and the drug is started as a continuous infusion. After the start of infusion, its action begins in 15 minutes, and the maximum effect is reached within one hour. The recommended dose is up to 1.4mcg/kg/hour.^(^ 107 ^)^

**Fentanyl - **This has rapid onset of action and high potency. It does not release histamine. It tends to accumulate in parallel with the duration of continuous infusion and in patients with liver dysfunction; in some patients, chest-wall rigidity can occur. The recommended initial dose is 50 to 100mcg. For continuous infusion, the recommended rate is 0.7 to 10mcg/kg/h.^(^ 108 ^,^ 110 ^)^

**Morphine -** This is the opioid that is most widely used for pain exacerbations. For bedside titration, 1 to 2mg are administered every 10 minutes until adequate analgesia is achieved or side effects appear. For continuous infusion, the recommended dose is 2 to 30mg/h. It tends to accumulate in case of liver or kidney dysfunction. It releases histamine.^(^ 110 ^)^

**Remifentanil -** This is an opioid with analgesic potency similar to that of fentanyl. It is metabolized by plasma esterases, and its pharmacological profile does not favor accumulation, even after prolonged infusion. It does not exhibit a residual analgesic effect. The recommended loading dose is 1.5mcg/kg in about three to five minutes, and the recommended maintenance dose is 0.5 to 15mcg/kg/hour.^(^ 110 ^)^

### When should neuromuscular blocking agents be used?

**Recommendation** - Use cisatracurium during the first 48 hours in cases of ARDS with PaO_2_/FiO_2_<120 to maintain controlled MV. Induction of neuromuscular blockade requires deep sedation and appropriate monitoring of the level of consciousness. The suggested dose is 37.5mg/hour.^(^ 111 ^)^

### How should sedated patients under mechanical ventilation be monitored?

**Recommendation -** Monitor the level of sedation using the Sedation and Agitation Scale (SAS) or the Richmond Agitation and Sedation Scale (RASS). Both are tools for clinical use that have been validated for use in the ICU and must be applied systematically, by trained staff.^(^ 112 ^,^ 113 ^)^

**Suggestion -** In order to assess the degree of sedation in patients who require neuromuscular blockade or when the use of scales is not possible, use brain activity monitoring methods such as continuous electroencephalography (EEG) or the biespectral index system (BIS).

### How to discontinue sedation 

**Recommendation -** Perform daily interruption of sedation in patients receiving MV as soon as the patient's clinical condition allows it. Patients who are awake or might be easily awakened and cooperative with the current sedation strategy do not require discontinuance or interruption of sedation.^(^ 110 ^,^ 114 ^)^

**Recommendation -** Pain and delirium should be routinely and frequently assessed and treated, as they commonly cause agitation upon awakening. Maintenance of sedation can contribute to increased MV duration and difficult weaning from MV.^(^ 110 ^)^

## Mechanical ventilation in asthma

**Comment - **Severe asthma attacks pose a risk to the patient's life. Morbidity and mortality due to asthma has decreased in the past decade as a function of the use of ventilatory strategies that aim to reduce alveolar hyperinflation.^(^ 115 ^)^

### Indications for mechanical ventilation

**Recommendation -** Indications for invasive MV in asthma include the following: cardiac arrest; respiratory arrest; reduced level of consciousness, Glasgow Coma Scale <12; hypoxemia (PaO_2_<60 mmHg; SpO_2_<90%) that is uncorrected by use of face-mask oxygen supplementation (FiO_2 _40-50%); severe arrhythmia; or progressive fatigue (progressive hypercapnia).

**Suggestion -** Suggested indications for invasive MV in asthma include myocardial ischemia and lactic acidosis after treatment with bronchodilators.^(116,117)^

### Intubation of patients with an asthma attack

**Recommendation - **Perform rapid sequence intubation. 

**Suggestion -** Place the patient in a 20-30º-degree, head-up position (which reduces the risk of passive regurgitation and aspiration).

**Recommendation -** Perform pre-oxygenation with O_2_ mask or BIPAP; use a bag-valve mask gently (eight respiratory cycles).

**Suggestion -** Perform premedication with intravenous (IV) lidocaine at 1.5mg/kg, three minutes before intubation (this reduces the sympathetic reflex and the occurrence of nausea and vomiting) and fentanyl 3mcg/kg (this reduces the sympathetic reflex but may cause respiratory depression). 

### **Suggestion - **Do not perform the Sellick maneuver. 

**Recommendation -** For inducing intubation, select ketamine 1 to 2mg/kg IV, propofol 2 to 2.5mg/kg IV, or etomidate 0.2 to 0.3mg/kg IV.

**Recommendation -** To induce muscle relaxation, use rocuronium 0.9mg/kg or succinylcholine 1 to 1.5mg/kg IV (fasciculation may increase the risk of regurgitation and aspiration). ^(^ 118 ^,^ 119 ^)^

**Suggestion -** Another option for inducing muscle relaxation is vecuronium 0.3mg/kg (disadvantage: onset of action is 60-90 seconds).

**Suggestion -** Use a tube with the largest possible diameter (>8 mm of internal diameter when possible). 

### Ventilator settings

**Suggestion - **ventilatory settings are as follows: Mode: PCV or VCV; Vt: 6mL/kg predicted body weight; maximum inspiratory pressure: <50cmH_2_O; plateau pressure: <35cmH_2_O; auto-PEEP: <15cmH_2_O; f: 8 to 12 breaths/minute; flow rate: as needed to maintain an expiratory time sufficient to end expiration; 60 to 100L/minute (VCV); free (PCV); FiO_2_: as necessary to maintain SpO_2_>92%; PaO_2_>60mmHg; PEEP: low (3 to 5cmH_2_O); in selected cases (however, with appropriate monitoring, higher values of PEEP can be used, due to its mechanical effect of opening the small airways).^(116,117)^

### Patient monitoring and reduction of hyperinflation

**Recommendation - **Patients with asthma who are receiving MV should be periodically monitored for alveolar hyperinflation (plateau pressure and intrinsic PEEP) and calculation of the airway resistance. The peak pressure is not a representative measure of alveolar hyperinflation. ^(^ 120 ^,^ 121 ^)^

**Recommendation -** Use a Vt of 5 to 6mL/kg predicted body weight. In cases with hyperinflation refractory to conventional treatment, consider volumes <5mL/kg and lower f (10 to 12 breaths per minute) to avoid alveolar hyperinflation. This strategy may cause hypercapnia; therefore, the PaCO_2 _should be monitored and maintained <80mmHg, and the pH should be maintained >7.20 (permissive hypercapnia). 

**Suggestion -** Use PEEP as a strategy to reduce alveolar hyperinflation. In such cases, use PCV with a distending pressure of ≤15cmH_2_O. When PEEP increases, the expiratory volume may increase, which indicates a reduction in alveolar hyperinflation, or deflation.

**Recommendation -** Monitor ventilatory mechanics in patients who have hemodynamic instability in order to detect the presence of auto-PEEP, and readjust the parameters as needed to improve hemodynamic status.^(^ 122 ^)^

**Recommendation -** Perform chest radiographs in patients with hemodynamic instability, due to the risk of pneumothorax.^(120,121)^

**Recommendation -** Weaning from ventilation must be started as soon as bronchospasm and alveolar hyperinflation are controlled.^(^ 1 ^,^ 8 ^)^

**Suggestion -** Patients with asthma can be extubated under mild sedation.

**Suggestion -** In cases where ventilator weaning is difficult, investigate the presence of respiratory muscle weakness due to polyneuropathy associated with the use of corticoids and curare. 

## Analgesia and sedation(123-126)

**Suggestion -** Avoiding the use of morphine is suggested, as morphine may increase histamine release. Do not use meperidine, because it also may increase histamine release. The following agents can be used: fentanyl 1 to 3mcg/kg/hour; alfentanil 0.5 to 1mcg/kg/minute; sufentanil 0.5mcg/kg/hour; ketamine 0.25 to 0.5mg/kg/hour (bronchodilator); propofol 0.3 to 4mg/kg/hour (bronchodilator); or midazolam 0.04 to 0.06mg/kg/hour (3 to 5mg/hour). 

## Muscle relaxation(126-128)

**Recommendation - **Muscle relaxation may be performed as needed to allow intubation during the initial stage of MV. Long-lasting use should be avoided due to an associated risk of myopathy and neuropathy (a risk that is increased by the concomitant use of corticoids). 

**Recommendation -** Rocuronium is the drug of choice, with a dose of 1mg/kg, an onset of action 45 seconds, and duration of action of 45 minutes. Sugammadex can be used as an antidote, if needed.

**Suggestion - **The suggested muscle relaxants for use during MV are vecuronium (0.15mg/kg, onset of action 75-90 seconds, and duration of action 30 minutes) or succinylcholine at a dose of 1 to 1.2mg/kg (up to 1.5mg/kg) for intubation at induction. Succinylcholine is contraindicated in patients with a history of malignant hyperthermia, neuromuscular disease, muscular dystrophy, hyperkalemia, or rhabdomyolysis, as well as for use up to 72 hours after burns or up to 72 hours after stroke.

**Suggestion - **Do not use pancuronium**; **although the risk of histamine release is low, it is higher than that of vecuronium or rocuronium. 

**Recommendation - **Do not use atracurium or cisatracurium due to the high risk of histamine release.

Additional treatment - use of anesthetics, heliox and extracorporeal membrane oxygenation 

**Suggestion -** Use halogen-based anesthetics (e.g., isoflurane) administered through an anesthesia ventilator for possible control of bronchospasm that is refractory to usual treatment; this therapy should not be used for longer than 12 hours. Special attention should be paid to monitoring for liver injury during their use.^(^ 122 ^)^

**Suggestion - **Heliox may reduce airway resistance and facilitate the delivery of bronchodilators to the lungs; its use can be attempted in refractory cases and in services where the appropriate equipment for the use of heliox is available.^(^ 129 ^)^

**Suggestion -** Extracorporeal membrane oxygenation (ECMO) can be considered for severe cases that do not respond to the abovementioned treatments.^(^ 130 ^)^

## Mechanical ventilation in chronic obstructive pulmonary disease

### Indications for invasive mechanical ventilation

**Recommendation -** Consider invasive MV when NIV is contraindicated or fails (25% of cases). Optimize pharmacological treatment. 

**Suggestion -** For OTI, use tubes with the largest possible diameter, ideally > 8 mm, to reduce airway resistance and facilitate the removal of secretions.^(^ 131 ^-^ 133 ^)^

### Aims of mechanical ventilation

**Recommendation -** To promote respiratory muscle rest and improvement of acute gas exchange disorders, reduce lung hyperinflation, and optimize patient-ventilator synchrony.^(^ 131 ^,^ 134 ^,^ 135 ^)^

### Initial mode of ventilation 

**Suggestion -** Any mode of ventilation (volume- or pressure-controlled) can be used in the initial treatment of COPD exacerbations, provided monitoring is adequate and the staff is thoroughly acquainted with the selected mode. ^(^ 131 ^-^ 136 ^)^

### Fraction of inspired oxygen

**Suggestion -** Adjust FiO_2_ based on the arterial blood gas measurement and pulse oximetry so as to use the lowest FIO_2_ level that can maintain SaO_2_ at 92 to 95% and PaO_2_ at 65 to 80mmHg. ^(^ 131 ^)^

### Tidal volume

**Recommendation -** Use a low Vt, specifically 6mL/kg predicted body weight.^(131,132-136) ^In PCV and PSV, monitor for excess Vt that can occur when pressure levels are low. 

### Ventilation frequency and minute volume

**Recommendation - **Initially set f at 8 to 12 breaths per minute. Minute volume should be adjusted to achieve a normal pH, rather than a normal PaCO_2_.^(^ 131 ^,^ 132 ^-^ 136 ^)^

### Inspiratory flow and inspiration-to-expiration ratio

**Recommendation -** In the controlled-volume mode, use a decelerating inspiratory flow rate of 40 to 60L/min, and adjust the I:E rate to <1:3, thus allowing for an expiratory time long enough to promote pulmonary deflation and reduce air trapping. In the pressure-controlled mode, set the lowest distending pressure value that allows for an inspiratory time sufficient to reduce the ventilator inspiratory flow to zero (lung filling time). The I:E ratio should be kept at <1:3 to achieve a sufficient expiratory time with minimal auto-PEEP.^(^ 131 ^,^ 132 ^-^ 136 ^)^

### Use of PEEP in controlled ventilation

**Suggestion -** Apply external PEEP to counterbalance auto-PEEP caused by the expiratory flow limitation, or as an attempt to induce lung deflation, provided that respiratory mechanics are adequately monitored. For this purpose, the plateau pressure value should be used in the VCV and PCV modes.^(131,137,138) ^In VCV, external PEEP-induced deflation is determined by the maintenance or fall of the plateau pressure. However, when the plateau pressure increases, external PEEP can cause additional lung hyperinflation and thus should be reduced or discontinued. In PCV, the expired Vt should be monitored in parallel with the increase in PEEP. When the expired Vt decreases, hyperinflation becomes worse, and external PEEP should be reduced or discontinued. On the contrary, when the expired Vt increases, external PEEP induces lung deflation and can be maintained.^(^ 131 ^,^ 137 ^,^ 138 ^)^

### Use of PEEP in assisted/spontaneous ventilation 

**Suggestion -** In the case of pressure-triggered ventilation, patients with auto-PEEP may find it difficult to start an assisted cycle, thus resulting in asynchrony. In such cases, flow-triggering should be used and/or external PEEP should be applied at approximately 85% of auto-PEEP to help the patient reach the ventilator trigger threshold.^(^ 134 ^,^ 139 ^,^ 140 ^)^

### Monitoring of mechanical ventilation 

**Recommendation -** In COPD exacerbations, the respiratory mechanics and lung hyperinflation should be monitored. The main parameters to monitor are: plateau pressure, peak pressure, auto-PEEP, airway resistance, as well as the flow-time, volume-time, and pressure-time curves. In cases with severe bronchospasm, a peak pressure as high as 45cmH_2_O may be well tolerated, provided the plateau pressure is <30cmH_2_O.^(^ 131 ^,^ 132 ^)^

### Discontinuation of mechanical ventilation 

**Suggestion -** Patients with COPD usually have greater difficulty in achieving an appropriate patient-ventilator interaction. Therefore, it is suggested to use modes of ventilation that afford greater comfort to the patient and facilitate monitoring. In this regard, PSV is quite useful when it is properly set. Special attention should be paid to high support pressure values, as they can hinder cycling and worsen the patient-ventilation interaction, resulting in increased auto-PEEP. PAV+ and NAVA are promising approaches for improving the patient-ventilator interaction, but more evidence is needed before these modes are routinely recommended.^(^ 141 ^-^ 143 ^)^

**Suggestion -** The deceleration of the inspiratory flow rate is lower in patients with COPD, and the inspiratory time may be increased in PSV with the usual expiratory sensitivity (25%). In ventilators that allow adjustment of PSV cycling (percentage of the cycling criterion, expiratory sensitivity or cycling-off criteria), adjust the expiratory cycling sensitivity to a higher level (40% to 60%), aiming to reduce the inspiratory time, Vt and the odds of asynchrony.^(^ 144 ^,^ 145 ^)^

**Suggestion -** To reduce the inspiratory time, with a consequent increase in the expiratory time, adjust the inspiratory flow rise time to a higher level, taking proper care to avoid inspiratory flow overshoot and monitor the patient's comfort. ^(^ 144 ^,^ 145 ^)^

**Recommendation -** After 24 to 48 hours of muscle rest, use NIV for early discontinuation of invasive MV in patients with COPD exacerbations provided the staff is duly trained and the criteria described in the specific corresponding topic in the present recommendations are followed.^(^ 146 ^)^

### Administration of inhaled bronchodilators 

**Suggestion -** Administer bronchodilators per the inhalation route using nebulizers or a metered-dose spray coupled to a spacer. Advantages of the metered-dose spray include ease of manipulation, a reproducible dose, and a lower risk of contamination.^(147) ^When beta-2-adrenergic agonists are administered using a metered-dose spray, the suggested dose is four puffs (first at 20-minute intervals for to three doses, and every two to four hours as maintenance treatment).^(^ 148 ^)^

## Mechanical ventilation in community-acquired pneumonia

**Comment - **The following recommendations apply to patients with CAP and healthcare-associated pneumonia (HCAP) and concern invasive mechanical ventilation and NIV. In the case of pneumonia associated with ARDS, see the specific topic in the present recommendations.

### Noninvasive mechanical ventilation

**Suggestion -** Use NIV cautiously in individuals with severe pneumonia. Use of NIV should be monitored at the bedside by a healthcare professional within thirty minutes to two hours. For NIV to be considered successful, the following criteria should be met: reduction of f, increase of Vt, improvement of the level of consciousness, reduction or cessation of the use of the respiratory accessory muscles, increase of PaO_2_ and/or SpO_2_, and reduction of PaCO_2_ without significant abdominal distension. In unsuccessful cases, OTI and invasive MV should be performed immediately, as delay in intubation reduces the patient's' survival. A better response to NIV is achieved under the following three circumstances: patients with systolic or diastolic left-sided cardiac failure; COPD with CO_2_ retention and acidosis; and immunosuppressed individuals with bilateral pneumonia. Success is expected in 75% of patients with hypercapnia and 50% of patients with hypoxia.^(^ 149 ^,^ 150 ^)^

### Mode of ventilation 

**Suggestion -** The choice of the mode of ventilation^(151-154) ^should be based on three criteria: the multi-professional staff's knowledge of and skills in using the selected mode; ventilator availability; and the clinical indication, which is mainly based on the presence of respiratory stimulus, hemodynamic instability and the severity of the lung injury. 

### Positive end-expiratory pressure

**Suggestion -** In the absence of ARDS, use PEEP values of 5 to 10cmH_2_O. The PEEP value should be adjusted in combination with the FiO_2_ to keep SpO_2_ at 90 to 95%, in order to minimize the risk of cognitive impairment. MV with very low or no PEEP is associated with greater bacterial translocation.^(151-155) ^For patients with ARDS, see the specific topic in the present recommendations.

### Adjusting the fraction of inspired oxygen 

**Suggestion -** The FiO_2_ value should be adjusted in combination with PEEP in order to keep SpO_2_ at 90 to 95%, thus minimizing the risk of cognitive impairment.^(^ 151 ^-^ 155 ^)^

### Tidal volume

**Suggestion - **A Vt of >6mL/kg ideal body weight increases bacterial translocation and ventilator-associated lung injury. Therefore, patients should be ventilated with a Vt of ≤6mL/kg predicted body weight.^(^ 151 ^-^ 155 ^)^

### Decubitus

**Suggestion -** Patients with unilateral pneumonia and severe hypoxemia can be placed in the lateral decubitus position. However, close surveillance is needed in such cases given the unpredictability of the results, as there is a higher risk of worse oxygenation and contamination of the contralateral lung.^(^ 156 ^)^

### Rescue treatment

**Suggestion -** Patients with unilateral pneumonia and hypoxemia that is refractory to conventional treatment may be candidates for independent MV. This treatment, however, should be performed only at centers with wide experience in independent lung ventilation and an available bronchoscopy service.^(^ 157 ^)^

## Ventilator-associated pneumonia

**Suggestion -** Patients with VAP should be ventilated using a protective ventilation strategy (Vt=6mL/kg predicted body weight), f to maintain PaCO_2_ at 35 to 45mmHg, and PEEP sufficient to ensure appropriate gas exchange, with either the VCV or PCV modes). Shift to assisted or spontaneous modes as soon as possible to achieve earlier discontinuation of MV. 

**Suggestion -** Patients with unilateral pneumonia and severe hypoxemia can be placed in the lateral decubitus position. However, close surveillance is needed in such cases given the unpredictability of the results, as there is risk of worse oxygenation and contamination of the contralateral lung. New proposals for positioning to reduce the aspiration of secretion above the cuff and prevent VAP are currently being investigated, such as the Trendelenburg lateral decubitus position.^(^ 158 ^)^

**Recommendation** - Use the following general strategies to reduce VAP: wash/disinfect the hands with 70% alcohol; perform microbiological surveillance; monitor and remove invasive devices as soon as possible; and apply programs for the rational use of antibiotics. 

**Recommendation -** The ventilator circuits should be replaced when they become dirty or damaged, since there is no need for planned replacement. Replacement of humidifiers must be performed every seven days or as needed. 

**Recommendation -** Perform aspiration of subglottic secretions when the patient requires MV for more than 72 hours; ventilation can be intermittent or controlled by a device that is specifically designed for this purpose.^(^ 159 ^)^

**Suggestion -** When available, use tubes with cuffs that are specifically designed to avoid microaspiration in patients who are estimated to require MV for at least 24 hours.

**Recommendation -** Set, monitor, and maintain the endotracheal tube cuff pressure at a level of at least 25cmH_2_O.^(^ 160 ^)^

**Recommendation -** The patient should be placed in the 30-45º-degree, head-up position.

**Recommendation -** Perform oral hygiene with 2% chlorhexidine on a daily basis.^(^ 161 ^)^

**Suggestion -** Perform daily interruption of sedation.

**Suggestion -** Perform selective decontamination of the digestive tract.^(^ 162 ^,^ 163 ^)^

**Recommendation -** Use silver-coated endotracheal tubes whenever intubation is estimated to last more than 24 hours.^(^ 164 ^)^

## Mechanical ventilation in patients with sepsis

**Comment -** ARDS is a common complication in patients with severe sepsis, although it is underdiagnosed in most cases. Observational studies showed that a diagnosis of ARDS is registered in the clinical records of only 30% to 50% of individuals who were shown to have diffuse alveolar injury on autopsy.^(165-167) ^For this reason, special attention to the possible presence of ARDS in patients with sepsis has paramount importance. Some interventions have proven efficacy in patients with ARDS, such as ventilation with Vt between 4 to 6mL/kg of predicted body weight. However, these interventions still need to be more widely disseminated, applied, and audited in clinical practice.^(168-170) ^Lack of appropriate diagnosis is a possible reason for the low rates of institutional adherence to appropriate treatment. ^(^ 166 ^)^

**Suggestion -** Apply a routine system for identifying ARDS in patients with sepsis, particularly in patients with severe sepsis and septic shock. A decrease in the PaO_2_/FiO_2_ ratio and the presence of bilateral infiltrates on the chest radiograph as diagnostic criteria may be possible diagnostic criteria for ARDS; clinicians should also monitor patients for signs that may represent early manifestations of ARDS^(171,172) ^(increased f, decrease of SpO2 and need for O_2_ supplementation) as early alerts.

**Observation - **The diagnosis and management of ARDS are described in sections "Mechanical ventilation in ARDS" and "Ventilation in the prone position and extracorporeal circulation" of the present recommendations. 

**Recommendation -** Use a Vt of approximately 6mL/kg predicted body weight in patients who are undergoing MV and have sepsis but not ARDS. A systematic review that included randomized and observational studies on patients who underwent surgery or were admitted to the ICU suggests that ventilation at a low Vt is associated with reduced mortality, as well as a reduced incidence of ARDS and pneumonia, compared to ventilation at a high Vt.^(^ 52 ^)^

**Observation -** Recommendations for patients undergoing MV with pneumonia and sepsis but without ARDS, are included in the section "Mechanical ventilation in pneumonia". 

## Mechanical ventilation in acute respiratory distress syndrome: diagnosis, recommendations and special care

**Comment - **Starting in 2012, ARDS was classified (Berlin Definition) into three categories (mild, moderate and severe)^(173) ^(Chart 6). 

**Quadro 6:**
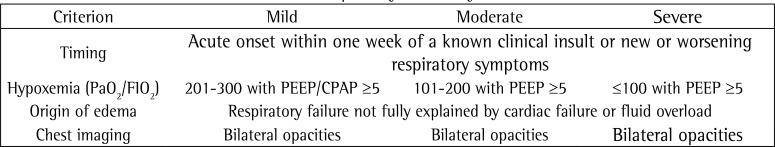
Berlin classification of acute respiratory distress syndrome(173) PaO2/FIO2 - relationship between oxygen partial pressure and fraction of inspired oxygen; PEEP - positive end-expiratory pressure; CPAP - continuous positive airway pressure.

### How to ventilate patients with ARDS 

Modes of ventilation

**Recommendation -** Initially (first 48 to 72 hours), controlled modes of ventilation (VCV or PCV) are recommended for all patients with ARDS (i.e., mild, moderate and severe cases). In PCV, the airway pressure is equal to the plateau or alveolar pressure when the respiratory flow falls to zero. 

### Tidal volume(55,174,175)

**Recommendation -** In patients with mild ARDS who require assisted ventilation, Vt should be set at 6mL/kg (predicted body weight).

**Recommendation -** In patients with moderate or severe cases of ARDS who require assisted or controlled ventilation, Vt should be set at 3 to 6mL/kg (predicted body weight).^(^ 176 ^)^

**Recommendation -** Use the following formulas to calculate the predicted body weight:^(^ 175 ^)^ males: 50 + 0.91 x (height in cm - 152.4); females**:** 45.5 + 0.91 x (height in cm - 152.4).

### Fraction of inspired oxygen

**Recommendation -** Use the lowest possible FiO_2 _that suffices to ensure SpO_2_>92% in all three ARDS categories.

### Plateau pressure

**Recommendation -** Try to maintain the plateau pressure (Pplat) ≤ 30cmH_2_O.^(175,177)^

**Recommendation -** Try to keep the difference between Pplat and PEEP (known as distending pressure or driving pressure) ≤15cmH_2_O in all three ARDS categories.^(91)^

**Suggestion -** When high PEEP (usually >15cmH_2_O) is used in patients with moderate or severe ARDS, a Pplat of up to 40cmH_2_O may be tolerable, provided that the driving pressure is always maintained at ≤15cmH_2_O.^(^ 91 ^)^

### Respiratory rate

**Recommendation -** Begin with f = 20 breaths per minute, and increase to up to 35 breaths per minute as needed in order to achieve the desired PaCO_2_ (<80mmHg), provided that auto-PEEP is not induced. In patients with moderate or severe ARDS who are subjected to permissive hypercapnia with a Vt of ≤6mL/kg predicted body weight, f may be as high as 45 breaths per minute, provided that this f does not cause auto-PEEP.^(^ 175 ^)^

### PEEP adjustment

**Comment -** There are several strategies for adjusting PEEP in ARDS, many of which are in equipoise (i.e., the degree of evidence does not allow for a definitive conclusion on the superiority of any of them). The techniques where there is wider experience and that have proved to be safer in clinical studies are described in this topic.

**Recommendation -** Avoid using PEEP <5cmH_2_O in patients with ARDS.^(^ 55 ^,^ 175 ^)^

**Recommendation** - Avoid using PEEP below the values described in the table "LOW PEEP versus FIO_2_" (Table 1).^(175)^

**Table 1:** PEEP versus FiO2 to identify optimal PEEP in cases of mild ARDS

FIO_2_	0.3	0.4	0.4	0.5	0.5	0.6	0.7	0.7	0.7	0.8	0.9	0.9	0.9	1.0
PEEP	5	5	8	8	10	10	10	12	14	14	14	16	18	18↔24


**Adapted from:** : Ventilation with lower tidal volumes as compared with traditional tidal volumes for acute lung injury and the acute respiratory distress syndrome

The Acute Respiratory Distress Syndrome Network. N Engl J Med. 2000;342(18):1301-8

FiO2 - fraction of inspired oxygen

PEEP - positive end-expiratory pressure.

### **Suggestion -** Use the table "LOW PEEP versus FIO_2_" (Table 1) only in cases of mild ARDS.^(175)^

**Comment -** There are two options for adjusting high PEEP, corresponding to the ALVEOLI^(178) ^and LOVS^(^ 179 ^)^ studies that are described in Table 2; these studies demonstrated very similar practical results. The LOVS table tends to subject the patient to longer periods of high PEEP. 

**Table 2:** Adjustment of PEEP at high values to find the optimal PEEP in cases of moderate or severe ARDS

Study ALVEOLI table																
FIO_2_	0.3	0.3	0.4		0.4		0.5	0.5		0.5↔0.8		0.8		0.9		1.0
PEEP	12	14	14		16		16	18		20		22		22		22↔24
**Study LOVS table**																
FIO_2_	0.3	0.4		0.5		0.6			0.7		0.8		0.9		1.0	
PEEP	5↔10	10↔18		18↔20		20			20		20↔22		22		22↔24	

**Source:** : based on studies ALVEOLI(178) and LOVS(179). FiO2 - fraction of inspired oxygen; PEEP - positive end-expiratory pressure.

**Suggestion -** Use Table 2 in cases of moderate or severe ARDS as an alternative to the decremental PEEP technique, which is described below.

**Suggestion -** The Express study suggests using Pplat of a maximum of 30 cmH_2_O and maximum PEEP with a Vt of 6 mL/kg predicted body weight in cases of moderate or severe ARDS.^(^ 180 ^)^

**Suggestion -** Avoid using Table 2 in cases of mild ARDS.^(^ 181 ^)^

Decremental PEEP titration according to respiratory system compliance

**Recommendation -** The decremental PEEP technique is described in the following section. After a maximum recruitment strategy (MRS) is performed, the elastic compliance of the respiratory system is measured at decremental PEEP values starting at 23 to 26cmH_2_O to a minimum of approximately 8 to 12 cmH_2_O. PEEP is typically decreased in steps of 2 to 3cmH_2_O every four minutes. Once the PEEP level that induces the best compliance is identified, or two or more reduction steps with equivalent compliance are observed, a PEEP value 2 to 3cmH_2_O above that level is selected. Before the PEEP value thus found to be adequate is finally selected, a MRS is performed again. PEEP may then be directly set at 2 to 3cmH_2_O above the value identified by decremental titration.^(^ 182 ^-^ 184 ^)^

**Suggestion -** Consider MRS in cases of moderate or severe ARDS.^(^ 182 ^-^ 184 ^)^

Decremental PEEP titration by other methods 

**Suggestion -** Perform decremental PEEP titration using EIT in centers where it is available: following MRS, select the PEEP value that is associated with a collapse increase of less than 5%, as estimated by EIT.^(^ 95 ^)^

**Suggestion -** Perform decremental PEEP titration using conventional CT. Following MRS, select the PEEP value that is associated with a collapse increase of less than 5%, as estimated by CT. If this method is used, all issues related to patient care, transportation, and safety should be taken into consideration, only staff who have been specifically trained for this procedure should be involved, and low radiation doses should be used.^(^ 91 ^)^

**Suggestion -** Based on oxygenation after MRS, select the PEEP value that is associated with a <10% reduction in the PaO_2_/FIO_2_ ratio. 

Estimation of the lower inflection point using the random volumes technique

**Recommendation** - With the patient sedated and without an active ventilatory drive, set PEEP to zero and vary the Vt in 50mL aliquots to a maximum Vt of 1,000mL or a Pplat of 40cmH_2_O, and record the Pplat value after three ventilations. Record the measurements in a Vt versus Pplat table and plot an x-y graph (Vt on the y-axis and Pplat on the x-axis). A sigmoid curve is expected. Identify the curve trends and the lower inflection point (meeting point of the trend lines in the first curvature, projecting the value on the x-axis) and set PEEP 2.0cm above the lower inflection point.^(^ 55 ^)^

Estimation of the point of best compliance (compliance-PEEP technique) 

**Recommendation -** With the patient sedated and without active ventilatory drive*, *set Vt at 6mL/kg predicted body weight, and vary PEEP in 2 to 3cmH_2_O aliquots; record Pplat after three ventilations. Record the measurements in a PEEP versus static compliance table (for how to calculate the static compliance of the respiratory system (Cst), see section "Monitoring the patient under ventilatory support" in the present recommendations) to find the PEEP value that provides the best Cst. Set PEEP 2.0 cm H_2_O above that value. If the best Cst value corresponds to two PEEP levels, the ideal PEEP should be considered to be the highest one. 

### Neuromuscular blocking agents

**Recommendation -** In cases of ARDS with pO_2_/FiO_2_<120mmHg under deep sedation, use cisatracurium during the first 48 hours of ventilatory support.^(^ 111 ^)^

### Prone positioning

**Recommendation -** Use prone positioning in patients with ARDS and a PaO2/FiO2 ratio of <150 for at least 16 hours per session. (More details are provided in the specific topic of the present recommendations).^(^ 185 ^)^

**Recommendation -** Discontinue prone positioning as soon as a PaO2/FiO2 ratio >150mmHg is attained with a PEEP of ≤10cmH_2_O in the supine position.^(^ 17 ^)^

**Suggestion - **In patients with moderate or severe ARDS, use prone positioning for patients with right ventricular dysfunction and controlled hypoxemia, as well as in cases where it is difficult to maintain lung protection within the safety threshold (distending pressure ≤15cmH_2_O and pH >7.15).^(^ 185 ^,^ 186 ^)^

Maximum alveolar recruitment maneuvers or maximum recruitment strategy 

**Suggestion -** In patients with moderate or severe ARDS, perform MRS as a part of the lung protective strategy to reduce the driving pressure following adjustment of decremental PEEP.^(^ 91 ^,^ 187 ^)^

**Recommendation -** MRS should be performed in PCV mode with a distending pressure of 15cmH_2_O. Start with PEEP=10cmH_2_O and increase PEEP by 5cmH_2_O every two minutes until it reaches 25cmH_2_O; thereafter, PEEP should be increased sequentially by 10cmH_2_O until it reaches 35cmH_2_O, or 45cmH_2_O at most. Next, reduce PEEP to 25cmH_2_O and start decremental PEEP titration (as described in the section above).^(^ 91 ^,^ 187 ^)^

**Recommendation -** Place a central venous access device and perform continuous invasive blood pressure monitoring.^(^ 91 ^,^ 187 ^)^

**Recommendation -** In patients with refractory hypoxemia that does not respond to prone positioning, perform MRS followed by readjustment of PEEP by means of the decremental technique; initiate rescue therapy in eligible patients, with full adherence to the monitoring and safety norms included in the present recommendations. ^(^ 91 ^,^ 187 ^)^

### High-frequency ventilation 

**Recommendation -** Avoid the use of high-frequency ventilation as adjuvant therapy.^(^ 188 ^)^

### Nitric oxide

**Suggestion -** Use inhaled nitric oxide (NO) in patients who have severe ARDS with acute pulmonary hypertension and right ventricular failure; monitor the response and titrate the dose as parts per million (ppm).^(^ 187 ^)^

Extracorporeal membrane oxygenation (venovenous)

**Recommendation -** In patients with refractory hypoxemia, which is defined as a P/F ratio <80mmHg with an FiO_2_>80% after at least three hours of adjuvant and rescue maneuvers for severe ARDS, use veno-venous ECMO when this technology is available. More details are given in the corresponding topic in the present recommendations.^(^ 187 ^)^

## Ventilation in the prone position and extracorporeal circulation

Ventilation in the prone position: when should it be performed?

**Recommendation -** When it is indicated, ventilation in the prone position should be performed during the first 48 hours of MV.^(^ 185 ^,^ 189 ^-^ 191 ^)^

### Indications

**Recommendation -** Avoid routine ventilation in the prone position in mild ARDS.^(^ 185 ^,^ 189 ^-^ 191 ^)^

**Suggestion -** Use ventilation in the prone position in the following situations: after PEEP titration in patients with moderate ARDS,^(^ 185 ^,^ 189 ^-^ 191 ^)^ when there is moderate-to-severe acute right ventricular failure (acute cor pulmonale); when protective ventilation cannot be maintained; or when a distending pressure >15cmH_2_O, a f >35 breaths per minute, and a pH of <7.2 are needed. 

**Recommendation -** Prone positioning should be started early (within 48 hours of the diagnosis of ARDS) in cases of ARDS with a PaO2/FiO2 ratio <150.^(^ 185 ^,^ 189 ^-^ 191 ^)^

How long should prone positioning be maintained? 

**Recommendation -** Maintain prone positioning for a period of 16 to 20 hours, with continuation of all appropriate protective measures and monitoring. ^(^ 185 ^,^ 189 ^-^ 191 ^)^

What are the contraindications to the use of prone positioning?

**Recommendation -** Prone positioning is contraindicated in the following conditions:^(^ 185 ^,^ 189 ^-^ 191 ^)^ intracranial hypertension, pelvic fractures, spine fractures, intra-abdominal hypertension (relative contraindication), laparotomy, pregnancy (relative contraindication), flail chest, severe hemodynamic instability, and inexperienced staff.

Techniques and special care for prone positioning

**Recommendation** - The following techniques and care procedures should be observed:^(^ 185 ^,^ 189 ^-^ 191 ^)^ raise FiO_2_ to 100% while shifting the patient's position; if PCV is used, pay attention to possible reductions in the exhaled volume; sedation and analgesia should be optimized; place a central venous access device and perform continuous invasive blood pressure monitoring; place pillows under the patient, distributed to reduce the pressure on the main anatomical points of support; place cushions below the pelvic and shoulder girdle to reduce abdominal compression; place protective (hydrocolloid) dressings on the patient's forehead, face, knees and shoulders; consider placing an absorbent diaper on the patient's face, and change it whenever it gets too wet; perform electrocardiogram monitoring with the electrodes placed on the patient's back; move the patient, especially his or her head, taking care with anatomical points of support of the face, at least every two hours; change the arm position above and below the interscapular line every two hours at least; maintain enteral nutrition with lower volumes; and check that the patient's eyes are closed. Prone positioning can be maintained for as long as needed, provided that signs of skin or other organ pain attributable to prone positioning do not appear. The response to the shift to prone position should be monitored based on the SpO_2_, if desaturation <90% remains 10 minutes after body rotation, shift to the supine position. The patient should also be shifted to the supine position in case of cardiac arrest, severe hemodynamic aggravation, malignant arrhythmia, or suspected displacement of the ventilatory prosthesis. Repositioning should be performed by three to five persons, and specific training should be provided to the staff, using videos such as those available at the following websites (PROSEVA - three people - http://www.youtube.com/watch?v=E_6jT9R7WJs and William Harvey Hospital - five people - http://www.youtube.com/watch?v=Hd5o4ldp3c0). An arterial blood gas sample should be collected after one hour of prone positioning. A patient should be considered a responder to prone positioning when the pO_2_/FiO_2 _ratio increases by 20 or the PaO_2_ increases by 10mmHg. 

### Extracorporeal gas exchange

**Comment -** Extracorporeal lung assist may in removing CO_2_ or performing extracorporeal membrane oxygenation.^(^ 179 ^,^ 180 ^,^ 192 ^,^ 193 ^)^

### What are the indications of extracorporeal gas exchange?

**Recommendation - **The mandatory criteria for initiation of extracorporeal gas exchange include the following: tracheal intubation and MV; patient ≥18 years old; acute lung disease; reversible lung injury (in some centers in which this technology is available, it is suggested for patients with irreversible lung disease awaiting transplantation); ARDS with PEEP≥10cmH_2_O; and an experienced center. At least one of the following complementary criteria must also be met: hypoxic patients should have a PaO_2_/FiO_2_≤80, with an FiO_2_ of ≥0.8 for at least three hours, despite the performance of rescue maneuvers; hypercapnic patients should have a pH of ≤7.20 with a f = 35 breaths per minute, a Vt of 4 to 6mL/kg predicted body weight, and a mandatory driving pressure of ≤15cmH_2_O.

### What are the contraindications for extracorporeal gas exchange? 

**Recommendation** - Extracorporeal gas exchange is contraindicated in the following situations: dying patients; patients with a body mass index > 40 - 45kg/m^2^; coma (non-sedated patients) after cardiac arrest; patients with irreversible chronic lung disease; lack of accessible and safe vascular access with an appropriate caliber catheter; a life-limiting chronic illness without the perspective of cure; and heparin-induced thrombocytopenia (HIT).^(^ 179 ^,^ 180 ^,^ 192 ^,^ 193 ^)^

What devices are used for extracorporeal gas exchange?

**Suggestion -** Arteriovenous (A-V) interventional lung assist (ILA) is suggested for CO_2_ removal in patients without hemodynamic instability. 

**Suggestion -** Circulatory assistance using ECMO is suggested for oxygenation and CO_2_ removal. 

### Extracorporeal membrane oxygenation - techniques and special care

**Recommendation** - The following techniques and procedures should be used for ECMO. A polymethylpentene membrane should be used for either CO_2_ removal or ECMO. Staff must have extensive experience with the technique (knowledge of the ECMO system and patient physiology, as well as of the most common complications and how to treat them). Improvisation is not acceptable. The venous and percutaneous routes are the first choice for access; cannulas >18 Fr are preferred; if arterial access is needed, and the artery diameter is not >4mm larger than the cannula diameter, seriously consider the use of a distal perfusion cannula before the proximal cannula is placed. Provide safe anticoagulation and monitor the activated partial thromboplastin time (aPTT) and the platelet count every six hours. Initial ventilation should be ultra-protective, using the following settings: controlled ventilation at FiO_2_<0.6; PEEP=10cmH_2_O, distending pressure of 10cmH_2_O and/or Vt <4mL/kg; and f = 10 breaths per minute.^(^ 179 ^,^ 180 ^,^ 192 ^,^ 193 ^)^

**Recommendation -** In venovenous ECMO, maintain ECMO FiO_2_=1 and the lowest possible blood flow rate that is sufficient to maintain the arterial saturation > 90%; maintain the membrane ventilation flow in order to keep the pH at 7.35 - 7.40. 

**Recommendation -** When PSV is used, attempt to achieve the lowest possible work of breathing while preserving patient-ventilation synchrony by using protective ventilation parameters (distending pressure <15cmH_2_O). 

**Recommendation -** In patients with blood flow rate >5,000 - 6,000mL/minute and SaO_2_<85%, consider the following options: increase the ventilator FiO_2_; control agitation; check and correct for recirculation; control the systemic temperature; increase PEEP; induce deep sedation and use neuromuscular blocking agents; perform alveolar recruitment; and consider other options, such as beta-blockers, nitric oxide, prone positioning, and permissive hypoxemia. 

### Interventional lung assistance - techniques and special care

**Recommendation -** The following techniques and procedures should be used for ILA. An echo Doppler should be performed to establish the diameter of the femoral artery and vein, and an ultrasound-guided technique should be used for insertion of the cateters. Cardiac output and perfusion pressure in the system must be ensured (maintain mean arterial pressure >70mmHg), and system flow should be monitored continuously using ultrasound. O_2_ titration should begin at 1L/min and is not to exceed 10L/min. The arterial pH should be monitored in parallel with CO_2_ removal, especially in patients with intracranial hypertension. Protective ventilation should be maintained as was described above for ECMO. Safe anticoagulation should be provided, with monitoring of the aPTT, the fibrinogen level, and the platelet count at least every six hours. Consider removal of cateters under direct visualization (surgical intervention) to reduce vascular complications.^(^ 179 ^,^ 180 ^,^ 192 ^,^ 193 ^)^

## Adjuvant techniques

### Nitric oxide 

**Comment -** The aim of NO use is adjustment of the ventilation/perfusion ratio through vasodilation of the pulmonary artery territory in ventilated areas.

### **Recommendation -** Do not use routinely. 

**Suggestion** - NO may be used when there is acute cor pulmonale, or severe and refractory hypoxemia. 

**Recommendation - **The following techniques and procedures should be employed when NO is used. The NO cylinder should be coupled to its own closed system, with a monitor for inhaled NO and NO_2_. The initial dose should be 5ppm, and NO_2 _should be maintained at <10ppm. Invasive hemodynamic monitoring by means of a thermodilution catheter is preferred. Patients should be monitored for changes in kidney function and methemoglobinemia; do not use NO in patients with methemoglobin reductase deficiency. The patient should not be cared for by pregnant health care providers.^(^ 194 ^)^

### Heliox

**Comment -** The aim of using heliox is to reduce airway resistance and the work of breathing. 

**Suggestion - **Heliox may be used in conditions that are associated with lower airway obstruction to facilitate the maintenance of invasive or noninvasive ventilatory support.^(^ 195 ^,^ 196 ^)^

**Recommendation - **The following techniques and procedures should be employed. Required material should be available (including a ventilator that is prepared for the use of heliox, a heliox regulator, a gas oximeter, and two heliox cylinders, since one needs to be kept as a backup, with an helium/O_2_ concentration not lower than 60/40). Heliox should be discontinued in cases of severe hypoxemia, and intubation should not be delayed, as stated in the recommendations for NIV failure. ^(^ 195 ^)^

### Continuous tracheal gas insufflation 

**Comment -** The aim of continuous tracheal gas insufflation (TGI) is to remove CO_2_ from the gas in the anatomic dead space, thus reducing hypercapnia to a PaCO_2 _of <80mmHg. This resource can be used when Pplat is >30cmH_2_O,Vt is low, and PaCO_2 _is >80mmHg.

**Suggestion -** TGI may be indicated in patients whose f, Cst, and airway pressures are at the respiratory system protection and safety thresholds, but who have a PaCO_2 _of >80mmHg and/or a pH of <7.2.

**Recommendation - **The following techniques and procedures are recommended: capnography with ETCO_2_ measurement should be used, bearing in mind that the efficacy of TGI is greater in patients with high ETCO_2_ levels that are close to the PaCO_2_ level. A bronchoscopy connector should be used for the tracheal cannula, and a fine probe (6 Fr) should be inserted through the connector. The catheter tip should be placed 2 to 3cm above the carina and below the distal end of the ventilatory prosthesis (measured in a tracheal tube outside the trachea). A TGI flow that is sufficient for the expired CO_2_ plateau line (now descending) to come close or contact the zero line should be used. Flow rates >10L/min should be avoided, and TGI should be used in conjunction with PSV, bearing in mind that the volumes measured by the ventilator are inaccurate and that plateau pressure cannot be accurately measured when TGI is used.^(^ 197 ^)^
