# Supplementary material for: Brazilian recommendations of mechanical ventilation 2013. Part I
Source: J Bras Pneumol. 2014 Jul-Aug;40(4):327–63. doi: 10.1590/S1806-37132014000400002 (PMC4201165; doi:10.1590/S1806-37132014000400002)
Supplement: Supplementary file 1 [file 1806-3713-jbpneu-40-04-00327-suppl01.pdf]

Online Suplemment

Brazilian recommendations of mechanical ventilation 2013. Part I

Recomendações brasileiras de ventilação mecânica 2013. Parte I

Workgroup of the Brazilian Association of Intensive Care Medicine and the Brazilian Thoracic Society

Chart 1 - Available ventilators for noninvasive ventilation.

Manufacturer/ Model	Context of use	Available modes	Special modes	Observations
**Philips**				
BIPAP A30	Specific for NIV	AVAPS<br>Auto-trak	Oximetry coupling	Memory card and trends can be coupled
TRILOGY-100	NIV and IMV	PSV, PCV and VCV<br>AVAPS	Leak compensation<br>*Auto-trak*<br>Heated humidification	Monitoring screen<br>6-8 hour battery
**Dixtal**				
DX3012	NIV and IMV	PSV, PCV, VCV,SIMV, CPAP	Leak compensation<br>Active and passive humidification	Monitoring screen, volumetric capnography
**Philips respironics**				
BIPAP-vision, focus and ST	Specific for NIV	BIPAP and CPAP	Auto-trak<br>Leak compensation<br>Slope adjustment<br>FIO_2 control in vision	Monitoring screen
**Resmed**				
Stellar	NIV and IMV	PSV with automatic pressure adjustment<br>iVAPS	FIO_2 control<br>Portable<br>Data *download*	Monitoring screen, preset values for diseases, mask adjustment
**Covidien**				
Covidien 840	NIV module	Spontaneous + PSV<br>A/C and SIMV	Slope and expiratory sensitivity can be adjusted in PSV	Possible *back up* with manual ventilation and/or Vf
**Servo**				
Servo I	NIV module	Spontaneous and PSV	FIO_2 control<br>Adjustment of slope and expiratory cycling	Monitoring screen
**Drager**				
Ventilator Carina	Specific for NIV	VC-SIMV Auto-Flow<br>PC-BIPAP<br>PC-AC<br>SPN-PS (VG)<br>SPN-CPAP<br>Apnea ventilation	1-hour internal battery, 9-hour external battery<br>Automatic triggering and slope control	Monitoring screen and automatic leak compensation
EVITA XL	Special module for NIV	PSV	Automatic adjustments	Monitoring screen
**GE**				
Engstron Pro	VNI and VMI	Multiple modes of ventilation	Internal battery	Monitoring screen and automatic adjustments
**Alliance**				
Care fusion-VELA	Special module for NIV	PSV	6-hour battery<br>Leak compensation	Monitoring screen
**Intermed**				
Care Fusion: IX-5	Special module for NIV	A/C, SIMV, CPAP, PSV	Adjustment of inspiratory and expiratory sensitivity and rise time	Monitoring screen with up to 5 simultaneous curves
Care Fusion: Inter 7 plus	Special module for NIV	A/C, SIMV, CPAP, PSV	Adjustment of inspiratory and expiratory sensitivity and rise time	3-hour internal battery
**VIVO**				
VIVO 40 (pressure up to 40 cmH_2O) and VIVO 30 (pressure up to 30 cmH_2O)	Special module for NIV	PSV, PCV and CPAP	Adjustment of inspiratory and expiratory sensitivity and rise time	External battery<br>Humidification system

Chart 1 - Continued...

Manufacturer/ Model	Context of use	Available modes	Special modes	Observations
**Breas**				
I-sleep 20 (pressure up to 20 cmH$_2$0)	Special module for NIV	CPAP	Adjustable slope<br>Leak compensation	Heated humidifier

BIPAP - bilevel positive air pressure; NIV - noninvasive ventilation; AVAPS - average volume assured pressure support; IMV - invasive mechanical ventilation; PSV - pressure support ventilation mode; VCV - volume controlled ventilation mode; PCV - pressure controlled ventilation mode; SIMV - synchronized inspiratory mandatory ventilation; CPAP - continuous positive air pressure; iVAPS - intelligent volume-assured pressure support; FIO_2 - fraction of inspired oxygen; Vf - respiratory rate; SPN-PS (VG) - spontaneous - pressure support (volume guarantee); SPN - spontaneous - pressure support; A/C - assist/control.

Chart 2 - Basic ventilators (without curve monitoring).

Manufacturer/Model	Age range	Context of use	Available modes	Flow (VCV)	Special modes	Observations
**Air Liquide**						
Taema Osiris	Ad, Ped	Transport	A-C (VCV)	Continuous	- - -	- - -
**Bio-Vent**						
CrossVent CV-3 / CV-4	Ad, Ped	ICU, Transport	A-C (VCV), SIMV, CPAP/ spontaneous, PSV	Continuous	- - -	- - -
**Care Fusion**						
Omni-Tech Omni-Vent	Ad, Ped	Transport	A-C (VCV)		- - -	Can be used in MRI<br>Allows for hyperbaric ventilation
Allied EPV 200	Ad	Transport	A-C (VCV)	Continuous	- - -	- - -
Allied Life Support Autovent 2000, 3000 & 4000	Ad	Transport	A-C (VCV), CPAP/ spontaneous	Continuous	- - -	- - -
**Drager**						
Oxylog 2000 Plus	Ad	Transport	A-C (VCV), SIMV, CPAP/ spontaneous	Continuous	- - -	- - -
Oxylog 3000	Ad, Ped	Transport	A-C (VCV), PCV, SIMV, CPAP/ spontaneous, PSV	Continuous	- - -	- - -
Oxylog 3000	Ad, Ped	Transport	A-C (VCV), PCV, SIMV, CPAP/ spontaneous, PSV	Continuous	- - -	Capnometry.<br>Optional: *autoflow*
**GE**					- - -	
Bi-Level 40	Ad, Ped	Transport	A-C (VCV), PCV, SIMV, CPAP/ spontaneous, PSV	Continuous	- - -	- - -
**K. Takaoka**						
Mini-ventil 600	Ad	Transport	A-C (VCV)	Continuous	- - -	- - -
MicroTak 920	Ad, Ped	Transport	A-C (VCV), SIMV, CPAP/ spontaneous	Continuous	- - -	- - -
**Leistung**						
PR 4D-02	Ad, Ped	Transport	A-C (VCV)	Continuous	- - -	Time-cycled
**Res Med**						
VS III	Ad, Ped	ICU, NIV	A-C (VCV), PCV, SIMV, CPAP/ spontaneous, PSV	Continuous	- - -	- - -
**Tyco / Covidien**						
Newport HT 70	Ad, Ped	Transport	A-C (VCV), PCV, SIMV, CPAP/ spontaneous, PSV	Continuous<br>Decelerating	Rise time<br>% expiratory time-PSV	- - -
**Vent-Logos**						
VLP 2000 – E	Ad	Transport	A-C (VCV)	Continuous	- - -	- - -
VLP 4000 – P	Ad	Transport	A-C (VCV)	Continuous	- - -	- - -

Ad - use in adults; Ped - use in children (non-newborn); A-C - assist control; VCV - volume controlled ventilation mode; SIMV - synchronized inspiratory mandatory ventilation; CPAP - continuous positive air pressure; PCV - pressure controlled ventilation mode; PSV - pressure support ventilation mode; MRI - magnetic resonance imaging; transport - use for transportation of patients under invasive mechanical ventilation; ICU - appropriate for use in intensive care unit; NIV - use for noninvasive ventilation.

Chart 3 - Ventilators with basic resources and with curves.

Manufacturer/ Model	Age range	Context of use	Available modes	Flow (VCV)	Monitoring	Special modes	Observation
**Air Liquide**							
Extend XT	Ad, Neo	ICU	A-C (VCV), PCV, SIMV, CPAP/spontaneous, PSV	Continuous, decelerating	Curves, loops	- - -	Capnography
**Care Fusion**							
Intermed Inter-5 Plus	Ad, Ped	ICU	A-C (VCV), PCV, SIMV, CPAP/spontaneous, PSV	Continuous, decelerating	Curves	- - -	Separate monitor
**Drager**							
Savina 300	Ad, Ped	ICU	A-C (VCV), PCV, SIMV, CPAP/spontaneous, PSV	Continuous, decelerating	Curves	BiLevel	*Auto-Flow*
**GE**							
Ventil Pulmonar 101	Ad, Ped	ICU, home	A-C (VCV), PCV, SIMV, CPAP/spontaneous, PSV	Continuous, decelerating	Curves	PRVC, volume guaranteed	iVent MRI: can be used in MRI
**Hamilton**							
Galileo Gold	Ad, Ped	ICU	A-C (VCV), PCV, SIMV, CPAP/spontaneous, PSV	Continuous, decelerating	Curves, loops	ASV, APRV, automatic tube compensation	Automatic P-V curve, P0.1
Raphael Color	Ad, Ped	ICU, ER	A-C (VCV), PCV, SIMV, CPAP/spontaneous, PSV	Continuous, decelerating	Curves, loops	ASV, APRV, automatic tube compensation	- - -
T-1	Ad, Ped	Transport	A-C (VCV), PCV, SIMV, CPAP/spontaneous, PSV	Continuous, decelerating	Curves	ASV	Automatic P-V curve Capnometry Barometric pressure compensation (air transportation)
MR-1	Ad, Ped	Transport	A-C (VCV), PCV, SIMV, CPAP/spontaneous, PSV	Continuous, decelerating	Curves	ASV	For MRI "Extra" monitor
**K-Takaoka**							
Smart	Ad, Neo	ICU, ER	A-C (VCV), PCV, SIMV, CPAP/spontaneous, PSV	Continuous, decelerating	Curves, loops	BiLevel, orotracheal tube compensation	P0.1 PiMax
Carmel	Ad, Neo	ICU	A-C (VCV), PCV, SIMV, CPAP/spontaneous, PSV	Continuous, decelerating	Curves	PSV: RiseTime & time adjustment (% flow), PCV volume guaranteed	Capnometry
**Leistung**							
Luft 1-g	Ad, Ped	ICU, ER	A-C (VCV), PCV, SIMV, CPAP/spontaneous, PSV	Continuous, decelerating	Curves	- - -	- - -
Luft 2-g	Ad, Ped	ICU	A-C (VCV), PCV, SIMV, CPAP/spontaneous, PSV	Continuous, decelerating	Curves, loops	MMV, BiLevel, PSV volume guaranteed, APRV	- - -
PR - 4g	Ad, Ped	Transport	A-C (VCV), PCV, SIMV, CPAP/spontaneous, PSV	Continuous, decelerating	Curves	- - -	- - -
**Magnamed**							
Fleximag	Ad, Neo	ICU, ER	A-C (VCV), PCV, SIMV, CPAP/spontaneous, PSV	Continuous, decelerating	Curves	BiLevel, PSV: rise time	Capnography (optional)
Oxymag	Ad, Neo	Transport	A-C (VCV), PCV, SIMV, CPAP/spontaneous, PSV	Continuous, decelerating	Curves	BiLevel, APRV	Capnography (optional)
**Neumovent**							
GraphNet T5	Ad, Ped	ICU, ER	A-C (VCV), PCV, SIMV, CPAP/spontaneous, PSV	Continuous, decelerating	Curves	- - -	- - -
**Tyco/Covidien**							
Newport e360	Ad	ICU, ER	A-C (VCV), PCV, SIMV, CPAP/spontaneous, PSV	Continuous, decelerating	Curves, loops	BiLevel, PSV: rise time & time adjustment (% flow), APRV	- - -

Ad - use in adults; Ped - use in children (non-newborn); Neo - use in neonatology; ICU - appropriate for use in ICU; PSV - pressure support ventilation mode; VCV - volume controlled ventilation mode; PCV - pressure controlled ventilation mode; SIMV - synchronized inspiratory mandatory ventilation; CPAP - continuous positive air pressure; APRV - airway pressure release ventilation; PRVC - pressure regulated volume controlled; ASV - adaptative support ventilation; MMV - minute mandatory ventilation; ER - use in emergency room.

Chart 4 - Ventilators with curve monitoring and advanced resources.

Manufacturer/ Model	Age range	Context of use	Basic modes	Flow (VCV)	Monitoring	Special modes	Observations
**Air Liquide**							
Monnal T-75	Ad, Neo	ICU	Yes	Continuous, decelerating	Curves, loops	BiLevel CPAP, PRVC	Capnography
**Care Fusion**							
Viasys Vela	Ad, Ped	ICU	Yes	Continuous, decelerating	Curves, loops	PSV: RiseTime & time adjustment (% flow), PRVC, APRV	Capnography
Viasys Avea	Ad, Neo	ICU	Yes	Continuous, decelerating	Curves, loops	V-PSV, PSV: RiseTime & time adjustment (% flow), Vsync, PRVC, AAC (tube compensation)	Capnography Measures P0.1, Pimax, WOB (esophageal)
Intermed i X5	Ad, Neo	ICU	Yes	Continuous, decelerating	Curves, loops	PSV: RiseTime & time adjustment (% flow), TGI, tube compensation	Measures P0.1
Intermed Inter-7 Plus	Ad, Neo	ICU	Yes	Continuous, decelerating	Curves, loops	PSV: RiseTime & time adjustment (% flow), VAPS, BiLevel, TGI, APRV (BiPEEP)	Measures P0.1
**Drager**							
Evita 4	Ad, Ped	ICU	Yes	Continuous, decelerating	Curves, loops	MMV, Auto-Flow, APRV, PPS (optional), ATC – tube compensation	Capnography, PiMax, Vd/Vt
Evita XL	Ad, Neo	ICU	Yes	Continuous, decelerating	Curves, loops	Auto-Flow, automatic tube compensation, Smart Care Variable SP, BiLevel, PC-APRV, Recruitment maneuver, Low flow maneuver	Capnography, oximetry, Lung protection package (optional)
**GE**							
Engstron Carestation	Ad, Neo	ICU	Yes	Continuous, decelerating	Curves, loops	BiLevel PSV: RiseTime & time adjustment (% flow), Automatic tube compensation, APRV, PC-volume guaranteed	P0.1, PiMax, Measures FRC, indirect calorimetry
Engstron Pro	Ad, Neo	ICU	Yes	Continuous, decelerating	Curves, loops	BiLevel, PSV: RiseTime & time adjustment (% flow), automatic orotracheal tube compensation, APRV, PC-volume guaranteed	P0.1
**Hamilton**							
C-3	Ad, Ped	ICU, ER	Yes	Continuous, decelerating	Curves	ASV, APRV	Volumetric capnometry (optional),
C-2	Ad, Ped	ICU, ER	Yes	Continuous, decelerating	Curves, loops	ASV, APV, Tube compensation, APRV	Automatic P-V curve, Volumetric capnometry (optional),
S-1	Ad, Ped	ICU	Yes	Continuous, decelerating	Curves, loops	ASV, APRV	Automatic P-V curve, capnography,
G-5	Ad, Neo	ICU	Yes	Continuous, decelerating	Curves, loops	ASV, BiLevel, APRV, APV	Allows heliox (optional), Volumetric capnography (optional),
**K Takaoka**							
Color	Ad, Neo	ICU	Yes	Continuous, decelerating	Curves	MMV, PSV-volume guaranteed, BiLevel, tube compensation	P0.1, PiMax, Capnometry
**Maquet Getinge**							
Servo-i	Ad, Ped	ICU	Yes	Continuous, decelerating	Curves, loops	NAVA (optional), AutoMode, BiLevel, PSV: RiseTime & time adjustment (% flow), PRVC, APRV	Capnography, P0.1, WOB (*in-line*)

Chart 4 - Continued...

Manufacturer/ Model	Age range	Context of use	Basic modes	Flow (VCV)	Monitoring	Special modes	Observations
Servo-S	Ad, Ped	ICU	Yes	Continuous, decelerating	Curves, loops	BiLevel, PSV: RiseTime & time adjustment (% flow), PRVC, APRV	P0.1
**Mindray**							
Synovent E3	Ad, Ped	Hospital, ICU, ER	Yes	Continuous, decelerating	Curves, loops	Bilevel, PSV, optional automatic expiratory trigger control, rise time, time adjustment, tube compensation	Capnography, P0.1, WOB, PiMax, enables connectivity with the hospital system (Bnelink/HL7)
Synovent E5	Ad, Ped	ICU	Yes	Continuous, decelerating	Curves, loops	PRVC, APRV, Bilevel, PSV, optional automatic expiratory trigger control, rise time, time adjustment, tube compensation	Capnography, P0.1, WOB, PiMax, low flow maneuver, enables connectivity with the hospital system (Bnelink/HL7), displays 4 simultaneous curves (P/T, V/T, F/T and capnography)
**Neumovent**							
GraphNet Advance	Ad, Neo	ICU	Yes	Continuous, decelerating	Curves, loops	PRVC, APRV	Capnography
**Philips**							
Dixtal DX-3012 Plus	Ad, Ped	ICU	Yes	Continuous, decelerating	Curves, loops	BiLevel, PS-volume guaranteed, MMV, PSV: rise time & time adjustment (% flow), APRV	P0.1, Capnography
Dixtal DX-3012	Ad, Ped	ICU	Yes	Continuous, decelerating	Curves, loops	BiLevel, PS-Vol Gar, MMV, PSV: rise time & time adjustment (% flow), APRV	Capnography, P0.1, PiMax
**Tyco/Covidien**							
Puritan Bennett 840	Ad, Ped	ICU	Yes	Continuous, decelerating	Curves, loops	BiLevel, PSV: rise time & time adjustment (% flow), PAV-Plus, automatic tube compensation, APRV	

Ad - use in adults; Ped - use in children (non-newborn); Neo - use in neonatology; ICU - appropriate for use in ICU; PSV - pressure support ventilation mode; Bipap - bilevel positive air pressure; VCV - volume controlled ventilation mode; PCV - pressure controlled ventilation mode; SIMV - synchronized inspiratory mandatory ventilation; CPAP - continuous positive air pressure; iVAPS - intelligent volume-assured pressure support; APRV - airway pressure release ventilation; PRVC - pressure regulated volume controlled; ASV - adaptative support ventilation; MMV - minute mandatory ventilation; ER - use in emergency room; VAPS - volume assured pressure support ventilation mode; Pimax - maximal inspiratory pressure; FRC - functional residual capacity; NAVA - neurally adjust ventilatory assist; PAV - proportional assist ventilation; APV - adaptative pressure ventilation; PS - pressure support.

Chart 5 - Mode-dedicated ventilators HFOV (adults).

Manufacturer/Model	Age range
**Care Fusion**	
SensorMedics 3100 B	Ad, Ped

HFOV - high frequency oscillatory ventilation; Ad - use in adults; Ped - use in children (non-newborn).
