# Supplementary material for: Brazilian recommendations of mechanical ventilation 2013. Part I
Source: J Bras Pneumol. 2014 Jul-Aug;40(4):327–63. doi: 10.1590/S1806-37132014000400002 (PMC4201165; doi:10.1590/S1806-37132014000400002)
Supplement: Supplementary file 2 [file 1806-3713-jbpneu-40-04-00327-suppl01-pt.pdf]

Recomendações brasileiras de ventilação mecânica 2013. Parte I

Brazilian recommendations of mechanical ventilation 2013. Part I

Grupo de trabalho AMIB e SBPT

Quadro 1 - Ventiladores disponíveis para realização de ventilação não invasiva.

Fabricante/ Modelo	Contexto de utilização	Modos disponíveis	Modos especiais	Observações
**Philips**				
BIPAP A30	Especial para VNI	AVAPS<br>*Auto-trak*	Acoplamento de oximetria	Pode acoplar cartão de memória e tendências
TRILOGY-100	VNI e VMI	PSV, PCV e VCV<br>AVAPS	Compensação de fugas<br>*Auto-trak*<br>Umidificação aquecida	Tela de monitorização<br>Bateria de 6 a 8 horas
**Dixtal**				
DX3012	VNI e VMI	PSV, PCV, VCV,SIMV,CPAP	Compensação de fugas<br>Umidificação ativa e passiva	Tela de monitorização, capnografia volumétrica
**Philips respironics**				
BIPAP-vision, focus e ST	Especiais para não invasiva	BIPAP e CPAP	*Auto-trak*<br>Compensação de fugas<br>Ajuste de rampa<br>Controle de FIO_2 *no vision*	Tela de monitorização
**Resmed**				
Stellar	VNI e VMI	PSV com ajuste automático de pressão<br>iVAPS	Controle de FIO_2<br>Portátil<br>*Download* de dados	Tela de monitorização, valores predefinidos para doenças, ajuste de máscaras
**Covidien**				
Covidien 840	Módulo de VNI	Espontâneo + PSV<br>A/C e SIMV	Possibilidade de ajuste de rampa e sensibilidade expiratória na PSV	Possibilidade de *back up* com ventilação manual e ou f
**Servo**				
Servo I	Módulo de VNI	Espontâneo e PSV	Controle de FIO_2<br>Ajuste de rampa e ciclagem expiratória	Tela de monitorização
**Drager**				
Ventilador Carina	Especial para VNI	VC-SIMV *Auto-Flow*<br>PC-BIPAP<br>PC-AC<br>SPN-PS (VG)<br>SPN-CPAP<br>Ventilação de apneia	Bateria interna de 1 hora e externa de 9 horas<br>Disparo e controle de rampa automáticos	Tela de monitorização e compensação automática de fugas
EVITA XL	Modulo especial para não invasiva	PSV	Ajustes automáticos	Tela de monitorização
**GE**				
Engstron Pro	VNI e VMI	Multiplos modos ventilatórios	Bateria interna	Tela de monitorização e ajustes automáticos
**Ventilador Alliance**				
Care fusion-VELA	Modulo específico para não invasiva	PSV	Bateria para 6 horas<br>Compensação de vazamentos	Tela de monitorização
**Intermed**				
Care Fusion: IX-5	Modulo específico para não invasiva	A/C, SIMV, CPAP, PSV	Ajuste de sensibilidade inspiratória, expiratória e *rise time*	Tela de monitorização com até 5 curvas simultâneas
Care Fusion: Inter 7 plus	Modulo específico para não invasiva	A/C, SIMV, CPAP, PSV	Ajuste de sensibilidade inspiratória, expiratória e *rise time*	Bateria interna com até 3 horas
**Ventilador - VIVO**				
VIVO 40 (pressão até 40cmH_2O) e VIVO 30 (pressões até 30cmH_2O)	Especial para não invasiva	PSV, PCV e CPAP	Ajuste de sensibilidade inspiratória e expiratória, ajuste de *rise time*	Bateria externa<br>Sistema de umidificação

Quadro 1 - Continuação...

Fabricante/ Modelo	Contexto de utilização	Modos disponíveis	Modos especiais	Observações
**Breas**				
I-sleep 20 (pressões até 20cm$H_2$0)	Especial para não invasiva	CPAP	Rampa ajustável<br>Compensação de vazamentos	Umidificador aquecido

BIPAP - *bilevel positive air pressure*; VNI - ventilação não invasiva; AVAPS - *average volume assured pressure support*; VMI - ventilação mecânica invasiva; PSV - *pressure support ventilation mode*; VCV - *volume controlled ventilation mode*; PCV - *pressure controlled ventilation mode*; SIMV - *synchronized inspiratory mandatory ventilation*; CPAP - *continuous positive air pressure*; iVAPS - *intelligent volume-assured pressure support*; FIO_2 - fração inspirada de oxigênio; f - frequencia respiratória; SPN-PS (VG) - *spontaneous - pressure support (volume guarantee)*; SPN - *spontaneous - pressure support*; A/C - assistido/controlado.

Quadro 2 - Ventiladores básicos (sem monitorização de curvas).

Fabricante/Modelo	Faixa etária	Contexto de utilização	Modos disponíveis	Fluxo (VCV)	Modos especiais	Observações
**Air Liquide**						
Taema Osiris	Ad, Ped	Transporte	A-C (VCV)	Contínuo	- - -	- - -
**Bio-Ventw**						
CrossVent CV-3 / CV-4	Ad, Ped	UTI, Transporte	A-C (VCV), SIMV, CPAP/ espontâneo, PSV	Contínuo	- - -	- - -
**Care Fusion**						
Omni-Tech Omni-Vent	Ad, Ped	Transporte	A-C (VCV)		- - -	Permite uso em RNM<br>Permite ventilação hiperbárica
Allied EPV 200	Ad	Transporte	A-C (VCV)	Contínuo	- - -	- - -
Allied Life Support Autovent 2000, 3000 & 4000	Ad	Transporte	A-C (VCV), CPAP/espontâneo	Contínuo	- - -	- - -
**Drager**						
Oxylog 2000 Plus	Ad	Transporte	A-C (VCV), SIMV, CPAP/ espontâneo	Contínuo	- - -	- - -
Oxylog 3000	Ad, Ped	Transporte	A-C (VCV), PCV, SIMV, CPAP/ espontâneo, PSV	Contínuo	- - -	- - -
Oxylog 3000	Ad, Ped	Transporte	A-C (VCV), PCV, SIMV, CPAP/ espontâneo, PSV	Contínuo	- - -	Capnometria. Opcional: *autoflow*
**GE**					- - -	
Bi-Level 40	Ad, Ped	Transporte	A-C (VCV), PCV, SIMV, CPAP/ espontâneo, PSV	Contínuo	- - -	- - -
**K. Takaoka**						
Mini-ventil 600	Ad	Transporte	A-C (VCV)	Contínuo	- - -	- - -
MicroTak 920	Ad, Ped	Transporte	A-C (VCV), SIMV, CPAP/ espontâneo	Contínuo	- - -	- - -
**Leistung**						
PR 4D-02	Ad, Ped	Transporte	A-C (VCV)	Contínuo	- - -	Ciclado a tempo
**Res Med**						
VS III	Ad, Ped	UTI, VNI	A-C (VCV), PCV, SIMV, CPAP/ espontâneo, PSV	Contínuo	- - -	- - -
**Tyco / Covidien**						
Newport HT 70	Ad, Ped	Transporte	A-C (VCV), PCV, SIMV, CPAP/ espontâneo, PSV	Contínuo, Desacelerado	*Rise time*<br>% tempo expiratório-PSV	- - -
**Vent-Logos**						
VLP 2000 – E	Ad	Transporte	A-C (VCV)	Contínuo	- - -	- - -
VLP 4000 – P	Ad	Transporte	A-C (VCV)	Contínuo	- - -	- - -

Ad - uso em adultos; Ped - uso em crianças (não neonatos); A-C - assistido controlado; VCV - *volume controlled ventilation mode;* SIMV - *synchronized inspiratory mandatory ventilation*; CPAP - *continuous positive air pressure;* PCV - *pressure controlled ventilation mode*; PSV - *pressure support ventilation mode;* RNM - ressonância nuclear magnética; transporte - uso em transporte do paciente sob ventilação mecânica invasiva; UTI - uso apropriado para unidade de terapia intensiva; VNI - uso para ventilação não invasiva.

 http://dx.doi.org/10.1590/S1806-37132014000400002

Quadro 3 - Ventiladores com recursos básicos, com curvas.

Fabricante/ Modelo	Faixa etária	Contexto de utilização	Modos disponíveis	Fluxo (VCV)	Monitoramento	Modos especiais	Observações
**Air Liquide**							
Extend XT	Ad, Neo	UTI	A-C (VCV), PCV, SIMV, CPAP/Espontâneo, PSV	Contínuo, Desacelerado	Curvas, Alças	- - -	Capnógrafo
**Care Fusion**							
Intermed Inter-5 Plus	Ad, Ped	UTI	A-C (VCV), PCV, SIMV, CPAP/Espontâneo, PSV	Contínuo, Desacelerado	Curvas	- - -	Monitor em separado
**Drager**							
Savina 300	Ad, Ped	UTI	A-C (VCV), PCV, SIMV, CPAP/Espontâneo, PSV	Contínuo, Desacelerado	Curvas	BiLevel	*Auto-Flow*
**GE**							
Ventil Pulmonar 101	Ad, Ped	UTI, Domicílio	A-C (VCV), PCV, SIMV, CPAP/Espontâneo, PSV	Contínuo, Desacelerado	Curvas	PRVC, Vol garantido	iVent MRI: pode ser utilizado para RNM
**Hamilton**							
Galileo Gold	Ad, Ped	UTI	A-C (VCV), PCV, SIMV, CPAP/Espontâneo, PSV	Contínuo, Desacelerado	Curvas, Alças	ASV, APRV, Compensação automática tubo	Curva P-V automática, P0.1
Raphael Color	Ad, Ped	UTI, PS	A-C (VCV), PCV, SIMV, CPAP/Espontâneo, PSV	Contínuo, Desacelerado	Curvas, Alças	ASV, APRV, Compensação automática tubo	- - -
T-1	Ad, Ped	Transporte	A-C (VCV), PCV, SIMV, CPAP/Espontâneo, PSV	Contínuo, Desacelerado	Curvas	ASV	Curva P-V automática Capnometria Compensação de pressão barométrica (transporte aéreo)
MR-1	Ad, Ped	Transporte	A-C (VCV), PCV, SIMV, CPAP/Espontâneo, PSV	Contínuo, Desacelerado	Curvas	ASV	Para RNM Monitor "extra"
**K-Takaoka**							
Smart	Ad, Neo	UTI, PS	A-C (VCV), PCV, SIMV, CPAP/Espontâneo, PSV	Contínuo, Desacelerado	Curvas, Alças	BiLevel, Compens TOT	P0.1 PiMax
Carmel	Ad, Neo	UTI	A-C (VCV), PCV, SIMV, CPAP/Espontâneo, PSV	Contínuo, Desacelerado	Curvas	PSV: *RiseTime* & Ajuste de tempo (%Fluxo), PCV-volume garantido	Capnometria
**Leistung**							
Luft 1-g	Ad, Ped	UTI, PS	A-C (VCV), PCV, SIMV, CPAP/Espontâneo, PSV	Contínuo, Desacelerado	Curvas	- - -	- - -
Luft 2-g	Ad, Ped	UTI	A-C (VCV), PCV, SIMV, CPAP/Espontâneo, PSV	Contínuo, Desacelerado	Curvas, Alças	MMV, BiLevel, PSV-volume garantido, APRV	- - -
PR - 4g	Ad, Ped	Transporte	A-C (VCV), PCV, SIMV, CPAP/Espontâneo, PSV	Contínuo, Desacelerado	Curvas	- - -	- - -
**Magnamed**							
Fleximag	Ad, Neo	UTI, PS	A-C (VCV), PCV, SIMV, CPAP/Espontâneo, PSV	Contínuo, Desacelerado	Curvas	BiLevel, PSV: *rise time*	Capnógrafo (opcional)
Oxymag	Ad, Neo	Transporte	A-C (VCV), PCV, SIMV, CPAP/Espontâneo, PSV	Contínuo, Desacelerado	Curvas	BiLevel, APRV	Capnógrafo (opcional)
**Neumovent**							
GraphNet T5	Ad, Ped	UTI, PS	A-C (VCV), PCV, SIMV, CPAP/Espontâneo, PSV	Contínuo, Desacelerado	Curvas	- - -	- - -
**Tyco/Covidien**							
Newport e360	Ad	UTI, PS	A-C (VCV), PCV, SIMV, CPAP/Espontâneo, PSV	Contínuo, Desacelerado	Curvas, Alças	BiLevel, PSV: *rise time* & Ajuste tempo (%Fluxo), APRV	- - -

Ad - uso em adultos; Ped - uso em crianças (não neonatos); Neo - uso em neonatologia; UTI = uso apropriado para UTI; PSV - *pressure support ventilation mode*; VCV - *volume controlled ventilation mode*; PCV - *pressure controlled ventilation mode*; SIMV - *synchronized inspiratory mandatory ventilation*; CPAP - *continuous positive air pressure*; APRV - *airway pressure release ventilation*; PRVC - *pressure regulated volume controlled*; ASV - *adaptative support ventilation*; MMV - *minute mandatory ventilation*; PS - uso no pronto-socorro.

Quadro 4 - Ventiladores com monitorização de curvas e recursos avançados.

Fabricante/ Modelo	Faixa etária	Contexto de utilização	Modos básicos	Fluxo (VCV)	Monitoramento	Modos especiais	Observações
**Air Liquide**							
Monnal T-75	Ad, Neo	UTI	Sim	Contínuo, Desacelerado	Curvas, Alças	BiLevel CPAP, PRVC	Possui capnógrafo
**Care Fusion**							
Viasys Vela	Ad, Ped	UTI	Sim	Contínuo, Desacelerado	Curvas, Alças	PSV: *rise time* & ajuste tempo (% Fluxo), PRVC, APRV	Capnógrafo
Viasys Avea	Ad, Neo	UTI	Sim	Contínuo, Desacelerado	Curvas, Alças	V-PSV, PSV: *RiseTime* & Ajuste tempo (% Fluxo), Vsinc, PRVC, AAC (Compens tubo)	Capnógrafo Mede P0.1, Pimax, WOB (esofágica)
Intermed i X5	Ad, Neo	UTI	Sim	Contínuo, Desacelerado	Curvas, Alças	PSV: *RiseTime* & Ajuste tempo (% Fluxo), TGI, Compens tubo	Mede P0.1
Intermed Inter-7 Plus	Ad, Neo	UTI	Sim	Contínuo, Desacelerado	Curvas, Alças	PSV: *RiseTime* & Ajuste tempo (% Fluxo), VAPS, BiLevel, TGI, APRV (BiPEEP)	Mede P0.1
**Drager**							
Evita 4	Ad, Ped	UTI	Sim	Contínuo, Desacelerado	Curvas, Alças	MMV, *Auto-Flow*, APRV, PPS (opcional), ATC - Compens tubo	Capnógrafo, PiMax, Vd/Vt
Evita XL	Ad, Neo	UTI	Sim	Contínuo, Desacelerado	Curvas, Alças	*Auto-Flow*, Compensação automatica de tubo, *Smart Care* PS variável, BiLevel, PC-APRV, Manobra recrutamento, Manobra fluxo baixo	Capnógrafo, Oxímetro, *Lung protection package* (opcional)
**GE**							
Engstron Carestation	Ad, Neo	UTI	Sim	Contínuo, Desacelerado	Curvas, Alças	BiLevel PSV: *RiseTime* & Ajuste tempo (% Fluxo), Compensação automática de tubo, APRV, PC-Volume garantido	P0.1, PiMax, Mede CRF, Calorimentria indireta
Engstron Pro	Ad, Neo	UTI	Sim	Contínuo, Desacelerado	Curvas, Alças	BiLevel, PSV: *RiseTime* & Ajuste tempo (% Fluxo), Compensação automática de TOT, APRV, PC-volume garantido	P0.1
**Hamilton**							
C-3	Ad, Ped	UTI, PS	Sim	Contínuo, Desacelerado	Curvas	ASV, APRV	Capnometria volumétrica (opcional),
C-2	Ad, Ped	UTI, PS	Sim	Contínuo, Desacelerado	Curvas, Alças	ASV, APV, Compensação do tubo, APRV	Curva P-V automática, Capnografia volumétrica (opcional),
S-1	Ad, Ped	UTI	Sim	Contínuo, Desacelerado	Curvas, Alças	ASV, APRV	Curva P-V automática, Capnógrafo,
G-5	Ad, Neo	UTI	Sim	Contínuo, Desacelerado	Curvas, Alças	ASV, BiLevel, APRV, APV	Permilte Heliox (opcional), Capnografia volumétrica (opcional)
**K Takaoka**							
Color	Ad, Neo	UTI	Sim	Contínuo, Desacelerado	Curvas	MMV, PSV-Volume garantido, BiLevel, Compensação do tubo	P0.1, PiMax, Capnometria

Quadro 4 - Continuação...

Fabricante/ Modelo	Faixa etária	Contexto de utilização	Modos básicos	Fluxo (VCV)	Monitoramento	Modos especiais	Observações
**Maquet Getinge**							
Servo-i	Ad, Ped	UTI	Sim	Contínuo, Desacelerado	Curvas, Alças	NAVA (opcional), AutoMode, BiLevel, PSV: *RiseTime* & Ajuste tempo (% Fluxo), PRVC, APRV	Capnógrafo, P0.1, WOB (*in-line*)
Servo-S	Ad, Ped	UTI	Sim	Contínuo, Desacelerado	Curvas, Alças	BiLevel, PSV: *RiseTime* & Ajuste tempo (% Fluxo), PRVC, APRV	P0.1
**Mindray**							
Synovent E3	Ad, Ped	Hospital, UTI, PS	Sim	Contínuo, Desacelerado	Curvas, Alças	Bilevel, PSV, opção de controle de trigger expiratório automático, *rise time*, ajuste de tempo, compensação de tubo	Capnógrafo, P0.1, Trabalho respiratório, PiMax, permite conectividade com sistema hospitalar (Bnelink/HL7)
Synovent E5	Ad, Ped	UTI	Sim	Contínuo, Desacelerado	Curvas, Alças	PRVC, APRV, Bilevel, PSV, opção de controle de trigger expiratório automático, *rise time*, ajuste de tempo, compensação de tubo	Capnógrafo, P0.1, Trabalho respiratório, PiMax, manobra de baixo fluxo permite conectividade com sistema hospitalar (Bnelink/HL7), exibe quatro curvas simultâneas (PxT, VxT, FxT e capnografia)
**Neumovent**							
GraphNet Advance	Ad, Neo	UTI	Sim	Contínuo, Desacelerado	Curvas, Alças	PRVC, APRV	Capnógrafo
**Philips**							
Dixtal DX-3012 Plus	Ad, Ped	UTI	Sim	Contínuo, Desacelerado	Curvas, Alças	BiLevel, PS-Vol Gar, MMV, PSV: *rise time* & ajuste tempo (% fluxo), APRV	P0.1, Capnógrafo
Dixtal DX-3012	Ad, Ped	UTI	Sim	Contínuo, Desacelerado	Curvas, Alças	BiLevel, PS-Vol Gar, MMV, PSV: *rise time* & ajuste tempo (% fluxo), APRV	Capnógrafo, P0.1, PiMax
**Tyco/Covidien**							
Puritan Bennett 840	Ad, Ped	UTI	Sim	Contínuo, Desacelerado	Curvas, Alças	BiLevel, PSV: *rise time* & ajuste tempo (% fluxo), PAV-*Plus*, compensação automática de tubo, APRV	

Ad - uso em adultos; Ped - uso em crianças (não neonatos); Neo - uso em neonatologia; UTI - uso apropriado para UTI; PSV - *pressure support ventilation mode*; Bipap - *bilevel positive air pressure*; VCV - *volume controlled ventilation mode*; PCV - *pressure controlled ventilation mode*; SIMV - *synchronized inspiratory mandatory ventilation*; CPAP - *continuous positive air pressure*; iVAPS - *intelligent volume-assured pressure support*; APRV - *airway pressure release ventilation*; PRVC - *pressure regulated volume controlled*; ASV - *adaptative support ventilation*; MMV - *minute mandatory ventilation*; PS - uso no pronto-socorro; VAPS - *volume assured pressure support ventilation mode*; Pimax - pressão inspiratória máxima; CRF - capacidade residiual funcional; NAVA - neurally adjust ventilatory assist; PAV - *proportional assist ventilation*; APV - adaptative pressure ventilation; PS-Vol Gar - *pressure support* (*volume guarantee*).

Quadro 5 - Ventiladores modo-dedicados HFOV (adultos).

Fabricante/Modelo	Faixa etária
**Care Fusion**	
SensorMedics 3100 B	Ad, Ped

HFOV - *high frequency oscillatory ventilation*; Ad - uso em adultos; Ped - uso em crianças (não neonatos).
