# Supplementary material for: Brazilian recommendations of mechanical ventilation 2013. Part I
Source: J Bras Pneumol. 2014 Jul-Aug;40(4):327–63. doi: 10.1590/S1806-37132014000400002 (PMC4201165; doi:10.1590/S1806-37132014000400002)
Supplement: Translation

# Translation: Recomendações brasileiras de ventilação mecânica 2013. Parte I

**Keywords:** Recomendações, Ventilação Mecânica, Insuficiência Respiratória

## Abstract

O suporte ventilatório artificial invasivo e não invasivo ao paciente crítico tem evoluído e inúmeras evidências têm surgido, podendo ter impacto na melhora da sobrevida e da qualidade do atendimento oferecido nas unidades de terapia intensiva no Brasil. Isto posto, a Associação de Medicina Intensiva Brasileira (AMIB) e a Sociedade Brasileira de Pneumologia e Tisiologia (SBPT) - representadas pelo seus Comitê de Ventilação Mecânica e Comissão de Terapia Intensiva, respectivamente, decidiram revisar a literatura e preparar recomendações sobre ventilação mecânica objetivando oferecer aos associados um documento orientador das melhores práticas da ventilação mecânica na beira do leito, baseado nas evidencias existentes, sobre os 29 subtemas selecionados como mais relevantes no assunto. O projeto envolveu etapas visando distribuir os subtemas relevantes ao assunto entre experts indicados por ambas as sociedades que tivessem publicações recentes no assunto e/ou atividades relevantes em ensino e pesquisa no Brasil na área de ventilação mecânica. Esses profissionais, divididos por subtemas em duplas, responsabilizaram-se por fazer revisão extensa da literatura mundial sobre cada subtema. Reuniram-se todos no Forum de Ventilação Mecânica na sede da AMIB em São Paulo, em 03 e 04 de agosto de 2013 para finalização conjunta do texto de cada subtema e apresentação, apreciação, discussão e aprovação em plenária pelos 58 participantes, permitindo a elaboração de um documento final.

Essas recomendações são uma iniciativa conjunta do Comitê de Ventilação Mecânica da Associação de Medicina Intensiva Brasileira (AMIB) e da Comissão de Terapia Intensiva da Sociedade Brasileira de Pneumologia e Tisiologia (SBPT).

## Introdução

O suporte ventilatório mecânico, tanto não invasivo como invasivo, deve ser realizado de forma adequada e segura para evitarmos a lesão induzida pela ventilação mecânica. A ventilação mecânica moderna e atual, guiada pelos conhecimentos de fisiologia e as evidências literárias tanto dos experimentos de laboratório, como pelos ensaios clínicos randomizados e/ou observacionais com pacientes, nos indicam um suporte ventilatório com volumes correntes de 6mL/Kg de peso predito, delta entre a pressão de platô e a pressão expiratória final positiva (PEEP) de no máximo 15cmH_2_0, níveis de pressão expiratória final suficientes para evitar o colabamento das vias aéreas e dos alvéolos e garantir uma troca gasosa adequada, posicionamento dos pacientes no leito de maneira a garantir uma ventilação adequada e não lesiva (como a posição prona nos casos de sindrome do desconforto respiratório agudo (SDRA) grave) e técnicas de suporte avançado (como a circulação extra-corpórea com remoção do gás carbônico (CO_2_) nos casos de SDRA refratária. Com o advento de ventiladores cada vez mais sofisticados e com possibilidades de ajuste fino de sensibilidade e de diversos mecanismos de disparo, de diferentes velocidades e aceleração de fluxo inspiratório, diversos mecanismos de término de tempo inspiratório e diversas opções de monitorização ocorre a possibilidade de ajuste de sincronia do paciente com o ventilador mecânico e a ventilação mecânica de acordo com a doença respiratória apresentada pelos pacientes, destacando-se o suporte ventilatório direcionado para as doenças restritivas diferentemente das obstrutivas. 

Assim, fez-se a necessidade de reunião de especialistas brasileiros envolvidos com suporte ventilatório: anestesia, terapia intensiva, pneumologia, fisioterapia, enfermagem, nutrição e fonoaudiologia para análise das evidências literárias disponíveis sobre o ventilação mecânica que, associado à experiência das diversas especialidades, pudessem orientar os profissionais das unidades de terapias intensivas brasileiras a como oferecer aos nossos pacientes com insuficiência respiratória um suporte respiratório seguro e eficaz, baseado nas melhores evidências disponíveis para evitarmos a possibilidade de lesão associada a ventilação mecânica. 

Assim, o objetivo do presente trabalho foi rever a literatura disponível sobre 29 subtemas relacionados ao suporte ventilatório do paciente em insuficiência respiratória e, após apresentação, discussão e aprovação em plenária composta pelos 58 especialistas, apresentá-las sob a forma de recomendações ou sugestões.

## Metodologia

A literatura disponível no MEDLINE (2003-2013) e na Cochrane Central Register of Controlled Trials (CENTRAL) foi revisada por especialistas com nível escolar superior (médicos intensivistas, pneumologistas e anestesiologistas, fisioterapeutas, enfermeiros, nutricionistas e fonoaudiólogos), dispostos em duplas para cada um dos 29 subtemas escolhidos sobre suporte ventilatório não invasivo e invasivo em pacientes com insuficiência respiratória.

Após a revisão dos artigos disponíveis na literatura cada dupla respondeu as perguntas formuladas pela comissão organizadora (constituída pelos Drs. Carmen Silvia Valente Barbas (Presidente do Comitê de Ventilação Mecânica da AMIB), Alexandre Marini Ísola (Coordenador Nacional do Curso de Ventilação Mecânica para Adultos em UTI da AMIB - VENUTI) e Augusto Manoel de Carvalho Farias, Coordenador da Comissão de Terapia Intensiva da SBPT), de acordo com os critérios previamente sugeridos por outros autores.^(^ 1 ^-^ 4 ^)^ Assim, usou-se o termo recomendação quando o nível de evidência era forte, com base em estudos randomizados com mais de cem pacientes, metaanálises e efeito tudo ou nada ou de segurança para os pacientes. O termo sugestão foi utilizado quando a evidência foi considerada fraca, ou seja, baseada em estudos observacionais, casos-controle, série de casos e ou experiência de especialistas para orientação do suporte ventilatório nacional de maneira eficaz e segura evitando-se os possíveis efeitos deletérios associados ao suporte ventilatório não adequado para nossos pacientes.

Os 58 especialistas participantes responderam as perguntas propostas durante 8 horas de reunião na sede da Associação de Medicina Intensiva Brasileira (AMIB) no dia 03 de agosto de 2013. Após a elaboração das respostas às perguntas formuladas baseadas nas evidências disponíveis na literatura e na experiência dos especialistas estas foram apresentadas em plenária para todos os 58 especialistas presentes no dia 04 de agosto de 2013 na Sede da Associação de medicina Intensiva Brasileira (AMIB), sendo discutidas, modificadas quando necessário, votadas e aprovadas de acordo com sugestões e observações dos especialistas presentes.

Todos os relatórios entregues pelas duplas foram reunidos pela Comissão Organizadora do projeto, que revisou, formatou e produziu o documento final, após revisão dos autores. A seguir, o documento foi encaminhado para impressão de manuais de beira de leito contendo as recomendações para distribuição as unidades de terapia intensiva de todo o Brasil e para publicação na Revista Brasileira de Terapia Intensiva (RBTI) e no Jornal Brasileiro de Pneumologia.

## Indicação de suporte ventilatório não invasivo e invasivo

**Comentário** - A VM substitui total ou parcialmente a ventilação espontânea e está indicada na insuficiência respiratória aguda (IRpA) ou crônica agudizada. A VM propicia melhora das trocas gasosas e diminuição do trabalho respiratório, podendo ser utilizada de forma não invasiva por meio de uma interface externa, geralmente uma máscara facial, e de forma invasiva por meio de um tubo endotraqueal ou cânula de traqueostomia. Ventilação não invasiva (VNI) utiliza uma pressão inspiratória para ventilar o paciente por meio de interface nasofacial (pressão inspiratória positiva (IPAP) e ou pressão de suporte (PSV)) e uma pressão positiva expiratória para manter as vias aéreas e os alvéolos abertos para melhorar a oxigenação, pressão expiratória positiva (EPAP) ou pressão expiratória final positiva (PEEP). No modo de pressão positiva contínua nas vias aéreas (CPAP), é administrada ao paciente, por interface nasofacial, somente uma pressão expiratória final contínua nas vias aéreas, e a ventilação do paciente é feita de forma totalmente espontânea.

Ventilação mecânica não invasiva com pressão positiva: quando começar

**Recomendação -** Em não havendo contraindicação (Quadro 1), os pacientes com incapacidade de manter ventilação espontânea (volume-minuto >4Lpm, PaCO_2_<50mmHg e pH>7,25) devem iniciar uso de VNI com dois níveis de pressão, com a pressão inspiratória suficiente para manter um processo de ventilação adequada, visando impedir a progressão para fadiga muscular e/ou parada respiratória.^(^ 5 ^)^

**Quadro 1:** Contraindicações a ventilação não invasiva DPOC - doença pulmonar obstrutiva crônica.

**Sugestão -** Pode-se usar VNI em pacientes com rebaixamento de nível de consciência devido à hipercapnia em doença pulmonar obstrutiva crônica (DPOC). A melhora da consciência deve ser evidente dentro de 1 a 2 horas após o início da VNI.^(^ 5 ^,^ 6 ^)^

**Recomendação - **Os pacientes que deterioram ou não melhoram devem ser imediatamente intubados pelo risco de perda de proteção da via aérea inferior e de parada respiratória.^(^ 5 ^)^

### Ventilação mecânica não invasiva com pressão positiva: quando descontinuar 

**Recomendação -** O uso de VNI deve ser monitorado por profissional da saúde à beira-leito, de 0,5 a 2 horas. Para ser considerado sucesso, devem ser observados: diminuição da frequência respiratória (f), aumento do volume corrente (VC), melhora do nível de consciência, diminuição ou cessação de uso de musculatura acessória, aumento da pressão parcial do oxigênio (PaO_2_) e/ou da saturação arterial periférica (SpO_2_), e diminuição da pressão parcial de gás carbônico (PaCO_2_) sem distensão abdominal significativa. Quando não há sucesso, realizar imediata intubação orotraqueal (IOT) e ventilação invasiva. Espera-se sucesso na população hipercápnica com o uso da VNI em 75% dos casos, e nos hipoxêmicos em cerca de 50%.^(^ 5 ^)^

### Ventilação mecânica não invasiva na exacerbação da asma

**Sugestão - **A VNI pode ser utilizada em conjunto com terapia medicamentosa para melhorar a obstrução ao fluxo aéreo e diminuir esforço respiratório em pacientes em crise asmática moderada e acentuada.^(^ 5 ^,^ 7 ^)^

Ventilação mecânica não invasiva na exacerbação aguda da doença pulmonar obstrutiva crônica

**Recomendação - **Deve-se usar VNI no tratamento da DPOC agudizada para diminuir a necessidade de intubação (risco relativo - RR de 0,41 [intervalo de confiança de 95% − IC95%: 0,33-0,53]), diminuição do tempo de internação no hospital e diminuição da mortalidade com um RR de 0,52 (IC95%: 0,35-0,76).^(^ 5 ^,^ 6 ^)^

Edema agudo de pulmão cardiogênico

**Recomendação - **Deve-se usar VNI (ventilação com pressão positiva bifásica (BIPAP) com EPAP 5 a 10 e pressão inspiratória positiva (IPAP) até 15cmH_2_O) e/ou CPAP de 5 a 10cmH_2_O nos pacientes com edema agudo de pulmão de origem cardiogênica visando diminuir a necessidade de intubação endotraqueal (RR de 0,53 [IC95%: 0,34-0,83]) e redução na mortalidade hospitalar (RR de 0,6 [IC95%: 0,45-0,84]).^(^ 5 ^,^ 8 ^,^ 9 ^)^

Ventilação mecânica não invasiva na síndrome de desconforto respiratório agudo 

**Sugestão -** Pode-se utilizar a VNI na síndrome de desconforto respiratório agudo (SDRA), especialmente nos casos de SDRA leve, com os cuidados de se observarem as metas de sucesso de 0,5 a 2 horas. No caso de não sucesso, evitar retardar a intubação.^(^ 5 ^,^ 10 ^)^

**Recomendação -** Na SDRA grave, evitar utilizar VNI, devido à alta taxa de falência respiratória e à necessidade de IOT, especialmente em pacientes com PaO_2_/FIO_2_ <140 e *Simplified Acute Physiology Score* (SAPS) II >35.^(^ 5 ^,^ 10 ^)^

Ventilação mecânica não invasiva na pneumonia adquirida na comunidade grave

**Sugestão - **Pode-se utilizar a VNI em pneumonia adquirida na comunidade (PAC) grave com insuficiência respiratória hipoxêmica, especialmente nos portadores concomitantes de DPOC com os cuidados de se observarem as metas de sucesso de 0,5 a 2 horas. No caso de não sucesso, evitar retardar a intubação.^(^ 5 ^,^ 11 ^)^

### Pós-extubação

**Recomendação -** A VNI deve ser utilizada visando encurtar a duração da ventilação invasiva (ação facilitadora da retirada da VNI), reduzir a mortalidade, diminuir as taxas de pneumonia associada à VM (PAV), gerando menos dias internação em unidade de terapia intensiva (UTI) e hospitalar na população de pacientes DPOC hipercápnicos.^(^ 5 ^,^ 12 ^,^ 13 ^)^

**Recomendação -** Usar VNI imediatamente após a extubação nos pacientes de risco (Quadro 2) para evitar IRpA e reintubação (ação profilática). ^(^ 5 ^,^ 12 ^-^ 15 ^)^

**Quadro 2:** Pacientes considerados em risco de falha de extubação que poderão se beneficiar do uso de ventilação não invasiva imediato após extubação (uso profilático)

**Recomendação -** Evitar o uso da VNI após novo quadro de insuficiência respiratória instalada (ação curativa).^(^ 5 ^,^ 12 ^-^ 16 ^)^

### Ventilação não invasiva em pós-operatório

**Recomendação - **A VNI para tratamento da IRpA no pós-operatório imediato de cirurgias abdominal e torácica eletivas deve ser utilizada associada à melhora da troca gasosa, à redução de atelectasias e à diminuição do trabalho respiratório, além de diminuição da necessidade de IOT e, possivelmente, da mortalidade. Deve ser utilizada com cautela, respeitando-se as limitações e contraindicações para sua utilização.^(^ 5 ^,^ 16 ^-^ 19 ^)^

**Sugestão -** Em cirurgias esofágicas, pode-se usar VNI para se evitar IRpA, mantendo-se pressões inspiratórias mais baixas (EPAP <8 e IPAP <15). A mesma sugestão vale para cirurgia torácica, cirurgia abdominal, cirurgia cardíaca, e cirurgia bariátrica.^(^ 5 ^,^ 17 ^-^ 19 ^)^

### Broncoscopia

**Sugestão - **A VNI pode ser utilizada durante e após a broncoscopia, visando diminuir o risco de complicações associadas ao procedimento em pacientes com hipoxemia grave refratária, insuficiência respiratória pós-operatória, ou DPOC grave.^(^ 5 ^)^ Cuidados especiais devem ser utilizados após procedimento de biópsia trasnbrônquica, mantendo-se as pressões de vias aéreas <20cmH_2_O e realizando radiografia de tórax, se descompensação clínica do paciente e após cerca de 6 horas do procedimento, para verificação de possível ocorrência de pneumotórax.

## Máscaras e ventiladores para aplicação de ventilação não invasiva

Ventiladores disponíveis no mercado brasileiro: características, vantagens e desvantagens

**Sugestão - **A VNI pode ser aplicada por meio de ventiladores portáteis, específicos para esse fim com compensação de vazamento, os quais devem ser acoplados a interfaces nasofaciais com circuito único e válvula exalatória localizada na própria máscara e, ventiladores invasivos microprocessados com programas específicos para esse fim, os quais devem ser acoplados a interfaces nasofaciais por meio de cotovelo e circuito duplo do próprio ventilador mecânico (Quadro 1 - material eletrônico suplementar ). O modo CPAP pode ser aplicado por meio de geradores de fluxo (CPAP de "parede") ^(20,21) ^(Quadro 3).

**Quadro 3:** Tipos de modos ventilatórios para suporte não invasivo CPAP - pressão positiva contínua nas vias aéreas; BIBAP - pressão positiva bifásica; EAP - edema agudo de pulmão; PO - pos-operatório; IPAP - pressão inspiratória positiva; EPAP - presssão expiratória positiva. *exceto se contraindicado.

### Reinalação de gás carbônico

**Sugestão -** Procurar evitar a reinalação de CO_2_ ou minimizá-la nos casos de uso dos ventiladores de circuito único. Os sistemas que apresentam orifícios de exalação na própria interface apresentam menor risco de reinalação quando comparados aos que têm esse orifício no circuito. Outros fatores que podem contribuir para a reinalação de CO_2_ são a utilização de PEEP baixa e o reduzido suporte pressórico, necessitando maior grau de atenção nessas situações.^(^ 22 ^)^

### Suplementação de oxigênio 

**Sugestão -** Em ventiladores com misturador de gás, o ajuste da suplementação de oxigênio (O_2_) é feita no próprio ventilador. Nos equipamentos de VNI portáteis, sem misturador de gás, suplementar O_2_ diretamente na máscara sempre depois da válvula de exalação, usando fonte de O_2_ externa. A FiO_2_ suplementada ao paciente depende do fluxo de O_2_, da posição da conexão da fonte de O_2 _no circuito, do grau de vazamento verificado no circuito ventilatório, do tipo de interface utilizada e dos níveis de IPAP e EPAP oferecidos. ^(^ 23 ^-^ 26 ^)^

### Monitorização na ventilação não invasiva

**Recomendação -** Monitorizar o volume corren te (VC), a frequência respiratória (f) e a saturação periférica de oxigênio (SpO_2_) durante uso da VNI. Quando disponível, realizar a monitorização gráfica. As assincronias, os escapes o auto-peep, os esforços ineficazes e o mecanismo de compensação do vazamento devem ser constantemente observados. ^(26,27)^

Indicação de uso das interfaces nas principais situações clínicas

**Recomendação** - Escolher uma interface adequada, a que melhor se adapte à face do paciente, visando à melhor eficiência clínica. 

**Recomendação - **Utilizar interfaces sem compressão nasal se o tempo estimado de VNI for >24 a 48 horas. 

**Recomendação** - Utilizar interface com válvula de PEEP se a opção for CPAP com gerador de fluxo. 

**Recomendação -** Na VNI com ventilador mecânico de UTI (microprocessado convencional) utilizar máscara conectada a circuito duplo. Na VNI com ventilador específico, utilizar máscara para circuito único^(20,23-25) ^(Quadro 4).

**Quadro 4:** Diferenças entre a ventilação não invasiva administrada pelos ventiladores portáteis específicos para ventilação não invasiva e dos ventiladores de unidade de terapia intensiva microprocessados com módulo de ventilação não invasiva UTI - unidade de terapia intensiva; PCV - ventilação controlada a pressão; VNI - ventilação não invasiva; O2 - oxigênio; PEEP - pressão expiratória final positiva.

### Adaptação e tolerância da interface 

Máscaras nasais

**Sugestão -** Podem ser usadas em IRpA leve, em pacientes com claustrofobia ou má adaptação à máscara facial. 

**Sugestão -** Podem-se combinar diferentes interfaces quando os pacientes necessitam de assistência ventilatória contínua para evitar os pontos isquêmicos devido à redução de fluxo sanguíneo secundária à pressão que a máscara exerce no rosto do paciente (Quadro 5).^(^ 25 ^)^

Máscaras oronasais (faciais)

**Recomendação** - Usar máscaras faciais na IRpA leve e moderada, visando à melhora rápida dos parâmetros fisiológicos (trocas gasosas e trabalho respiratório). Monitorizar tolerância e efeitos colaterais, como úlcera em pontos de apoio e distensão gástrica.

Máscara facial total e capacete

**Recomendação -** Utilizar nas situações de insuficiência respiratória hipoxêmica mais graves por permitir uma pressurização maior das vias aéreas. Por cobrir todo o rosto, distribui a pressão da máscara exercida na pele, evitando pontos de pressão em torno do nariz e reduzindo o risco de lesões cutâneas (Quadro 5).

**Quadro 5:** Vantagens e desvantagens dos diversos tipos de interfaces VNI - ventilação não invasiva; CO2 - dióxido de carbono; PEEP - pressão expiratória final positiva.

**Sugestão -** Pode ser utilizada a máscara do tipo capacete (Helmet), se disponível, nos casos de insuficiência respiratória menos grave. A mesma é hermeticamente fechada por uma almofada de ar ao redor do pescoço, inflada pelo próprio ventilador, tendo como pontos de contato o pescoço, os ombros e a região axilar. Porém, o fato de apresentar um grande espaço morto gera uma limitação para sua aplicação em pacientes com distúrbio de ventilação, devendo ser corrigida com aplicação de maiores níveis de suporte pressórico. O ruído interno também é um incômodo a ser considerado. Essa interface pode gerar também assincronia de disparo determinado pelo atraso na liberação do fluxo inspiratório, com consequente elevação do trabalho respiratório^(28-30) ^(Quadro 5).

## Intubação e traqueostomia

### Técnicas para intubação eletiva, semieletiva e na emergência

**Recomendação -** Usar laringoscopia direta com visualização da laringe como o mais rápido e confiável método de inserção do tubo orotraqueal, seja em casos eletivos e na emergência. Após três tentativas de intubação sem sucesso, por médico experiente, considera-se via aérea difícil (VAD), devendo seguir as normatizações estabelecidas para esse fim.^(^ 31 ^,^ 32 ^)^

Intubação eletiva 

**Sugestão -** A intubação traqueal eletiva é caracterizada quando não há sinais iminentes de falência nos mecanismos de proteção das vias aéreas, de ventilação e/ou oxigenação, podendo ser escolhido o melhor método para intubação traqueal de acordo com cada paciente. Usar a laringoscopia direta com IOT como método de escolha.^(^ 31 ^,^ 32 ^)^

**Sugestão -** Preparar o paciente adequadamente para intubação traqueal, por meio de pré-oxigenação, monitorização e posicionamento adequado durante o procedimento, visando a uma laringoscopia ótima.^(^ 32 ^,^ 33 ^)^

**Sugestão -** Utilizar preferencialmente o laringoscópio de lâmina curva de número apropriado. O laringoscópio de lâmina reta pode ser usado para exposição da laringe em intubações difíceis.^(^ 31 ^,^ 32 ^,^ 34 ^)^

Intubação de emergência

**Sugestão -** Utilizar a técnica de intubação com sequência rápida para reduzir o risco de aspiração de conteúdo gástrico. Nessa técnica, posicionar o tubo orotraqueal no menor tempo possível, após a perda da consciência.^(^ 32 ^,^ 35 ^,^ 36 ^)^

**Sugestão -** Utilizar agentes hipnóticos (propofol, etomidato, cetamina ou tiopental), opioides (fentanil, alfentanil ou remifentanil) e bloqueadores neuromusculares (rocurônio ou succinilcolina). A manobra de Sellick (compressão da cricoide) pode ser utilizada, durante o procedimento para minimizar risco de aspiração de conteúdo gástrico.^(^ 32 ^,^ 35 ^-^ 37 ^)^

### Técnicas e indicação de traqueostomia: vantagens e desvantagens

Tempo de realização de traqueostomia: recomendações baseadas na causa da insuficiência respiratória

Trauma raquimedular

**Sugestão -** Realizar traqueostomia precoce (até 7 dias) nesse grupo. O trauma cervical alto - C5 ou acima - é um fator preditor independente de necessidade VM prolongada. Os pacientes com lesões abaixo desse nível devem ser avaliados individualmente.^(^ 32 ^,^ 38 ^)^

Trauma cranioencefálico

**Sugestão - **Realizar traqueostomia precoce (até 7 dias) nos pacientes mais graves (escala de Glasgow <8), que geralmente necessitam de suporte ventilatório prolongado. Há evidência conflitante na redução da taxa de pneumonia associada à VM e não há evidência que a traqueostomia precoce reduza a mortalidade, a lesão das vias aéreas e nem a internação hospitalar.^(^ 32 ^,^ 38 ^,^ 39 ^)^

Pacientes com trauma que não incluem o sistema nervoso central

**Sugestão -** Indicar a traqueostomia precoce quando houver estimativa de suporte ventilatório prolongado.^(^ 32 ^,^ 38 ^-^ 40 ^)^

Pacientes internados por causas clínicas na unidade de terapia intensiva

**Recomendação -** Aguardar 14 dias para realização da traqueostomia, uma vez que o procedimento precoce não reduz a mortalidade em 30 dias, tampouco o tempo de UTI e a necessidade de sedação.^(^ 32 ^,^ 41 ^-^ 44 ^)^

### Técnicas de traqueostomia

**Recomendação** - Realizar traqueostomia percutânea ou convencional de acordo com os recursos disponíveis e experiência da equipe. A traqueostomia percutânea pode ser realizada à beira do leito pela equipe da terapia intensiva, mas apresenta custo maior e necessita de broncoscopia, visando aumentar a segurança do procedimento, apresentando menor taxa de infecção na ferida cirúrgica. A traqueostomia convencional deve ser realizada em centro cirúrgico por equipe especializada, salvo unidades de terapia intensiva que possuam sala para procedimentos cirúrgicos internos. Em relação às complicações maiores, como taxa de sangramento, enfisema subcutâneo, pneumotórax e mortalidade, ambas as técnicas apresentam resultados semelhantes.^(^ 32 ^,^ 45 ^-^ 47 ^)^

## Regulagem inicial do ventilador invasivo e modos ventilatórios convencionais

### Regulagem do ventilador

**Recomendação -** Utilizar a FIO_2_ necessária para manter a saturação periférica de oxigênio (SpO_2_) entre 93 a 97%.^(^ 48 ^,^ 49 ^)^

**Recomendação -** Usar VC 6mL/kg/peso predito inicialmente. Reavaliar de acordo com evolução do quadro clínico do paciente.^(^ 48 ^-^ 52 ^)^

**Recomendação - **Usar modo assistido-controlado, podendo ser ciclado a volume (VCV) ou ciclado a tempo e limitado a pressão (PCV), reavaliando nas primeiras horas de acordo com o quadro clínico.^(^ 48 ^-^ 51 ^)^

**Recomendação -** Regular frequência respiratória (f) inicial controlada entre 12 e 16rpm, com fluxo inspiratório ou tempo inspiratório visando manter inicialmente relação I:E em 1:2 a 1:3. Em caso de doença obstrutiva, pode-se começar usando f mais baixa (<12rpm) e, em caso de doenças restritivas, pode-se utilizar f mais elevada (>20rpm, por exemplo, se o quadro clínico assim exigir). Reavaliar assim que disponível a primeira gasometria.^(^ 48 ^,^ 51 ^-^ 54 ^)^

**Recomendação -** Definir o tipo de disparo do ventilador. Os disparos mais comuns no mercado são os disparos a tempo (modo controlado pelo ventilador) e pelo paciente (disparos a pressão e a fluxo, chamados de modos de disparo pneumáticos). A sensibilidade do ventilador deve ser ajustada para o valor mais sensível para evitar autodisparo. O ventilador ainda pode ser disparado pelo estímulo neural (modo de ventilação assistida ajustada neuralmente - NAVA).^(^ 48 ^,^ 51 ^-^ 54 ^)^

**Recomendação -** Usar PEEP de 3 a 5cmH_2_O inicialmente, salvo em situações de doenças como SDRA, nas quais o valor da PEEP deve ser ajustado de acordo com as orientações abordadas no tema específico nestas recomendações.^(^ 48 ^,^ 49 ^,^ 55 ^-^ 57 ^)^

**Recomendação -** Utilizar aquecedores e umidificadores passivos em pacientes sob VM. Nos pacientes portadores de secreção espessa, devem-se utilizar umidificação e aquecimento ativos, se disponível com umidificação ótima, para evitar oclusão do tubo orotraqueal.^(^ 58 ^)^

**Recomendação -** Regular os alarmes de forma individualizada, usando critérios de especificidade e sensibilidade adequados para o quadro clínico do paciente. Devem-se regular o *back-up* de apneia e os parâmetros específicos de apneia, se disponíveis no equipamento. 

**Recomendação -** Uma vez estabelecidos os parâmetros iniciais, observar as curvas de VC, pressão e fluxo, a fim de constatar se os valores obtidos estão dentro do previsto e se não há necessidade de reajuste imediato. Verificar a oximetria de pulso que deve ser contínua. Inicialmente, usar o alarme de pressão máxima nas vias aéreas em 40cmH_2_O, visando evitar barotrauma, ajustando-se conforme quadro clínico, assim que possível.^(^ 48 ^,^ 51 ^-^ 54 ^)^

**Recomendação - **Após 30 minutos de ventilação estável, deve-se colher uma gasometria arterial, para observar se as metas de ventilação e troca foram atingidas. Do contrário, realizar os reajustes necessários nos parâmetros de modo e ciclagem.^(^ 48 ^-^ 51 ^)^

**Recomendação -** Avaliar as possíveis repercussões hemodinâmicas da VM. Avaliar presença de hipovolemia/ocorrência de auto-PEEP e/ou pneumotórax em casos de hipotensão associada ao uso da ventilação com pressão positiva. 

**Recomendação -** Manter o nível de trabalho muscular o mais apropriado. Nos casos de demanda de fluxo inspiratório alta, utilizar opioides para diminuição do *drive *ventilatório e adequado conforto do paciente. Proporcionar o repouso muscular por 24 a 48 horas nos casos de fadiga muscular respiratória e de instabilidade hemodinâmica.

**Recomendação -** Nos casos em que o repouso muscular não se faz necessário, iniciar o mais rápido possível um modo assistido de ventilação com adequado ajuste da sensibilidade do ventilador. Evitar a disfunção diafragmática induzida pelo ventilador, que geralmente ocorre após 18 horas de ventilação controlada.

**Sugestão -** Em pacientes com idade avançada, com uso prolongado de modos controlados, pacientes desnutridos, pacientes sob uso de corticoides, bloqueadores neuromusculares e hipotireoidismo dar especial atenção à avaliação da função da musculatura respiratória. 

### Modos ventilatórios convencionais(59)

**Sugestão -** Utilizar modos assisto-controlados a volume (VCV) quando se almejar manter volume-minuto (VC x f) mais estável. Esse modo pode ser disparado a tempo (controlado), pressão e fluxo (assistido) e é ciclado ao se atingir o VC inspirado predeterminado. A pressão nas vias aéreas é variável e consequente à mecânica ventilatória do paciente (especial atenção à monitorização à pressão de pico e de platô nesse modo, com adequada regulagem de alarme de pressão máxima em vias aéreas). Esse modo é utilizado para medida da pressão de pico e pressão de platô visando calcular complacência e resistência do sistema respiratório sob fluxo inspiratório constante e quadrado (vide tema específico destas recomendações).

**Sugestão -** Utilizar modos assisto-controlados limitados a pressão e ciclados a tempo (PCV) em situação de comprometimento da mecânica do sistema respiratório (complacência baixa e ou resistência alta), pois permitem o controle mais adequado das pressões em vias aéreas e alveolares. Esse modo se caracteriza por manter a pressão limitada durante toda a fase inspiratória, sendo ciclado a tempo. O tempo inspiratório é fixo em segundos pelo cuidador. O fluxo é livre e desacelerado. Nesse modo o VC é variável e consequente do delta de pressão administrado e da mecânica ventilatória do paciente (deve-se oferecer especial atenção à monitorização do VC expirado e regulagem de alarme de volume-minuto máximo e mínimo). Pode-se, ainda, acelerar ou desacelerar a velocidade do fluxo inspiratório (rampa, *rise time* ou *slope*). O *rise time* pode ser mais acelerado em pacientes obstrutivos, visando ajustar um melhor VC. Cuidado especial com a ocorrência de pico de fluxo excessivo (*overshoot*). Nos pacientes restritivos, usar *rise time* menos acelerado.

**Sugestão -** A pressão de suporte (PSV) é considerada o modo preferencial durante a ventilação assistida ou espontânea. Deve ser iniciado seu uso o mais precocemente possível, conforme o quadro clínico. É modo disparado exclusivamente pelo paciente, a pressão ou a fluxo. Caracteriza-se por pressão limitada durante toda fase inspiratória, sendo ciclado quando o fluxo inspiratório cai, geralmente, a 25% do pico de fluxo inspiratório. Esse critério de ciclagem (% de critério de ciclagem) em alguns ventiladores mais modernos pode ser regulado de 5 a 80%, permitindo redução do tempo inspiratório em pacientes obstrutivos (% de critério de ciclagem >25%) e aumento do tempo inspiratório em pacientes restritivos (% de critério de ciclagem <25%). O *rise time* pode ser mais acelerado em pacientes obstrutivos, permitindo-se diminuir o tempo inspiratório e ajustar um melhor VC. Cuidado especial com a ocorrência de pico de fluxo excessivo (*overshoot*). Nos pacientes restritivos, usar *rise time* menos acelerado, com possível ganho de VC.

**Sugestão -** Usar ventiladores ciclados a pressão quando somente houver esse recurso no local. Pode ser disparado a tempo e a pressão. Caracteriza-se por oferecer fluxo fixo até que a pressão das vias áreas atinja um valor predeterminado regulado pelo cuidador (ciclagem). Assim, o VC não é conhecido, recomendando-se utilização de um ventilômetro externo (ventilômetro de Wright) ou coletar uma gasometria arterial após 20 minutos de ventilação estável, a fim de checar se o valor de PaCO_2_ está compatível com o quadro clínico (entre 35 a 45mmHg na maioria dos casos). Esse equipamento geralmente não dispõe de *blender* (misturador) interno de O_2_, nem de alarmes. Deve haver cuidadosa atenção e vigilância da ventilação e oxigenação por parte da equipe multiprofissional. 

**Recomendação - **Evitar o uso do modo *Synchronized Intermitent Mandatory Ventilation* (SIMV), pois se mostrou associado a aumento do tempo de retirada da VM. Atualmente, o uso dessa modalidade se restringe a pacientes que necessitem garantir volume-minuto mínimo no início da ventilação com pressão de suporte (PSV) (por exemplo: neuropatas ou pacientes no despertar inicial de anestesia geral). Assim que o controle (*drive*) ventilatório se mostrar estável, deve-se modificar para modo PSV. Descrição do modo: os ciclos controlados podem ser ciclados a volume (SIMV-V) ou limitados a pressão (SIMV-P). Os ciclos espontâneos devem ser associados a PSV. Caracteriza-se por permitir dentro da mesma janela de tempo (JT), que é determinada pela frequência respiratória do modo controlado, ciclos controlados, assistidos e espontâneos. Ciclo controlado somente ocorre se não tiver havido disparo assistido na JT imediatamente anterior. Do contrário, o *software* do ventilador aguarda o próximo disparo do paciente em ciclo assistido. No restante da JT, podem ocorrer ciclos espontâneos, apoiados pela PSV.

## Assincronia e novos modos em ventilação mecânica

### Assincronia paciente-ventilador

**Comentário -** Assincronia paciente-ventilador é a incoordenação entre os esforços e as necessidades ventilatórias do paciente em relação ao que é ofertado pelo ventilador.^(^ 60 ^)^ São eventos frequentes, presentes em 10 a 80% de todos os ciclos e que se associam a prolongamento da VM e da internação na UTI.^(^ 61 ^)^

**Recomendação - **A presença de assincronias e suas correções devem ser buscadas ativamente durante a avaliação do paciente em VM.

### Assincronias de disparo

Disparo ineficaz

**Comentário -** O esforço inspiratório do paciente não é suficiente para disparar o ventilador.^(^ 62 ^)^ Pode ocorrer por ajuste inadequado da sensibilidade ou por fatores do paciente, como fraqueza da musculatura respiratória, depressão do comando neural, presença de hiperinsuflação dinâmica (auto-PEEP) ou tempo inspiratório mecânico prolongado maior que o tempo neural do paciente. ^(62,63)^

**Identificação** - Clinicamente, percebe-se o esforço inspiratório do paciente tocando seu tórax ou abdome, observando que o mesmo não se acompanha de um ciclo fornecido pelo ventilador. ^(64,65) ^A figura 1A mostra como identificar essa assincronia com as curvas do ventilador.^(^ 64 ^,^ 65 ^)^

**Figura 1:** Assincronias de disparo identificadas nas curvas de volume, fluxo e pressão versus tempo e assinaladas com setas. As deflexões negativas nas curvas pressão versus tempo representam os esforços do paciente (pressão muscular), visíveis somente se monitorizada a pressão esofágica. Painel A) Esforços perdidos. Na primeira seta, observar um estímulo débil, incapaz de disparar o ventilador, resultando em uma pequena onda de fluxo positiva e em mínimo volume corrente. Na segunda seta, observar o esforço que ocorre durante a exalação, sem disparar o ventilador, apenas fazendo com que o fluxo volte à linha de base e até se tornar levemente positivo. Painel B) Duplo disparo. Exemplo em volume controlado. Os esforços do paciente permanecem no momento da ciclagem e, assim, disparam novo ciclo. Os volumes se somam (empilhamento), e a pressão nas vias aéreas se eleva, disparando muitas vezes o alarme de alta pressão. Painel C) Autodisparo: na modalidade pressão de suporte, alguns ciclos são disparados sem esforços do paciente, tendo sido favorecidos pela presença de vazamentos, visíveis na curva volume versus tempo, a qual não retorna à linha de base (medida do volume inspirado maior do que a do expirado). Figuras obtidas pelo Xlung.net, simulador virtual de ventilação mecânica. Disponível em: http//:www.xlung.net.

**Recomendação -** Para resolução da assincronia de disparo, a sensibilidade deve ser ajustada para o valor mais sensível possível, evitando-se, porém o autodisparo, ou ainda modificar o tipo de disparo de pressão para fluxo (geralmente mais sensível). 

**Sugestão -** Na vigência de auto-PEEP, uma PEEP extrínseca pode ser titulada de 70 a 85% da auto-PEEP, verificando-se os efeitos sobre a assincronia.^(^ 62 ^)^ Durante a PSV, pode-se tentar a redução dos níveis de pressão administrados ou o aumento da porcentagem do critério de ciclagem.^(^ 63 ^)^ Na modalidade pressão-controlada (PCV) pode-se tentar reduzir o tempo inspiratório e na volume-controlada (VCV), aumentar o fluxo inspiratório ou diminuir a pausa.^(^ 62 ^,^ 63 ^)^

Duplo disparo

**Comentário - **Ocorrem dois ciclos consecutivos disparados pelo mesmo esforço do paciente. O tempo inspiratório mecânico do ventilador é menor que o tempo inspiratório neural do paciente.^(3)^

**Identificação** - Clinicamente percebem-se dois ciclos consecutivos sem intervalo entre eles, em um padrão que pode se repetir com frequência. A figura 1B mostra como identificar essa assincronia com as curvas do ventilador.^(^ 64 ^-^ 66 ^)^

**Sugestão -** Em VCV, deve-se aumentar o fluxo inspiratório e/ou o volume corrente, respeitando-se os limites de segurança. Outra opção é a mudança para a modalidade PCV ou PSV, nas quais o fluxo inspiratório ofertado varia conforme os esforços do paciente. Caso o duplo disparo ocorra na PCV, pode-se aumentar o tempo inspiratório e/ou o valor da pressão controlada (PC). Na PSV, pode-se tentar aumentar o nível de pressão ou reduzir a porcentagem do critério de ciclagem.^(62,63)^

Autodisparo

**Comentário -** O ventilador é disparado sem que haja esforço do paciente. Pode ocorrer por ajuste excessivamente sensível do ventilador, por vazamento no sistema, presença de condensado no circuito gerando alterações no fluxo, detecção dos batimentos cardíacos e de grandes variações da pressão torácica pela ejeção do volume sistólico (Figura 1C).^(60,62)^

**Identificação** - Observação de frequência respiratória maior que a ajustada e sem que os ciclos sejam precedidos de indicadores de esforço do paciente.^(^ 64 ^-^ 67 ^)^

**Recomendação -** Descartadas ou corrigidas as presenças de vazamentos ou condensado no circuito, deve-se reduzir progressivamente a sensibilidade o suficiente para que autodisparos desapareçam.^(^ 62 ^,^ 64 ^-^ 66 ^)^

Assincronias de fluxo

Fluxo inspiratório insuficiente

**Comentário -** O fluxo recebido pelo paciente é inferior à sua demanda ventilatória, ocorrendo tipicamente quando o fluxo é ajustado pelo operador e não pode ser aumentado pelos esforços do paciente como na modalidade VCV. Entretanto, pode ocorrer também nas modalidades PCV e PSV, se os ajustes de pressão forem insuficientes em relação ao equilíbrio entre demanda e capacidade ventilatória do paciente.^(67,68)^

**Identificação** - Clinicamente, o paciente encontra-se desconfortável, com utilização de musculatura acessória. A figura 2 mostra como identificar essa assincronia com as curvas do ventilador.^(^ 67 ^,^ 68 ^)^

**Figura 2:** Assincronia de fluxo. Na modalidade volumecontrolada, o fluxo foi ajustado aquém da demanda do paciente, que mantém esforço muscular durante toda a inspiração, a qual passa a apresentar uma concavidade voltada para cima. Essa assincronia está representada com intensidade progressiva do primeiro para o terceiro ciclo na figura. As deflexões negativas nas curvas pressão versus tempo representam os esforços do paciente (pressão muscular), sendo visualizadas somente quando se monitoriza a pressão esofágica. Figuras obtidas pelo Xlung.net, simulador virtual de ventilação mecânica. Disponi´vel em: http//:www. xlung.net.

**Recomendação -** Corrigir as causas de aumento da demanda ventilatória, tais como febre, dor, ansiedade e acidose. Aumentar o fluxo inspiratório na VCV, observando o conforto e a conformação da curva pressão *versus* tempo; mudança do modo para PCV ou PSV, que têm fluxo livre;^(^ 68 ^)^ ajuste da velocidade com que a pressão limite é alcançada nas vias aéreas (*rise time* - tempo de subida ou ascensão ou aumentando o valor de pressão controlada).^(^ 69 ^)^

Fluxo inspiratório excessivo

**Comentário -** Pode ocorrer em VCV, quando o fluxo é ajustado acima do desejado pelo paciente, ou em PCV ou PSV, pelo ajuste de pressões elevadas ou de um *rise time* mais rápido. 

**Identificação** - Na VCV, a curva pressão *versus* tempo mostra o pico de pressão sendo alcançado precocemente.^(68,69) ^Na PCV ou PSV, a pressão nas vias aéreas ultrapassa o nível ajustado, fenômeno denominado *overshoot* .^(^ 69 ^)^

**Recomendação -** Na VCV, o fluxo deve ser reduzido; na PCV e na PSV, o *rise time* deve ser diminuído até que desapareça o *overshoot*.^(^ 68 ^)^

Assincronias de ciclagem

Ciclagem prematura 

**Comentário -** O ventilador interrompe o fluxo inspiratório antes do desejado pelo paciente, ou seja, o tempo inspiratório mecânico do ventilador é menor que o tempo neural do paciente.^(^ 70 ^)^ Nas modalidades VCV e PCV, o tempo inspiratório é ajustado pelo operador. Na PSV, ela ocorre por ajuste de baixo nível de pressão e/ou alta porcentagem de critério de ciclagem.^(^ 70 ^)^ A figura 3 mostra como identificar essa assincronia com as curvas do ventilador. Em alguns casos, o esforço pode ser suficiente para deflagrar um novo ciclo (dupla-ciclagem).^(64,66,70)^

**Figura 3:** Assincronias de ciclagem durante a pressão de suporte. No primeiro ciclo, o ponto de corte de 25% do pico fluxo foi atingido rapidamente (porcentagem de critério de ciclagem), fazendo com que o tempo inspiratório do ventilador tenha sido menor que o desejado pelo paciente. Isso pode ser observado pela porção expiratória da curva de fluxo, que tende a voltar para a linha de base em função do esforço ainda presente do paciente. O último ciclo representa o contrário, ou seja, ciclagem tardia. A redução do fluxo se faz de forma muito lenta, fenômeno típico de obstrução ao fluxo aéreo, fazendo com que o limiar de ciclagem demore a ser atingido. Algumas vezes o ciclo é interrompido pela contração da musculatura expiratória, que gera uma elevação acima da pressão de suporte ajustada ao final da inspiração (não representada nesta figura). Figuras obtidas pelo Xlung.net, simulador virtual de ventilação mecânica. Disponi´vel em: http//:www.xlung.net.

**Recomendação -** Em VCV, deve-se diminuir o fluxo inspiratório e/ou aumentar o volume corrente, respeitando-se os limites de segurança. Outra opção é a mudança para a modalidade PCV ou PSV, nas quais o fluxo inspiratório ofertado varia conforme os esforços do paciente. Caso a ciclagem prematura ocorra na PCV, pode-se aumentar o tempo inspiratório e/ou o valor da PC. Na PSV, pode-se tentar aumentar o nível de pressão ou reduzir a % do critério de ciclagem. ^(^ 62 ^,^ 63 ^,^ 70 ^)^

Ciclagem tardia

**Comentário -** O tempo inspiratório mecânico do ventilador ultrapassa o desejado pelo paciente, ou seja, é maior que o tempo neural do paciente. Em VCV, ocorre quando se prolonga o tempo inspiratório pelo ajuste de volume corrente alto, fluxo inspiratório baixo, e/ou uso de pausa inspiratória de forma inadequada. Na PCV, ocorre se o tempo inspiratório for ajustado além do desejado pelo paciente. Em PSV, particularmente nas doenças obstrutivas, como a DPOC a alta resistência e a complacência do sistema respiratório levam à desaceleração do fluxo inspiratório de forma lenta, prolongando o tempo inspiratório.^(70) ^A figura 3 mostra como identificar essa assincronia com as curvas do ventilador.^(^ 64 ^,^ 66 ^)^

**Recomendação -** Nas modalidades em que o operador ajusta o tempo inspiratório, este deve ser reduzido. Em PSV, pode-se elevar a porcentagem de critério de ciclagem (por exemplo, de 25% para 40% ou até mais).^(70)^

**Sugestão **- A assincronia paciente-ventilador deve ser tratada com ajuste dos parâmetros ventilatórios ou utilização de outros modos ventilatórios (opinião de especialistas).

### Modos avançados de ventilação mecânica

**Comentário -** A escolha do modo ventilatório deve ser baseada em função da gravidade do paciente.^(71) ^Para pacientes com insuficiência respiratória com assincronia, uma mudança de modo ventilatório pode ser uma alternativa. Nos últimos anos, houve um aumento significativo do número e da complexidade dos modos ventilatórios. Apesar de sua crescente disponibilidade, o impacto clínico da utilização desses novos modos ainda é pouco estudado.^(^ 71 ^)^

**Sugestão** - Usar os modos avançados em situações clínicas específicas, desde que o usuário esteja familiarizado com seus ajustes e que o quadro clínico venha a se beneficiar dos recursos específicos de cada modo.

Volume controlado com pressão regulada (pressure-regulated volume-control)

**Comentário -** É um modo ventilatório ciclado a tempo e limitado à pressão. A cada ciclo, o ventilador reajusta o limite de pressão, baseado no volume corrente obtido no ciclo prévio, até alcançar o volume corrente alvo ajustado pelo operador.^(72)^

**Sugestão -** Indicar quando se almeja controle do volume corrente com pressão limitada, visando a ajustes automáticos da pressão inspiratória se a mecânica do sistema respiratório se modificar.

**Recomendação - **Deve-se ter cuidado ao ajustar o volume corrente, pois esse ajuste pode levar a aumentos indesejados da pressão inspiratória.

Ventilação com liberação de pressão nas vias aéreas e ventilação com pressão positiva bifásica

**Comentário -** O modo ventilação com liberação de pressão nas vias aéreas (APRV, do inglês *airway pressure-release ventilation*) é limitado a pressão e ciclado a tempo, sendo considerado um modo espontâneo. O operador ajusta a pressão superior (PEEPhigh) e a inferior (PEEPlow) e a relação PEEPhigh: PEEPlow, bem como a frequência de alternância entre os dois níveis de PEEP, sendo obrigatoriamente o tempo em PEEPhigh superior a tempo de PEEPlow. O modo BIPAP também usa dois níveis de PEEP, porém com tempo de PEEPlow mais longo que o PEEPhigh. O paciente consegue respirar espontaneamente em qualquer dos níveis de pressão.^(73,74) ^Pode-se adicionar pressão de suporte, cujo valor será somado ao valor de PEEPlow, sendo a pressão final nas vias aéreas (Paw) a soma de PSV + PEEPlow. Se o valor de PEEPhigh for inferior ao valor de PSV+PEEPlow, durante a PEEPhigh o ventilador apenas complementa o valor de PSV para atingir o mesmo valor de Paw medido durante PEEPlow com PSV.

**Sugestão -** Utilizar o APRV quando houver necessidade de manutenção da ventilação espontânea, do recrutamento alveolar com potencial melhora das trocas gasosas e de redução do espaço morto e da assincronia. Pode ser usado em pacientes com SDRA como estratégia protetora, desde que gere baixos volumes correntes.

**Recomendação - **Cuidado com a regulagem da alternância entre os níveis de pressão, pois o volume-minuto nesse modo é a soma dos VC obtidos, quando alternam-se as pressões mais o VC do paciente (com ou sem pressão de suporte).

### Ventilação assistida proporcional 

**Comentário -** O modo ventilação assistida proporcional (PAV, sigla do inglês *proportional assist ventilation)* é um modo espontâneo que utiliza a equação do movimento para oferecer pressão inspiratória (Pvent) proporcional ao esforço do paciente (Pmus). Caso a Pmus se reduza, a Pvent também se reduz e vice-versa.^(71,75-79) ^Alguns estudos mostraram melhor sincronia paciente-ventilador com o PAV e sua versão mais recente, PAV *plus*, em comparação com a PSV. O PAV *plus* estima o trabalho ventilatório (WOB) do paciente e do ventilador mecânico, usando a equação do movimento, e calcula a complacência e a resistência por meio da aplicação de micropausas inspiratórias de 300ms a cada quatro a dez ciclos ventilatórios. 

**Indicações** - Para pacientes com *drive* respiratório, apresentando assincronia significativa em modo espontâneo, em especial PSV. Quando se almeja conhecer o WOB do paciente e as medidas de mecânica durante ventilação assistida, como, por exemplo, estimativa de PEEP intrínseca em tempo real.^(^ 75 ^-^ 79 ^)^

**Recomendação - **O cuidador deve ajustar no ventilador antes do início do modo PAV *plus* o tipo de prótese traqueal, o diâmetro da mesma, o tipo de umidificador, o VC máximo e a Pmáxima em vias aéreas permitida (limites).

**Recomendação -** Usar valor de porcentagem de apoio inicial de 50%, visando manter o WOB do paciente entre 0,3 e 0,7 J/L com adequado VC e f. A Pvent é maior quanto maior for a Pmus do paciente. Evitar ultrapassar 90% de porcentagem de apoio. Nessa necessidade, melhor optar por modos de ventilação convencionais assisto-controlados. Diminuir porcentagem de apoio progressivamente em função da melhora clínica do paciente até 30%. Se mantidos os parâmetros adequados (acima), extubar paciente. 

**Sugestão -** O modo PAV é uma alternativa ao PSV para pacientes com assincronia significativa, com potencial de melhorar a interação paciente-ventilador.

**Recomendação -** Deve-se evitar em pacientes sem *drive* respiratório, bem como VM com vazamentos que prejudiquem as medidas de resistência e complacência.

### Compensação automática do tubo

**Comentário -** A compensação automática do tubo (ATC, sigla do inglês *automatic tube compensation*) é um modo espontâneo que tem como objetivo diminuir o trabalho resistivo imposto ao paciente pela presença da via aérea artificial - tubo orotraqueal ou tubo de traqueostomia. Alguns estudos mostraram menor trabalho respiratório e maior conforto com o ATC quando comparado com o modo PSV.^(80-82)^

**Sugestão -** Utilizar, associado ou não à PSV, visando à compensação do aumento do trabalho resistivo associado à presença da prótese traqueal de forma automática (em PSV, essa compensação deve ser calculada pelo cuidador em virtude do diâmetro da prótese, oferecendo-se valores maiores de PSV para tubos com diâmetros menores. Por exemplo: PSV=5cmH_2_O para tubo de diâmetro 9 e PSV=9cmH_2_O para tubos de diâmetro 6).

**Recomendação -** Contraindicar para pacientes sem *drive* respiratório e cuidado com excesso de secreções que interfiram no fluxo inspiratório; é importante garantir que alarmes de pressão de vias aéreas estejam bem ajustados.

### Ventilação assistida ajustada neuralmente

**Comentário -** A NAVA (sigla do inglês *neurally adjusted ventilatory assist*) é um modo ventilatório que captura a atividade elétrica do diafragma e a utiliza como critério para disparar e ciclar o ventilador, oferecendo suporte inspiratório proporcional à atividade elétrica do diafragma. Para funcionar, o modo NAVA precisa que seja locado um cateter esofagogástrico com sensores posicionados no um terço distal do esôfago, capazes de captar a atividade elétrica do diafragma. ^(5,6) ^Em estudos clínicos, o NAVA associou-se à melhora da sincronia com o ventilador em comparação com PSV. 

**Indicações** - Para pacientes com *drive* respiratório, apresentando assincronia significativa em modo espontâneo, em especial esforços perdidos em PSV, como nos pacientes com auto-PEEP (PEEP intrínseca).^(^ 77 ^-^ 79 ^,^ 83 ^)^

**Recomendação -** Maior cuidado em pacientes com doenças oronasais ou esofágicas que possam impedir a passagem ou o posicionamento adequado do cateter de NAVA; Deve-se posicionar e fixar bem o cateter de NAVA, com sua posição sendo verificada periodicamente. Após a fixação da sonda, iniciar a medida de atividade elétrica do diafragma (Edi) e ajustar o ganho de NAVA (NAVA *gain*) de acordo com o VC, a frequência respiratória e a pressão nas vias aéreas (Edi *versus* NAVA *gain*). O disparo do ventilador ocorre por variação de 0,5µV da Edi. A partir daí, o ventilador envia fluxo livre em função da leitura da Edi. A pressão máxima alcançada nas vias aéreas é o resultado da soma da multiplicação do Edi máximo - Edi mínimo pelo NAVA *gain* ao valor da PEEP extrínseca. A ciclagem ocorre com queda da Edi para 70% do pico máximo de Edi detectado.^(^ 77 ^-^ 79 ^,^ 83 ^)^

**Recomendação - **O NAVA *gain* é ajustado de acordo com o quadro clínico apresentado, avaliando-se caso a caso.

**Sugestão -** O NAVA é uma alternativa ao PSV para pacientes com assincronia significativa, com potencial de melhorar a interação paciente-ventilador, em especial para pacientes com esforços perdidos.

### Ventilação de suporte adaptativa 

**Comentário -** A ventilação de suporte adaptativa (ASV, sigla do inglês *adaptive-support ventilation*) utiliza um algoritmo para escolher a combinação entre volume corrente e frequência respiratória, visando atingir ao volume-minuto regulado pelo cuidador, por meio de ciclos espontâneos e controlados, com a mínima pressão de vias aéreas possível. A versão denominada Intellivent-ASV, usa um sensor de CO_2_ no final de expiração (ETCO_2_) e um sensor de SpO_2_ para ajustar automaticamente PEEP e FIO_2_, utilizando uma tabela.^(^ 83 ^)^

**Indicações** - Pacientes com insuficiência respiratória grave, para os quais buscam-se redução do trabalho respiratório e estímulo para respirações espontâneas. 

**Sugestão -** Usar para garantir um volume-minuto com adequada proteção pulmonar em pacientes com controle ventilatório (*drive*) instável, com assincronia ou desconforto. Monitorizar ocorrência de vazamentos ou secreção excessiva que podem comprometer o seu funcionamento adequado.

## Ventiladores para aplicação de ventilação invasiva

### Escolha do ventilador mecânico

As seguintes perguntas devem ser respondidas na escolha dos ventiladores mecânicos: em que população de pacientes será utilizado o ventilador (população adulta, pediátrica, neonatal), com que frequência se internam pacientes com elevada dificuldade ventilatória (como pacientes com SDRA, doenças obstrutivas graves, fístulas pulmonares etc.), quais informações o ventilador pode fornecer que auxiliam nas decisões do suporte ventilatório naquela UTI, como se vai realizar a retirada da VM dos pacientes, qual modo ventilatório, quais medidas clínicas e de mecânica irão auxiliar na decisão e com que frequência e em que situações se vai utilizar a VNI. 

**Sugestão -** Avaliar características particulares dos diferentes ventiladores, de acordo com os recursos e as necessidades de sua unidade:

**Ventiladores com recursos básicos**. Apresentam um ou mais modos básicos sem curvas. Em sua maioria são ventiladores utilizados para transporte de pacientes em VM. 

**Ventiladores com recursos básicos com curvas**. Apresentam os modos básicos de ventilação (VCV, PCV, SIMV e PSV) e curvas básicas de ventilação (volume, fluxo e pressão).

**Ventiladores com curvas e recursos avançados de ventilação**. Apresentam além dos modos básicos e das curvas básicas, modos avançados como modos com duplo controle (PRVC, por exemplo), modos diferençados para ventilação espontânea (como PAV-plus, NAVA) e formas avançadas de monitorização (como medida de trabalho, P 0,1, PImax, capnometria volumétrica e calorimetria indireta).

**Recomendação -** No ambiente hospitalar, todos os ventiladores utilizados devem ter ao menos: (1) o controle de volume corrente expirado (VCe), (2) monitorização básica (no mínimo de pressão inspiratória), e (3) e, acoplado ao ventilador, um misturador de gases (*blender*), evitando necessidade de O_2_ suplementar na via artificial.

**Recomendação -** Ventiladores usados especificamente em UTI devem ter como pré-requisitos mínimos, além dos anteriores: (1) monitorização de curvas (pelo menos pressão-tempo) e (2) alarmes (pelo menos de pressão de vias aéreas máxima e mínima, para detecção de apneia e desconexão).

**Comentário -** No material eletrônico suplementar, apresenta-se a lista de ventiladores mecânicos pulmonares para adultos em VM invasiva, disponíveis e comercializados no Brasil (em agosto/2013) acompanhados de algumas de suas características (Quadros 2, 3, 4 e 5 do material suplementar). Não foram incluídos nessa lista ventiladores de uso exclusivo: (1) para VNI; (2) neonatal e pediátrico; (3) domiciliar/apneia do sono; e (4) para anestesia.

## Monitorização do paciente com suporte ventilatório

### Monitorização das trocas gasosas

Como fazer a monitorização da mecânica ventilatória à beira do leito

**Recomendação -** Deve-se fazer a monitorização da mecânica ventilatória de rotina em todo paciente submetido a suporte ventilatório mecânico invasivo, sendo compreendidos os seguintes parâmetros: VCe (volume corrente expirado), pressão de pico (pressão inspiratória máxima), pressão de platô ou de pausa inspiratória (em ventilação controlada), PEEP extrínseca, auto-PEEP ou PEEP intrínseca. ^(^ 84 ^-^ 88 ^)^

**Sugestão -** Fazer os cálculos de resistência de vias aéreas (Rva), complacência estática (Cst), e monitorar as curvas de fluxo, pressão e volume *versus* tempo em casos selecionados.^(^ 84 ^-^ 88 ^)^

**Comentário -** A mensuração da pressão alveolar na prática clínica pode ser obtida por meio de uma pausa inspiratória de pelo menos 2 segundos de duração. A pressão ao final da pausa é denominada de pressão de platô ou pressão de pausa. O fluxo inspiratório para a medição deve ser do tipo "quadrado" para cálculo da Rva, e deve ser convertido para litros/segundo.

**Recomendação -** São requisitos para mensuração acurada da pressão de pausa: ausência de esforço muscular respiratório, tempo de pausa de 2 a 3 segundos e ausência de vazamentos. ^(^ 84 ^-^ 87 ^)^

**Recomendação -** Evitar valores >28 a 30cmH_2_O da pressão alveolar, que indicam baixa complacência estática do sistema respiratório. Deve-se verificar a possível causa (alteração do parênquima pulmonar e/ou da caixa torácica). No primeiro caso, deve-se diminuir VC e/ou pressão de distensão. No segundo caso, podem existir outras causas a serem analisadas: diminuição da complacência da parede torácica e/ou hipertensão intra-abdominal. Nesse último caso, deve-se monitorar a pressão intra-abdominal e indicar conduta de descompressão, quando necessário. ^(84-88) ^A figura 4 ilustra a forma de cálculo da Rva e da Cst.

**Figura 4:** Manobra de pausa inspiratória e obtenção da resistência das vias aéreas e pressão de pausa (ou platô). VCV - volume controlado. Pva - pressão nas vias aéreas; PEEP - pressão expiratória final positiva; VC - volume corrente; Pel - pressão elástica; Palv - pressão alveolar.

**Comentário -** A auto-PEEP ou PEEPi ocorre quando a pressão alveolar ao final da fase expiratória é superior à pressão das vias aéreas devido a um esvaziamento incompleto do sistema respiratório. 

**Recomendação - **Identificar a auto-PEEP pela inspeção da curva de fluxo *versus* tempo, na qual o fluxo expiratório não volta a zero ao final da expiração.^(^ 84 ^-^ 87 ^)^

**Recomendação -** A medida da auto-PEEP ou PEEP intrínseca deve ser feita durante a ventilação controlada, realizando-se uma pausa ao final da expiração (chamada pausa expiratória), respeitados os mesmos cuidados da medida da pressão de pausa inspiratória.^(^ 84 ^-^ 87 ^)^

**Recomendação -** nos casos de SDRA, deve-se monitorizar a pressão de distensão ou também conhecida como *driving pressure*, obtida pela subtração Pplatô - PEEP. A pressão de distensão deve ficar obrigatoriamente ≤15cmH_2_O nos casos de SDRA moderada e grave, quando se necessitar de PEEP mais elevada, que acabe por elevar a Pplatô entre 30 e 40cmH_2_O (vide tema de VM na SDRA, nestas recomendações).^(^ 89 ^-^ 91 ^)^

### Monitorização da troca gasosa na ventilação mecânica

Gasometria arterial

**Recomendação - **Coletar gasometria arterial preferencialmente na artéria radial ou femoral em todos os casos de IRpA o mais rápido possível para estabelecimento do raciocínio clínico e da conduta terapêutica. Esse exame permite a avaliação diagnóstica do estado metabólico acidobásico e da troca gasosa pulmonar com medidas diretas do pH, PaCO_2_, PaO_2_ e cálculo da SaO_2_, HCO_3-_ e excesso de bases (EB). Se suspeita de intoxicação causando metemoglobinemia e carboxihemoglobinemia, determinar a SaO_2_ diretamente por co-oximetria.^(^ 92 ^,^ 93 ^)^

**Recomendação -** Realizar a coleta de gasometria em todos os pacientes sob suporte ventilatório cerca de 20 minutos após o ajuste inicial dos parâmetros do ventilador e diariamente, enquanto durar a fase aguda do quadro. Deve-se coletar nova amostra em caso de mudança no quadro clínico do paciente.^(^ 92 ^,^ 93 ^)^

**Recomendação -** Evitar coleta de gasometria em situação de risco de isquemia tissular no território irrigado pela artéria a ser puncionada e se houver infecção no sítio de punção. Nas coagulopatias e plaquetopenias, deve ser coletada se absolutamente necessária.^(^ 92 ^,^ 93 ^)^

Cuidados na realização da gasometria

**Sugestão -** Utilizar *kits* padronizados ou seringas de 5mL com heparina lítica ou sódica em quantidades mínimas e agulha fina (23 a 25G) de preferência com mecanismo de proteção. ^(^ 92 ^,^ 93 ^)^

**Recomendação -** O procedimento deve ser feito com técnica asséptica, por ser invasivo. Sempre que possível, o procedimento deve ser explicado ao paciente e realizado somente com seu consentimento.^(^ 92 ^,^ 93 ^)^

**Recomendação -** Deve-se comprimir o local da punção por 5 minutos, e por mais tempo em caso de coagulopatias ou uso de anticoagulantes. ^(^ 92 ^,^ 93 ^)^

**Recomendação -** A amostra deve ser levada e analisada o mais rapidamente possível. Em caso de necessidade de transporte para análise fora da unidade, deve-se levá-la em recipiente refrigerado.^(^ 92 ^,^ 93 ^)^

Cuidados na interpretação da gasometria

**Recomendação -** Registrar os seguintes parâmetros no momento da coleta: FIO_2_, volume corrente, frequência respiratória, PEEP, SpO_2_ e o PETCO_2_, se em uso de capnógrafo. 

**Recomendação -** Realizar para todos os pacientes o cálculo da relação PaO_2_/FIO_2_ para avaliação da eficiência da oxigenação e análise da evolução clínica do paciente.^(^ 92 ^,^ 93 ^)^

**Sugestão - **Registrar se há o uso de posição prona, qual o modo ventilatório em uso durante a coleta, se ocorreram manobras de recrutamento alveolar ou titulação da PEEP pré-coleta.

**Comentário -** A gasometria arterial retrata apenas um determinado momento do paciente. Para monitorização contínua a oximetria de pulso e a capnografia são os melhores métodos.

Oximetria de pulso

**Recomendação -** Realizar a monitorização contínua por oximetria de pulso em todo o paciente sob suplementação de O_2_, VNI ou suporte ventilatório invasivo e nos casos de IRpA

Capnografia

**Recomendação -** Usar em pacientes sob suporte ventilatório com doenças neurológicas, para confirmação de adequado posicionamento da prótese ventilatória, e em todas as situações de retenção de CO_2_ acima de 50mmHg.

**Sugestão -** Pode-se usar como monitorização em quadros de distúrbios de ventilação/perfusão para detecção de alterações agudas e monitorização de terapias específicas (terapia trombolítica no tromboembolismo pulmonar - TEP, por exemplo).

### Monitorização regional

Monitorização por tomografia de impedância elétrica

**Comentário -** Tomografia de impedância elétrica (TIE) é uma técnica não invasiva que se utiliza da mensuração da passagem da corrente elétrica entre eletrodos colocados ao redor do tórax, permitindo identificar os territórios mais ou menos resistentes à passagem da corrente. É utilizada para monitorização da ventilação e, mais recentemente, da perfusão pulmonar à beira do leito de forma contínua.^(^ 94 ^-^ 98 ^)^

**Sugestão -** Usar a TIE para detecção de alterações da ventilação pulmonar como pneumotórax, posição de tubo endotraqueal, alterações da ventilação conforme o decúbito, avaliação de recrutamento e colapso pulmonares, e distribuição regional da ventilação. Futuramente esse método pode vir a ser utilizado para monitorização da perfusão pulmonar.^(^ 94 ^-^ 98 ^)^

Tomografia computadorizada 

**Recomendação -** Usar como método diagnóstico nos casos de insuficiência respiratória de origem não clara; usar a angiotomografia na suspeita de embolia pulmonar.

**Sugestão -** Nos centros disponíveis, pode ser utilizada para monitorização do recrutamento alveolar e titulação decremental da PEEP nos casos de SDRA moderada e grave, com os cuidados pertinentes no transporte e considerando-se a taxa de radiação total ao paciente.^(^ 99 ^,^ 100 ^)^

Ecografia torácica (ultrassom de tórax)

**Recomendação -** Nos centros com a tecnologia disponível, deve-se treinar a equipe para usar o ultrassom do tórax na identificação precoce de pneumotórax, derrame pleural e como auxiliar no procedimento terapêutico. 

**Sugestão -** Pode ser utilizada para estimar reaeração alveolar em pacientes tratados para pneumonia associada a VM, avaliação de edema pulmonar, para detectar atelectasia pós-extubação e para estimar o recrutamento pulmonar induzido por PEEP.^(^ 101 ^-^ 103 ^)^

## Sedação e analgesia durante ventilação mecânica

### Quando indicar e como administrar sedativos e analgésicos?

**Sugestão -** Realizar a utilização de sedação e analgesia durante VM para auxiliar no controle da ansiedade, agitação e dor. A sedação adequada auxilia a promoção da tolerância do paciente ao ventilador, aos procedimentos terapêuticos e diagnósticos.^(^ 104 ^,^ 105 ^)^

**Recomendação -** O nível de sedação deve ser leve a moderado, para permitir mobilização precoce.^(^ 106 ^)^

**Recomendação -** Titular propofol e midazolam para níveis de sedação leve, moderada e profunda. A dexmedetomidina não deve ser utilizada para sedação profunda. Os opioides recomendados são fentanil, morfina e remifentanil.^(^ 107 ^)^

**Sugestão -** Evitar que cetamina seja utilizada como agente sedativo principal em pacientes em VM. A cetamina pode ser útil em situações em que há necessidade de seu efeito poupador de opioides.^(^ 107 ^,^ 108 ^)^

**Sugestão - **Dominar o conhecimento das principais drogas utilizadas para analgesia e sedação no paciente sob suporte ventilatório:

**Propofol -** Ação principal por agonista ácido gama-aminobutírico (GABA). Possui efeito sedativo, hipnótico, ansiolítico, anticonvulsivante e promove amnésia. Não possui efeito analgésico. Apresenta depressão respiratória dose-dependente e hipotensão secundária à vasodilatação sistêmica - principalmente após bólus. Infusão prolongada pode tornar o despertar imprevisível e ocasionar a síndrome de infusão do propofol - incidência ≤1%. A síndrome possui alta mortalidade e é caracterizada por piora da acidose metabólica, hipertrigliceridemia, hipotensão com necessidade maior de vasopressor e arritmias. A dose inicial recomendada é de 5mcg/kg/minuto, por 5 minutos, seguida da infusão contínua de 5 a 50mcg/kg/minutos.^(^ 107 ^)^

**Midazolam -** É um agonista GABA. Promove ansiólise, amnésia e hipnose. Possui efeitos anticonvulsivantes. Não possui efeito analgésico. A utilização de benzodiazepínico para hipnose parece estar relacionada a maior incidência de *delirium*. Em relação ao propofol, o midazolam pode ocasionar tempo de VM prolongada. Síndrome de abstinência pode ocorrer após infusão prolongada, isto é, de duração maior que 7 dias. A dose inicial recomendada é de 0,01 a 0,05mg/kg e a dose de manutenção, em infusão contínua, é de 0,02 a 0,1mg/kg/h.^(^ 109 ^)^

**Dexmedetomidina -** É um agonista alfa-adrenérgico do tipo 2 de ação central. Possui efeito sedativo e auxilia na redução da necessidade de analgésicos/opioides. Não possui efeitos anticonvulsivantes. Não apresenta depressão respiratória significativa. Não é um fármaco apropriado para sedação profunda. Em relação à prevalência de *delirium*, há uma redução com o uso de dexmedetomidina quando comparada ao benzodiazepínico. No paciente de terapia intensiva, opta-se por não utilizar a dose de ataque preconizada e iniciar com o fármaco em infusão contínua. Após início da infusão, o início de ação é em 15 minutos e o efeito máximo em 1 hora. A dose preconizada é de até 1,4mcg/kg/hora.^(^ 107 ^)^

**Fentanil - **Apresenta rápido início de ação e alta potência. Não libera histamina. Tende ao acúmulo com a duração da infusão contínua e na presença de disfunção hepática e, em alguns pacientes, pode ocorrer rigidez torácica. A dose inicial recomendada é de 50 a 100mcg. Em infusão contínua, a taxa de infusão recomendada é de 0,7 a 10mcg/kg/h.^(^ 108 ^,^ 110 ^)^

**Morfina -** Opioide mais utilizado para períodos de exacerbação da dor. Para titulação a beira leito, utilizam-se de 1 a 2mg a cada 10 minutos até analgesia adequada ou aparecimento de efeitos colaterais. Em infusão contínua, a dose recomendada é de 2 a 30mg/h. Tende ao acúmulo na disfunção hepática e renal. Libera histamina.^(^ 110 ^)^

**Remifentanil -** Opioide com potência analgésica semelhante ao fentanil. Metabolizado por estearases plasmáticas e com perfil farmacológico que não favorece o acúmulo, mesmo após infusão prolongada. Não apresenta efeito analgésico residual. Dose de ataque recomendada: 1,5mcg/kg em aproximadamente 3 a 5 minutos. Dose de manutenção recomendada: 0,5 a 015mcg/kg/hora.^(^ 110 ^)^

### Quando utilizar bloqueio neuromuscular?

**Recomendação **- Utilizar cisatracúrio nas primeiras 48 horas nos quadros de SDRA com relação PaO_2_/FiO_2 _< 120 para manter VM controlada. A utilização de bloqueio neuromuscular está condicionada a uma sedação profunda e à monitorização adequada do nível de consciência. A dose sugerida é de 37,5mg/hora.^(^ 111 ^)^

### Como monitorar o paciente sedado em ventilação mecânica?

**Recomendação -** Monitorar o nível de sedação por meio das escalas Sedation and Agitation Scale (SAS) ou *Richmond Agitation and Sedation Scale* (RASS) - ferramentas para uso clínico validadas para a UTI e que devem ser aplicadas de maneira sistemática e por equipe treinada.^(^ 112 ^,^ 113 ^)^

**Sugestão -** Usar monitores da atividade cerebral como eletroencefalografia contínua (EEC) contínuo ou Biespectral index system (BIS) quando houver bloqueio neuromuscular ou quando a utilização de escalas não for possível, a fim de avaliar o grau de sedação.

### Como proceder a retirada da sedação

**Recomendação -** Realizar a interrupção diária da sedação em pacientes em VM, quando a gravidade do quadro clínico permitir. O paciente que já se encontra acordado ou facilmente desperto e cooperativo com a estratégia de sedação atual não tem necessidade de ter sua sedação suspensa ou interrompida.^(^ 110 ^,^ 114 ^)^

**Recomendação -** Avaliação frequente e rotineira de dor e *delirium* pois são etiologias comuns de despertar agitado, com tratamento específico. A manutenção do quadro pode contribuir para aumento do tempo de VM e dificuldade da retirada da VM.^(^ 110 ^)^

## Ventilação mecânica na asma

**Comentário - **A crise asmática grave põe em risco a vida do paciente. Na última década, houve redução da morbimortalidade relacionadas a essa condição em consequência da utilização de estratégias ventilatórias que visam à redução da hiperinsuflação alveolar.^(^ 115 ^)^

### Indicações de ventilação mecânica 

**Recomendação -** Indicações recomendadas de VM invasiva na asma: parada cardíaca; parada respiratória; rebaixamento de consciência, escala de coma de Glasgow <12; hipoxemia (PaO_2_<60mmHg; SpO_2_<90%) não corrigida com máscara (FiO_2 _40-50%); arritmia grave; fadiga progressiva (hipercapnia progressiva).

**Sugestão -** Indicações sugeridas de VM invasiva na asma: isquemia miocárdica; acidose lática após tratamento com broncodilatadores.^(116,117)^

### Intubação dos pacientes com crise de asma

### **Recomendação - **Aplicar sequência rápida de intubação. 

**Sugestão -** Posicionar o paciente com elevação da cabeceira de 20 a 30**°** (diminui risco de regurgitação passiva e aspiração).

**Recomendação -** Realizar pré-oxigenação com máscara O_2_ ou BIPAP; ambu de forma gentil (oito ciclos respiratórios).

**Sugestão -** Realizar premedicação com lidocaína endovenosa (EV) 1,5mg/kg; 3 minutos pré-intubação (diminui reflexo simpático, náusea e vômito) e fentanil 3mcg/kg (diminui reflexo simpático; pode causar depressão respiratória).

### **Sugestão - **Não usar manobra de Sellick.

**Recomendação -** Na indução para intubação optar por: cetamina 1 a 2mg/kg EV; propofol 2 a 2,5mg/kg EV ou etomidato 0,2 a 0,3mg/kg EV.

**Recomendação -** Para realizar o relaxamento muscular usar: rocurônio 0,9mg/kg, ou succinilcolina 1 a 1,5mg/kg EV (fasciculação pode aumentar risco de regurgitação e aspiração). ^(^ 118 ^,^ 119 ^)^

**Sugestão -** Opcionalmente para o relaxamento muscular usar vecurônio 0,3mg/kg (desvantagem: início de efeito 60-90 segundos).

**Sugestão -** Usar tubo de maior calibre possível. Se viável, >8mm de diâmetro interno.

### Programação do ventilador 

**Sugestão - **Modalidade: PCV ou VCV; volume corrente: 6mL/kg peso predito (inicialmente); pressão inspiratória máxima: <50cmH_2_O; pressão de platô: <35cmH_2_O; auto-PEEP: <15cmH_2_O; frequência respiratória: 8 a 12/minuto; fluxo: necessário para manter tempo expiratório suficiente para terminar expiração; 60 a 100L/minuto (VCV); livre (PCV); FiO_2_: necessário para manter SpO_2_ >92%; PaO_2_>60mmHg;PEEP: baixa (3 a 5cmH_2_O); em casos selecionados e com monitorização adequada a PEEP pode ser usada em valores superiores pelo efeito mecânico em abrir as pequenas vias aéreas.^(116,117)^

### Monitorizção do paciente e redução de hiperinsuflação 

**Recomendação - **Os pacientes asmáticos em VM devem ser monitorizados periodicamente com o objetivo de identificar hiperinsuflação alveolar (pressão de platô e a PEEP intrínseca) e cálculo da resistência de vias aéreas. A pressão de pico não é uma medida representativa de hiperinsuflação alveolar.^(^ 120 ^,^ 121 ^)^

**Recomendação - **Utilizar VC de 5 a 6mL/kg − peso predito. Em casos de hiperinsuflação refratárias às medidas convencionais, considerar volumes inferiores a 5mL/kg e f mais baixas (10 a 12rpm) visando evitar hiperinsuflação alveolar. Essa estratégia pode levar à hipercapnia, que deve ser monitorizada para se manter PaCO_2_ <80mmHg e pH>7,20 (hipercapnia permissiva).

**Sugestão -** Utilizar PEEP, como estratégia de redução da hiperinsuflação alveolar. Nesse caso, ventilar o paciente em PCV com pressão de distensão ≤15cmH_2_O. Ao se aumentar a PEEP, se ocorrer aumento do volume expiratório, isto sugere redução da hiperinsuflação alveolar ou desinsuflação. 

**Recomendação -** Monitorizar a mecânica ventilatória em caso de instabilidade hemodinâmica, visando identificar se há auto-PEEP, a fim de reajustar parâmetros para melhora da hemodinâmica.^(^ 122 ^)^

**Recomendação -** Solicitar radiografia de tórax em caso de instabilidade hemodinâmica, pelo risco de pneumotórax.^(120,121)^

**Recomendação -** A retirada da ventilação deve ser iniciada tão logo haja controle do broncoespasmo e da hiperinsuflação alveolar.^(^ 1 ^,^ 8 ^)^

**Sugestão -** O paciente asmático pode ser extubado sob sedação leve. 

**Sugestão -** Em casos de dificuldade de progredir o desmame ventilatório, avaliar possibilidade de fraqueza da musculatura ventilatória por polineuropatia associada ao uso de corticoide e curare.

## Analgesia e sedação(123-126)

**Sugestão -** Sugere-se não usar morfina devido à possibilidade de aumentar a liberação de histamina. Não se deve usar meperidina devido à possibilidade de aumentar a liberação de histamina. Pode-se usar Fentanil 1 a 3mcg/kg/hora; Alfentanil 0,5 a 1mcg/kg/minuto; Sufentanil 0,5mcg/kg/hora; Ketamina 0,25 a 0,5mg/kg/hora (broncodilatador); Propofol 0,3 a 4mg/kg/hora (broncodilatador); Midazolam 0,04 a 0,06mg/kg/hora (3 a 5mg/hora). 

## Relaxamento muscular(126-128)

**Recomendação - **O relaxamento muscular pode ser utilizado para intubação, na fase inicial da VM, se necessário. Seu uso prolongado deve ser evitado por causa de miopatia e neuropatia (risco aumentado por uso concomitante de corticoides). 

**Recomendação -** Sobre o uso de relaxantes musculares durante a VM: recomenda-se o uso de Rocurônio: 1mg/kg - início 45 segundos - duração 45 minutos. Como antídoto, pode-se usar o Sugammadex(r) se necessário.

**Sugestão - **Em relação aos relaxantes musculares durante a VM são opções sugeridas: Vecurônio: 0,15mg/kg - início 75-90 segundos - duração 30 minutos; Succinilcolina: 1 a 1,2mg/kg (até 1,5), para intubação na indução. Essa última é contraindicada se história de hipertermia maligna, doença neuromuscular, distrofia muscular, hiperpotassemia, rabdomiólise, queimaduras até 72 horas, acidente vascular cerebral (AVC) até 72 horas; 

**Sugestão - **Não usar pancurônio** - **embora apresente risco baixo de liberação de histamina, esse risco é superior ao do vecurônio e rocurônio.

**Recomendação - **Não usar atracúrio e cisatracúrio, por risco alto de liberação de histamina.

Tratamento adicional - uso de anestésicos, heliox e oxigenação extracorpórea por membrana

**Sugestão -** Utilizar anestésicos halogenados (por exemplo: isoflurano) administrados pelo ventilador de anestesia para possível controle do broncoespasmo refratário às medidas terapêuticas habituais, evitando-se essa terapia por mais de 12 horas. Cuidado especial com a monitorização de lesão hepática durante o uso.^(^ 122 ^)^

**Sugestão - **Heliox pode diminuir a resistência das vias aéreas e melhorar a chegada do broncodilatador aos pulmões, podendo ser tentado em casos refratários e nos serviços que disponham do equipamento apropriado para uso desse gás. ^(^ 129 ^)^

**Sugestão -** Oxigenação extracorpórea por membrana (ECMO). Considerar em casos graves aqueles que não respondem os tratamentos anteriores.^(^ 130 ^)^

## Ventilação mecânica doença pulmonar obstrutiva crônica

### Indicações de ventilação mecânica invasiva

**Recomendação -** Considerar VM invasiva quando houver contraindicação para VNI ou quando houver falha no uso da VNI (25% dos casos). Otimizar o tratamento medicamentoso.

**Sugestão -** Na IOT, utilizar cânulas com maior diâmetro possível, idealmente >8mm, para reduzir a resistência das vias aéreas e facilitar a remoção das secreções.^(^ 131 ^-^ 133 ^)^

### Objetivos da ventilação mecânica

**Recomendação -** Promover repouso da musculatura respiratória, promover a melhora dos distúrbios agudos da troca gasosa, redução da hiperinsuflação pulmonar e otimização da sincronia paciente-ventilador.^(^ 131 ^,^ 134 ^,^ 135 ^)^

### Modo ventilatório inicial

**Sugestão -** Utilizar qualquer um dos modos ventilatórios (volume controlado ou pressão controlada) na abordagem inicial da exacerbação da DPOC, desde que haja monitorização adequada e familiaridade da equipe com o mesmo.^(^ 131 ^-^ 136 ^)^

### Fração inspirada de oxigênio 

**Sugestão -** Ajustar a FiO_2_ com base na gasometria arterial e na oximetria de pulso de modo a utilizar-se a menor FIO_2_ que mantenha a SaO_2_ entre 92 e 95% e PaO_2_ entre 65 e 80mmHg. ^(^ 131 ^)^

### Volume corrente

**Recomendação -** Utilizar volumes correntes baixos, de 6mL/kg do peso predito.^(131,132-136) ^Nos modos PCV e PSV, monitorizar excessos de volume corrente que podem ocorrer com valores baixos de pressão.

### Frequência respiratória e volume-minuto

**Recomendação - **Programar a frequência respiratória inicial entre 8 e 12 por minuto. O volume-minuto deve ser ajustado para normalizar o pH arterial, e não a PaCO_2_.^(^ 131 ^,^ 132 ^-^ 136 ^)^

### Fluxo inspiratório e relação inspiração:expiração 

**Recomendação -** No modo volume controlado, utilizar fluxos inspiratórios desacelerados de 40 a 60L/min, com ajuste da relação inspiração:expiração (I:E) em valores <1:3, permitindo um tempo expiratório prolongado, suficiente para promover a desinsuflação pulmonar e melhorar o aprisionamento aéreo. No modo pressão controlado, ajustar o menor valor de pressão de distensão, visando atingir um tempo inspiratório suficiente para ocorrer a zeragem do fluxo inspiratório pelo ventilador (tempo de enchimento pulmonar). Deve-se manter uma relação I:E em valores inferiores a 1:3 visando ao tempo expiratório suficiente, com o mínimo de auto-PEEP.^(^ 131 ^,^ 132 ^-^ 136 ^)^

### Aplicação de PEEP na ventilação controlada

**Sugestão -** Aplicar PEEP externa para contrabalançar a auto-PEEP secundária à limitação ao fluxo aéreo expiratório, como tentativa de desinsuflação pulmonar, desde que associados a uma monitorização adequada da mecânica respiratória. Com esse intuito, deve ser utilizada a medida da pressão de platô no modo VCV e no PCV.^(131,137,138) ^No modo VCV, a desinsuflação induzida pela PEEP externa pode ser detectada pela manutenção ou pela queda da pressão de platô. Se, por outro lado, a pressão de platô aumentar, a PEEP externa pode ocasionar hiperinsuflação pulmonar adicional e ser reduzida ou retirada. No modo PCV, à medida que se aumenta o valor da PEEP externa, monitora-se o volume corrente exalado. Se este for reduzido, há piora da hiperinsuflação e a PEEP externa deve ser reduzida ou retirada. Se, por outro lado, o volume corrente exalado aumentar, a PEEP externa ocasiona desinsuflação pulmonar e pode ser mantida.^(^ 131 ^,^ 137 ^,^ 138 ^)^

### Aplicação de PEEP na ventilação assistida/espontânea

**Sugestão -** No caso dos ventiladores com disparo a pressão, os pacientes com auto-PEEP podem ter dificuldade em iniciar o ciclo assistido, apresentando assincronia. Nesses casos, usar disparo a fluxo e/ou aplicar PEEP externa no valor de aproximadamente 85% da auto-PEEP, para facilitar ao paciente atingir o limiar de disparo do ventilador.^(^ 134 ^,^ 139 ^,^ 140 ^)^

### Monitorização da ventilação mecânica

**Recomendação -** Realizar a monitorização da mecânica respiratória e da hiperinsuflação pulmonar na exacerbação da DPOC. Os principais parâmetros a serem monitorizados são: pressão de platô, pressão de pico, auto-PEEP, resistência das vias aéreas e as curvas: fluxo *versus* tempo, volume *versus* tempo e pressão *versus* tempo. Em crises de broncoespasmo graves, pressão de pico de até 45cmH_2_O pode ser tolerada, desde que acompanhada de pressão de platô ≤30cmH_2_O. ^(^ 131 ^,^ 132 ^)^

### Retirada da ventilação mecânica

**Sugestão -** Pacientes com DPOC geralmente apresentam maior dificuldade para adequada interação paciente-ventilador. Desse modo, sugerem-se utilizar modos que promovam um melhor conforto do paciente e sua monitorização. PSV é um modo ventilatório útil, quando bem ajustado. Cuidado especial deve se ter com valores elevados de pressão de suporte, que podem dificultar ciclagem e piorar a interação paciente-ventilador, aumentando a auto-PEEP. Ventilação proporcional assistida plus (PAV+) e ventilação assistida com ajuste neural (NAVA) são modos promissores para melhora da interação paciente-ventilador, mas são necessárias maiores evidências para uso rotineiro.^(^ 141 ^-^ 143 ^)^

**Sugestão -** Pacientes com DPOC apresentam menor desaceleração do fluxo inspiratório, podendo ter aumento do tempo inspiratório em PSV com a sensibilidade expiratória habitual (25%). Nos ventiladores que permitem o ajuste da ciclagem da PSV (porcentagem de critério de ciclagem, sensibilidade expiratória ou *cycling-off criteria*), ajustar a sensibilidade expiratória de ciclagem para níveis mais elevados (40 a 60%), visando diminuir o tempo inspiratório, o VC e a chance de assincronia paciente.^(^ 144 ^,^ 145 ^)^

**Sugestão -** Para redução do tempo inspiratório e consequente prolongamento do tempo expiratório, ajustar a aceleração do fluxo inspiratório (*rise time*) em valores mais elevados, tendo cuidado para evitar a subida excessiva do fluxo inspiratório (*overshoot*) e a monitorização do conforto do paciente.^(^ 144 ^,^ 145 ^)^

**Recomendação -** Utilizar do recurso da VNI para retirada precoce da ventilação mecânica invasiva em pacientes com DPOC exacerbado após períodos de 24 a 48 horas de repouso muscular, com equipes treinadas e de acordo com os critérios tratados no tema específico nestas recomendações.^(^ 146 ^)^

### Administração de broncodilatadores inalatórios

**Sugestão -** Administrar as medicações broncodilatadoras por via inalatória por meio de nebulizador ou *spray* dosimetrado acoplado a espaçador. O spray dosimetrado apresenta as vantagens de maior facilidade de manipulação, reprodutibilidade da dose e menor risco de contaminação.^(147) ^Quando agonistas beta-2adrenérgicos são administrados por meio de *spray *dosimetrado, sugere-se a dose de quatro jatos (inicialmente, com intervalo de 20 minutos até três vezes e, no tratamento de manutenção, a cada 2 a 4 horas).^(^ 148 ^)^

## Ventilação mecânica na pneumonia adquirida na comunidade

**Comentário - **Estas recomendações aplicam-se as pacientes com pneumonia comunitária e pneumonia associada aos cuidados da saúde e referem-se à ventilação mecânica invasiva e VNI. Quando a pneumonia associar-se a SDRA, ver tema específico nestas recomendações.

### Ventilação mecânica não invasiva 

**Sugestão -** Utilizar VNI de forma cuidadosa em pacientes com pneumonia grave. O uso de VNI deve ser monitorado por profissional da saúde à beira do leito de 0,5 a 2 horas. Para ser considerado sucesso, devem ser observados diminuição da f, aumento do VC, melhora do nível de consciência, diminuição ou cessação de uso de musculatura acessória, aumento da PaO_2_ e/ou da SpO_2_ e diminuição da PaCO_2_ sem distensão abdominal significativa. Quando não há sucesso, realizar imediata IOT e ventilação invasiva, já que a intubação tardia diminui a sobrevida. Em três situações a VNI pode usada com melhor resposta: pacientes com insuficiência cardíaca esquerda sistólica ou diastólica; doença pulmonar obstrutiva crônica com retenção de CO_2_ e acidose e imunossuprimidos com pneumonia bilateral. Espera-se sucesso na população hipercápnica com o uso da VNI em 75% dos casos, e em 50% dos hipoxêmicos.^(^ 149 ^,^ 150 ^)^

### Modo ventilatório

**Sugestão -** A escolha do modo ventilatório^(151-154) ^deve se basear em três critérios: o conhecimento e habilidade da equipe multiprofissional com o modo; a disponibilidade de ventiladores e a indicação clínica, baseada principalmente na presença ou não de estímulo respiratório, da estabilidade hemodinâmica e da intensidade da lesão pulmonar.

### Pressão positiva expiratória final 

**Sugestão -** Em não havendo SDRA, utilizar valores de PEEP de 5 a 10cmH_2_O. O valor da PEEP deve ser ajustado em combinação com à fração inspirada de oxigênio (FiO_2_) para manter a SpO_2_ entre 90 e 95% para minimizar lesões cognitivas. Ventilação mecânica sem PEEP ou com PEEP muito baixa estão associadas a maior translocação bacteriana.^(151-155) ^Em havendo SDRA, vide tema específico destas recomendações. 

### Ajuste da fração inspirada de oxigênio 

**Sugestão -** O valor da FiO_2_ deve ser ajustado em combinação com a PEEP para manter a SpO_2_ entre 90 e 95% para minimizar lesões cognitivas. ^(^ 151 ^-^ 155 ^)^

### Volume corrente

**Sugestão -** Volumes correntes superiores a 6mL/kg de peso ideal aumentam a translocação bacteriana e a lesão pulmonar induzida pelo ventilador; portanto os pacientes devem ser ventilados com volumes correntes ≤6mL/kg de peso predito.^(^ 151 ^-^ 155 ^)^

### Decúbito

**Sugestão -** Em pacientes com pneumonia unilateral e hipoxemia grave, pode-se praticar a mudança para decúbitos laterais. No entanto, pelos resultados imprevisíveis, é necessária vigilância intensa, porque há risco de piora da oxigenação e de contaminação do pulmão contralateral.^(^ 156 ^)^

### Tratamento de resgate

**Sugestão -** Pacientes com pneumonia unilateral e hipoxemia refratária ao tratamento convencional podem ser candidatos à ventilação mecânica independente. No entanto, esse tratamento deve ser usado em centros com experiência em ventilação independente e com serviço de broncoscopia disponível.^(^ 157 ^)^

## Pneumonia associada à ventilação mecânica

**Sugestão -** Os pacientes com pneumonia associada à ventilação (PAV) devem ser ventilados utilizando-se estratégia ventilatória protetora (VC=6mL/kg peso predito); f visando manter PaCO_2_ entre 35 e 45mmHg e PEEP suficiente para garantir uma adequada troca gasosa, com modo ventilatório VCV ou PCV. Assim que possível, passar a modos assistidos ou espontâneos visando adiantar a retirada da VM.

**Sugestão -** Em pacientes com pneumonia unilateral e hipoxemia grave pode-se praticar a mudança para decúbitos laterais. No entanto, pelos resultados imprevisíveis, é necessária vigilância intensa, porque há risco de piora da oxigenação e de contaminação do pulmão contralateral. Estão em estudo novas propostas de posicionamento visando à prevenção da PAV, como decúbitos lateralizados em Trendelemburg, visando diminuir aspiração de secreção suprabalonete.^(^ 158 ^)^

**Recomendação** - Seguir as seguintes estratégias gerais para redução da PAV: lavagem das mãos e/ou desinfecção das mãos com base de álcool a 70%; uso de vigilância microbiológica; monitoramento e remoção precoce de dispositivos invasivos; programas para uso racional de antibióticos.

**Recomendação -** A troca de circuitos do ventilador deve ser feita apenas quando sujos ou danificados, não havendo necessidade de troca programada. 

**Recomendação -** A troca de umidificadores deve ser feita a cada 7 dias ou quando necessário.

**Recomendação -** Realizar aspiração de secreções subglóticas quando o paciente for permanecer por mais de 72 horas em ventilação mecânica, de forma intermitente ou controlada por dispositivo especialmente desenvolvido para esse fim.^(^ 159 ^)^

**Sugestão -** Quando disponível, usar cânulas com balonetes especialmente desenvolvidos para evitar microaspiração nos pacientes com previsão de ventilação mecânica por pelo menos 24 horas.

**Recomendação -** Colocar e monitorizar a pressão do balonete do tubo endotraqueal em pelo menos 25cmH_2_O.^(^ 160 ^)^

**Recomendação -** A cabeceira do leito deve ser mantida elevada, com angulação entre 30 a 45°.

**Recomendação -** Deve-se proceder a higiene oral diária com clorexedine 2%.^(^ 161 ^)^

**Sugestão -** Deve-se proceder a interrupção diária da sedação.

**Sugestão -** Deve-se proceder a descontaminação seletiva do trato digestivo. ^(^ 162 ^,^ 163 ^)^

**Recomendação -** Utilizar tubo traqueal revestido com prata quando intubação for prevista para mais de 24 horas.^(^ 164 ^)^

## Ventilação mecânica no paciente com sepse

**Comentário -** A SDRA é uma das complicações comumente observadas em pacientes com sepse grave, sendo, na maioria das vezes, subdiagnosticada. Estudos observacionais demonstram que apenas 30 a 50% dos pacientes que apresentam lesão alveolar difusa em autópsia tiveram o diagnóstico de SDRA documentado em prontuário médico.^(165-167) ^Daí a importância de atenção especial quanto à presença de SDRA entre pacientes com sepse. Há algumas intervenções de eficácia comprovada em pacientes com SDRA, como ventilação com volume corrente entre 4 e 6mL/kg de peso predito, as quais precisam ainda ser mais divulgadas, aplicadas e auditadas na prática clínica.^(168-170) ^A não realização do diagnóstico é possivelmente uma das razões para baixa adesão da instituição do tratamento correto.^(^ 166 ^)^

**Sugestão -** Empregar um sistema rotineiro de identificação de pacientes com SDRA nos pacientes com sepse, particularmente entre pacientes com sepse grave e choque séptico, possivelmente usando a queda da relação PaO_2_/FiO_2_ e infiltrado bilateral na radiografia de tórax como diagnósticos para SDRA e sinais clínicos^(171,172) ^(f aumentada, dessaturação e necessidade de uso de O_2_) como alertas precoces.

**Observação - **O diagnóstico e a condução do paciente com SDRA encontram-se nas seções "Ventilação mecânica na SDRA" e "Ventilação na posição prona e circulação extracorpórea" destas recomendações.

**Recomendação -** Utilizar volume corrente em torno de 6mL/kg de peso predito em pacientes com sepse sem SDRA, sob ventilação mecânica. Revisão sistemática incluindo estudos randomizados e observacionais, que envolveu tanto pacientes submetidos a cirurgias quanto pacientes internados em UTI, sugeriu que ventilação com volumes correntes baixos reduziu a mortalidade, incidência de SDRA e pneumonia, em comparação a volumes correntes altos.^(^ 52 ^)^

**Observação -** A assistência de pacientes sob ventilação mecânica com pneumonia e sepse, porém sem SDRA, encontra-se na seção "Ventilação Mecânica na Pneumonia".

## Ventilação mecânica na síndrome da angústia respiratória aguda ou síndrome do desconforto respiratório agudo: diagnóstico, recomendações e cuidados

**Comentário - **A partir de 2012, a SDRA passou a ser classificada (Definição de Berlim) como SDRA leve, moderada e grave^(173) ^(Quadro 6). 

**Qaudro 6:** Classificação de Berlim da sindrome de desconforto respiratório agudo(173) PaO2/FiO2 - relação entre pressão parcial de oxigênio e fração inspirada de oxigênio; PEEP - pressão expiratória final positiva; CPAP - pressão positiva contínua nas vias aéreas.

### Como ventilar o paciente com SDRA 

Modos ventilatórios

**Recomendação -** No início (primeiras 48 a 72 horas) do ajuste da ventilação mecânica em pacientes com SDRA (todas as categorias de gravidade - leve, moderada e grave), são recomendados modos controlados: VCV ou PCV. Em PCV, o valor da pressão das vias aéreas se equipara à pressão de platô ou pressão alveolar, quando o fluxo inspiratório cai a zero.

### Volume corrente(55,174,175)

**Recomendação -** Se SDRA leve, sob ventilação assistida, VC deve ser ajustado em 6mL/kg (considerando-se peso predito). 

**Recomendação -** Na SDRA moderada ou grave, sob ventilação assistida ou controlada, VC deve ser ajustado entre 3 e 6mL/kg (considerando-se peso predito).^(^ 176 ^)^

**Recomendação -** Para se obter o peso predito (*predicted body weight*) usar as seguintes fórmulas:^(^ 175 ^)^ homens : 50 + 0,91 x (altura em cm - 152,4); mulheres**:** 45,5 + 0,91 x (altura em cm - 152,4).

### Fração inspiratória de oxigênio 

**Recomendação -** Usar a menor FiO_2 _possível para garantir SpO_2_ >92% em todos as categorias de gravidade SDRA.

### Pressão de platô 

**Recomendação -** Buscar manter pressão de platô (Pplatô) ≤30cmH_2_O.^(175,177)^

**Recomendação -** Buscar manter o diferencial de pressão platô - PEEP (chamado de pressão de distensão, pressão motriz inspiratória ou *driving pressure*) ≤15cmH_2_O para todas as categorias de gravidade SDRA.^(91)^

**Sugestão -** Em casos de SDRA moderada e grave, quando a PEEP usada for elevada (geralmente >15cmH_2_O), pode-se tolerar Pplatô de no máximo 40cmH_2_O, desde que necessariamente a pressão de distensão seja mantida ≤15cmH_2_O. ^(^ 91 ^)^

### Frequência respiratória 

**Recomendação -** Iniciar com f=20rpm e, caso necessário, aumentar até 35 rpm, desde que não ocasione auto-PEEP, de acordo com a PaCO_2_ almejada (manter <80mmHg). Em casos de SDRA moderada ou grave, submetidos a estratégia de hipercapnia permissiva com VC ≤6mL/kg de peso predito, a f pode ser ajustada até 45rpm, desde que não ocasione auto-PEEP.^(^ 175 ^)^

### Ajuste da PEEP

**Comentário -** Existem várias formas de ajuste da PEEP na SDRA, muitas delas em situação de *equipoise* (grau de evidência que não permite conclusão definitiva sobre a superioridade de uma delas). A seguir descrevem-se as técnicas que exibem mais longa experiência e maior segurança em estudos clínicos.

**Recomendação -** Evitar utilizar PEEP <5cmH_2_O em paciente com SDRA.^(^ 55 ^,^ 175 ^)^

**Recomendação** - Evitar usar PEEP inferior aos valores da tabela PEEP BAIXO *versus* FIO_2_ (Tabela 1).^(175)^

**Tabela 1:** PEEP versus FiO2 para encontro da melhor PEEP, em situações de SDRA leve

FIO_2_	0.3	0.4	0.4	0.5	0.5	0.6	0.7	0.7	0.7	0.8	0.9	0.9	0.9	1.0
PEEP	5	5	8	8	10	10	10	12	14	14	14	16	18	18↔24

**Adaptado de:** : Ventilation with lower tidal volumes as compared with traditional tidal volumes for acute lung injury and the acute respiratory distress syndrome

The Acute Respiratory Distress Syndrome Network. N Engl J Med. 2000;342(18):1301-8. FiO2 - fração inspirada de oxigênio

PEEP - pressão expiratória final positiva.

**Sugestão -** Utilizar a tabela PEEP BAIXO *versus* FiO_2_ (Tabela 1) apenas em casos de SDRA leve. ^(175)^

**Comentário -** Há duas opções para ajuste de PEEP alto, segundo os estudos ALVEOLI^(178) ^e LOVS,^(^ 179 ^)^ disponíveis na tabela 2, com resultados práticos muito semelhantes. A tabela do LOVS tende a deixar o paciente mais tempo sob PEEP elevada.

**Tabela 2:** Ajuste da PEEP em parâmetros altos para encontro da melhor PEEP em SDRA moderada e grave

Tabela do estudo ALVEOLI																
FIO_2_	0.3	0.3	0.4		0.4		0.5	0.5		0.5↔0.8		0.8		0.9		1.0
PEEP	12	14	14		16		16	18		20		22		22		22↔24
**Tabela do estudo LOVS**																
FIO_2_	0.3	0.4		0.5		0.6			0.7		0.8		0.9		1.0	
PEEP	5↔10	10↔18		18↔20		20			20		20↔22		22		22↔24	

**Fonte:** : Baseada nos estudos ALVEOLI(178) e LOVS.(179) FiO2 - fração inspirada de oxigênio; PEEP - pressão expiratória final positiva.

**Sugestão -** Usar essas duas tabelas em casos de SDRA moderada e grave como alternativa à técnica da PEEP decremental, descrita adiante. 

**Sugestão -** O estudo Express sugere usar a Pplatô no máximo valor de 30cmH_2_O e nos casos de SDRA moderada e grave usar a PEEP máxima com VC 6mL/kg de peso predito.^(^ 180 ^)^

**Sugestão -** Evitar o uso dessas tabelas em caso de SDRA leve.^(^ 181 ^)^

### PEEP decremental, titulada pela complacência do sistema respiratório 

**Recomendação -** Sobre a técnica de PEEP decremental: Após manobra de recrutamento máximo (MRM), mede-se a complacência estática do sistema respiratório em valores decrementais de PEEP, a partir de valores de 23 a 26cmH_2_O, até valores mínimos ao redor de 8 a 12cmH_2_O. Os passos decrementais de PEEP são tipicamente de 2 ou 3cmH_2_O a cada 4 minutos. Após identificação da PEEP que produz a melhor complacência, ou de dois ou mais passos de PEEP com complacência equivalentes, escolhe-se uma PEEP 2 a 3cmH_2_O acima desse valor. Antes de finalmente ajustar o valor da PEEP para o obtido como adequado, procede-se a uma nova manobra de recrutamento máximo, após a qual a PEEP pode ser ajustada diretamente para 2 a 3cmH_2_O acima do valor encontrado na titulação decremental.^(^ 182 ^-^ 184 ^)^

**Sugestão -** Considerar essa manobra nos casos de SDRA moderada e grave.^(^ 182 ^-^ 184 ^)^

### PEEP decremental, titulada por outros métodos

**Sugestão -** Obter a PEEP decremental usando TIE nos centros que disponham da tecnologia: após manobra de máximo recrutamento, escolhe-se a PEEP decremental que resulta em menos de 0 a 5% de aumento do colapso, como estimado pela TIE.^(^ 95 ^)^

**Sugestão -** Obter a PEEP decremental usando tomografia computadorizada convencional. Após manobra de máximo recrutamento, escolhe-se a PEEP decremental que resulta em menos de 0 a 5% de aumento do colapso, como estimado pela TC. Esse método deve considerar todos os cuidados pertinentes com o deslocamento e a segurança do paciente, em equipes treinadas para esse tipo de procedimento, que deve ser feito com tomografia de baixa radiação.^(^ 91 ^)^

**Sugestão -** Pela oxigenação, após MRM, escolhe-se a PEEP decremental que resulta em <10% de queda na relação PaO_2_/FIO_2_.

### Obtenção do ponto de inflexão inferior pela técnica dos volumes aleatórios

**Recomendação** - Em paciente sedado, sem *drive* ventilatório ativo, fixar a PEEP em zero e variar o VC em alíquotas de 50mL, chegando a um valor máximo de VC de 1.000mL ou Pplatô de 40cmH_2_O, registrando a medida da Pplatô após três ventilações. Anotar os valores em uma tabela VC *versus* Pplatô e colocar num gráfico tipo XY (VC na ordenada e Pplatô na abscissa). Espera-se encontrar um traçado sigmoide. Tentar identificar, na curva, as tendências do traçado obtido, onde há o ponto de inflexão inferior (encontro das retas de tendência na primeira curvatura, projetando o valor na abscissa) e fixar a PEEP 2,0cm acima deste ponto.^(^ 55 ^)^

### Obtenção do ponto de melhor complacência (técnica da PEEP-complacência)

**Recomendação -** Em paciente sedado, sem *drive* ventilatório ativo, fixar o VC em 6mL/kg/peso predito e variar os valores de PEEP em alíquotas de 2 ou 3cmH_2_O, aguardando três ventilações para registrar a Pplatô. Anotar os valores em uma tabela PEEP *versus* complacência estática do sistema respiratório (Csr - cálculo da complacência descrito no item "Monitorização da ventilação mecânica" destas recomendações), visando encontrar o ponto de PEEP que proporcionou melhor Csr. Fixar a PEEP 2,0cm acima desse ponto. Em situação de se encontrarem dois valores melhores de Cst iguais, considerar como a PEEP ideal a de valor maior.

### Bloqueador neuromuscular

**Recomendação -** Nos casos de SDRA com relação pO_2_/FiO_2_<120mmHg sob sedação profunda, utilizar cisatracúrio nas primeiras 48 horas de suporte ventilatório.^(^ 111 ^)^

### Posição prona

**Recomendação -** Usar a posição prona em pacientes com SDRA com P/F <150 por pelo menos 16 horas por sessão. (Mais detalhes no tema específico destas recomendações).^(^ 185 ^)^

**Recomendação -** Deixar de ser repetida ventilação com posição PRONA assim que se atingir PaO_2_/FiO_2_>150mmHg com PEEP≤10cmH_2_O em posição supina.^(^ 17 ^)^

**Sugestão - **Na SDRA moderada ou grave, usar a posição prona em pacientes que apresentem disfunção do ventrículo direito (VD) com hipoxemia controlada; e nos pacientes com dificuldade de se manter a estratégia protetora dentro de limites de segurança (pressão de distensão ≤15cmH_2_O e pH >7,15).^(^ 185 ^,^ 186 ^)^

### Manobras de máximo recrutamento alveolar

**Sugestão -** Nos casos de SDRA moderada e grave, utilizar manobras de MRM como parte da estratégia protetora, com o objetivo de reduzir a pressão de distensão inspiratória (*driving pressure*), após ajuste da PEEP decremental.^(^ 91 ^,^ 187 ^)^

**Recomendação -** Sobre a técnica da MRM, ela deve ser realizada em modo PCV, com pressão de distensão de 15cmH_2_O. Iniciar com PEEP=10cmH_2_O, aumentando o valor da PEEP em incrementos de 5cmH_2_O a cada 2 minutos, até atingir um valor de 25cmH_2_O, após o qual aumenta-se o incremento para 10cm de H_2_O, atingindo 35 e, no máximo, 45cmH_2_O. Na sequência, baixar a PEEP para 25cmH_2_O e iniciar de manobra de titulação decremental da PEEP como descrita em item anterior.^(^ 91 ^,^ 187 ^)^

**Recomendação -** Instituir acesso venoso central e monitorização contínua de pressão arterial invasiva.^(^ 91 ^,^ 187 ^)^

**Recomendação -** Nos casos de hipoxemia refratária, não responsivas à posição prona, usar MRM, seguida de reajuste da PEEP pelo método decremental, com terapia de resgate nos pacientes elegíveis para essa técnica, dentro das normas de monitorização e segurança recomendadas nestas recomendações.^(^ 91 ^,^ 187 ^)^

### Ventilação com alta frequência

**Recomendação -** Deve-se evitar o uso de ventilação de alta frequência como terapia adjuvante.^(^ 188 ^)^

### Óxido nítrico 

**Sugestão -** Usar óxido nítrico (NO) inalatório em pacientes com SDRA grave com hipertensão pulmonar aguda e falência de VD, monitorizando-se a resposta, e titular a dosagem de partes por milhão (ppm).^(^ 187 ^)^

### Oxigenação extracorpórea por membrana (veno-venosa)

**Recomendação -** Nos casos de hipoxemia refratária definida como uma relação P/F <80mmHg, com FiO_2_>80%, após realização das manobras adjuvantes e de resgate para SDRA grave por pelo menos 3 horas, usar oxigenação por membrana extracorpórea (ECMO) veno-venosa nos centros com essa tecnologia disponível. Mais detalhes no tema específico nestas recomendações. ^(^ 187 ^)^

## Ventilação na posição prona e circulação extracorpórea

### Ventilação com posição prona: quando realizar?

**Recomendação -** Se indicada, realizar nas primeiras 48 horas de ventilação mecânica.^(^ 185 ^,^ 189 ^-^ 191 ^)^

### Indicações

**Recomendação -** Evitar utilizar na SDRA leve de forma rotineira.^(^ 185 ^,^ 189 ^-^ 191 ^)^

**Sugestão -** Utilizar na SDRA moderada,^(185,189-191) ^após PEEP titulada; se IVD (insuficiência ventricular direita aguda - cor pulmonale agudo) moderada a grave; na impossibilidade de sustentar ventilação protetora: necessário pressão de distensão >15cmH_2_O; f>35rpm; pH<7,2.

**Recomendação -** Deve ser usada posição prona precocemente (<48 horas do diagnóstico de SDRA) nos casos de SDRA com PaO_2_/FiO_2_ <150.^(^ 185 ^,^ 189 ^-^ 191 ^)^

### Durante quanto tempo manter pronado?

**Recomendação -** Manter a prona de 16 a 20 horas, com todos os cuidados adequados de proteção e monitorização.^(^ 185 ^,^ 189 ^-^ 191 ^)^

### Quais as contraindicações do uso da posição prona?

**Recomendação -** Contraindicar prona nas seguintes situações:^(^ 185 ^,^ 189 ^-^ 191 ^)^ hipertensão intracraniana; fratura pélvica; fratura de coluna; hipertensão intra-abdominal (contraindicação relativa); peritoniostomia; gestação (contraindicação relativa); tórax instável; instabilidade hemodinâmica grave; equipe inexperiente.

### Técnicas e cuidados na posição prona

**Recomendação** - Recomenda-se as seguintes técnicas e cuidados a serem respeitados:^(^ 185 ^,^ 189 ^-^ 191 ^)^ elevar FiO_2_ para 100% durante a rotação; se o paciente estiver ventilando em PCV, tomar cuidado com a queda de volume exalado; sedação e analgesia otimizadas; instituir acesso venoso central e monitorização contínua de pressão arterial invasiva; colocar travesseiros na frente do paciente distribuídos de modo a aliviar os pontos de apoio anatômicos principais; colocar coxins na cintura pélvica e escapular para alívio da compressão sobre o abdome; usar proteção para testa, face, joelhos e ombros (placas hidrocoloides); considerar colocar uma fralda absorvente na face do paciente; trocá-la, se muito úmida; monitorizar eletrocardiograma pelas costas; movimentar cabeça do paciente, principalmente modificando pontos de apoio na face, pelo menos a cada 2 horas; mudar posição de braços, acima e abaixo da linha interescapular pelo menos a cada 2 horas; manter dieta enteral com volume menor; certificar-se de que os olhos estão fechados; manter o posicionamento pelo tempo necessário desde que não existam sinais atribuídos à posição prona, de sofrimento cutâneo ou outro órgão; monitorar resposta à rotação com SpO_2_, se houver dessaturação <90% mantida após 10 minutos da rotação, retornar para supina; retornar a posição supina se parada cardiorrespiratória (PCR), piora hemodinâmica grave, arritmias malignas ou suspeita de deslocamento da prótese ventilatória; envolver de três a cinco pessoas para efetuar a rotação; treinar equipe e usar para isso vídeos constantes na literatura como nos sites a seguir: (PROSEVA - três pessoas - http://www.youtube.com/watch?v=E_6jT9R7WJs e William Harvey Hospital - cinco pessoas - http://www.youtube.com/watch?v=Hd5o4ldp3c0); coletar gasometria após 1 hora de prona, considerar o paciente como respondedor se a relação pO_2_/FiO_2_ aumentar em 20 ou PaO_2_ aumentar em 10mmHg.

### Troca gasosa extracorpórea

**Comentário -** A assistência pulmonar extracorpórea pode ser utilizada para remoção de CO_2_ ou para oxigenação por membrana extracorpórea.^(^ 179 ^,^ 180 ^,^ 192 ^,^ 193 ^)^

### Quais as indicações de troca gasosa extracorpórea?

**Recomendação - Os **critérios obrigatórios de indicação de troca gasosa extracorpórea incluem: intubação traqueal e em ventilação mecânica; pacientes ≥18 anos; doença pulmonar de início agudo; possibilidade de reversão da lesão pulmonar (pacientes com doença pulmonar irreversível em espera para transplante têm indicação sugerida em centros com essa disponibilidade); SDRA com PEEP≥10cmH_2_O; realização das técnicas em centro com experiência. São critérios complementares (há a necessidade pelo menos um): relacionado à hipoxemia - relação PaO_2_/FiO_2_≤80 com FiO_2_≥0,8 por pelo menos 3 horas, apesar da realização de manobras de resgate; relacionado à hipercapnia - hipercapnia com manutenção do pH≤7,20 com f de 35rpm e VC entre 4 a 6mL/kg de peso predito, obrigatoriamente com pressão de distensão ≤15cmH_2_O

### Quais as contraindicações de troca gasosa extracorpórea?

**Recomendação** - Recomenda-se contraindicar troca gasosa extracorpórea em pacientes moribundos; pacientes com índice de massa corporal >40 a 45; coma (paciente sem sedativos) após PCR; pacientes pneumopatas crônicos sem reversibilidade da doença - exceto em pacientes com possibilidade de transplante; pacientes sem acesso vascular calibroso, seguro e acessível; doença crônica limitante sem perspectiva; trombocitopenia induzida por heparina (HIT). ^(^ 179 ^,^ 180 ^,^ 192 ^,^ 193 ^)^

### Quais os dispositivos usados na troca gasosa extracorpórea?

**Sugestão -** Para realizar remoção de CO_2_ sugere-se a assistência pulmonar intervencionista (ILA) arteriovenosa (A-V) nos casos sem instabilidade hemodinâmica.

**Sugestão -** Para se realizar a oxigenação e remoção de CO_2_ sugere-se assistência circulatória com oxigenação por membrana extracorpórea (ECMO). 

### Oxigenação extracorpórea por membrana - técnicas e cuidados

**Recomendação** - Aplicar as seguintes técnicas e cuidados na utilização da ECMO: utilização de membrana composta por polimetilpentano tanto para remoção de CO_2_ quanto para ECMO; realizar em equipe com experiência (conhecer o sistema, conhecer a fisiologia, conhecer complicações mais comuns e como solucioná-las); evitar improvisações; usar acessos venosos e percutâneos como primeira escolha; Usar preferencialmente cânulas >18 Fr; se acesso arterial for necessário e o calibre da artéria não for maior que 4mm que o calibre da cânula considerar fortemente uma cânula de perfusão distal, antes da canulação proximal; prover anticoagulação segura ao paciente: monitorização de PTTa e plaquetometria a cada 6 horas; ventilar o paciente inicialmente de forma ultraprotetora: ventilação controlada com FiO_2_<0,6; PEEP=10cmH_2_O, pressão de distensão de 10cmH_2_O e/ou VC<4mL/kg/peso predito; frequência respiratória de dez inspirações por minuto.^(^ 179 ^,^ 180 ^,^ 192 ^,^ 193 ^)^

**Recomendação -** Na ECMO veno-venosa manter a FiO_2_ da ECMO em 1, e o menor fluxo de sangue necessário para manter uma saturação arterial >90%; manter um fluxo de ventilação da membrana para um pH entre 7,35 e 7,40. 

**Recomendação -** Quando em PSV, objetivar o menor trabalho respiratório respeitando a sincronia paciente *versus* ventilador sustentando parâmetros ventilatórios protetores (pressão de distensão <15cmH_2_O); 

**Recomendação -** Em paciente com fluxo de sangue maior que 5000 - 6000ml/minuto e SatO_2_<85% considerar conforme apropriado: aumento da FiO_2_ do ventilador; controle de agitação; verificação e correção de recirculação; controle de temperatura sistêmica; aumento da PEEP; sedação profunda e bloqueio neuromuscular; recrutamento alveolar e outros tais como betabloqueador, óxido nítrico (NO), prona e hipoxemia permissiva.

### Assistência pulmonar intervencionista - técnicas e cuidados

**Recomendação -** Usar as seguintes técnicas e cuidados na utilização da ILA: realizar eco-doppler para definir diâmetro arterial e venoso femoral; na inserção utilizar técnica guiada por ultrassom; garantir débito cardíaco e pressão de perfusão no sistema (pressão arterial média >70mmHg); monitorar fluxo no sistema de forma contínua com ultrassom; iniciar a titulação de O_2_ a 1L/min e não ultrapassar 10L/min; monitorar pH arterial conforme remoção de CO_2_ principalmente em pacientes com hipertensão intracraniana; manter ventilação protetora conforme descrito anteriormente em ECMO; prover anticoagulação segura ao paciente: monitorização de PTTa, fibrinogenemia e plaquetometria pelo menos a cada 6 horas; ponderar remoção sob visão direta (intervenção cirúrgica) objetivando redução de complicações vasculares.^(^ 179 ^,^ 180 ^,^ 192 ^,^ 193 ^)^

## Tecnicas adjuvantes

### Óxido nítrico

**Comentário -** Utiliza-se visando ajustar a relação ventilação/perfusão por meio de vasodilatação em território arterial pulmonar em áreas ventiladas.

### **Recomendação -** Não utilizar de forma rotineira

**Sugestão** - Pode ser utilizado na presença de *Cor pulmonale* agudo, hipoxemia grave e refratária.

**Recomendação - **Para utilização de NO, deve-se adotar as seguintes técnicas e cuidados: utilizar cilindro de NO acoplado a um sistema fechado próprio, e com monitor de NO inalado e NO_2_; usar dose inicial de 5ppm; manter NO_2_ <10ppm; usar monitorização hemodinâmica preferencialmente invasiva com cateter de termodiluição; monitorizar meta-hemoglobinemia e função renal; não utilizar em pacientes com deficiência de meta-hemoglobina redutase; não permitir cuidados no paciente por profissionais gestantes.^(^ 194 ^)^

### Heliox

**Comentário -** Utiliza-se heliox visando-se a redução da resistência em vias aéreas e redução do trabalho respiratório.

**Sugestão - **O heliox pode ser usado em doenças relacionadas a obstrução de via aérea inferior, para facilitar a manutenção do suporte ventilatório seja de forma não invasiva ou invasiva.^(^ 195 ^,^ 196 ^)^

**Recomendação - **As seguintes técnicas e cuidados devem ser empregados: ter disponível o material necessário (**v**entilador preparado para a utilização de heliox, regulador de heliox, oxímetro de gás, dois cilindros de heliox, sendo um para reserva, com concentração não inferior a 60/40), suspender em caso de hipoxemia grave e não postergar intubação conforme recomendações de falha de VNI.^(^ 195 ^)^

### Insuflação traqueal de gás contínua 

**Comentário -** A insuflação traqueal de gás contínua (TGI) visa retirar o CO_2_ do gás do espaço morto anatômico, diminuindo a hipercapnia para PaCO_2_<80mmHg. É um recurso que pode ser usado em situações de Pplatô>30cmH_2_O com VC baixos e PaCO_2_>80mmHg.

**Sugestão -** Para pacientes em que a f, C e as pressões em vias aéreas estão no limite de proteção e segurança do aparelho respiratório, mas com a PaCO_2_>80mmHg e/ou pH<7,2.

**Recomendação - **As seguintes técnicas e cuidados são recomendadas: usar capnografia com mensuração de ETCO_2_, levando-se em conta que TGI tem maior eficácia em pacientes com ETCO_2_ alto e próximo ao PaCO_2_ arterial; usar conector de broncoscopia para cânula traqueal e sonda fina (6Fr), através do conector; deixar a ponta do cateter 2 a 3cm acima da carina e abaixo da extremidade distal da prótese ventilatória (medir em tubo traqueal fora da traqueia); usar fluxo na TGI para que a linha de platô (agora descendente) do CO_2_ expirado chegue próximo ou toque a linha do zero; evitar fluxos >10L/min; realizar a TGI no modo PCV; ter em mente que os volumes medidos pelo ventilador serão inacurados; considerar que a pressão de platô não pode ser medida de forma acurada durante a TGI.^(^ 197 ^)^
